# Optimizing Shape Complementarity Enables the Discovery of Potent Tricyclic BCL6 Inhibitors

**DOI:** 10.1021/acs.jmedchem.1c02174

**Published:** 2022-06-03

**Authors:** Owen A. Davis, Kwai-Ming J. Cheung, Alfie Brennan, Matthew G. Lloyd, Matthew J. Rodrigues, Olivier A. Pierrat, Gavin W. Collie, Yann-Vaï Le Bihan, Rosemary Huckvale, Alice C. Harnden, Ana Varela, Michael D. Bright, Paul Eve, Angela Hayes, Alan T. Henley, Michael D. Carter, P. Craig McAndrew, Rachel Talbot, Rosemary Burke, Rob L. M. van Montfort, Florence I. Raynaud, Olivia W. Rossanese, Mirco Meniconi, Benjamin R. Bellenie, Swen Hoelder

**Affiliations:** † Cancer Research UK Cancer Therapeutics Unit, The Institute of Cancer Research, London SM2 5NG, U.K..; ‡ Division of Structural Biology, The Institute of Cancer Research, London SM2 5NG, U.K..

## Abstract

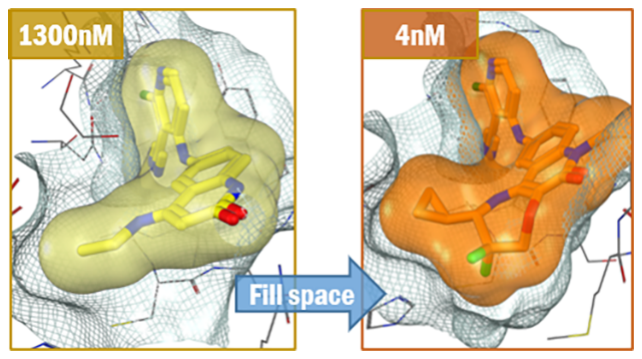

To identify new chemical series with enhanced binding affinity to the BTB domain of B-cell lymphoma 6 protein, we targeted a subpocket adjacent to Val18. With no opportunities for strong polar interactions, we focused on attaining close shape complementarity by ring fusion onto our quinolinone lead series. Following exploration of different sized rings, we identified a conformationally restricted core which optimally filled the available space, leading to potent BCL6 inhibitors. Through X-ray structure-guided design, combined with efficient synthetic chemistry to make the resulting novel core structures, a >300-fold improvement in activity was obtained by the addition of seven heavy atoms.

## Introduction

BCL6 (B-cell lymphoma 6 protein) is a master regulator of the germinal center (GC) B-cell phenotype and is required for GC formation.^1−3^ By binding to and repressing target genes, BCL6 attenuates cell differentiation and the DNA-damage response, thereby facilitating the process of somatic hypermutation required for antibody maturation.^4,5^ This phenotype broadly resembles that of malignant cells, so perhaps unsurprisingly, most B-cell lymphomas, including diffuse large B-cell lymphoma (DLBCL), derive from GC B-cells. Many of these tumors remain dependent on BCL6 expression for their survival.^6^

Transcriptional repression by BCL6 requires binding of corepressors including BCOR and NCOR to the BTB domain of BCL6.^7^ Disruption of this protein–protein interaction hence relieves BCL6-mediated gene repression. Both inhibitors and degraders of BCL6 have been shown to cause selective growth inhibition in BCL6-driven lymphoma cell lines,^8,9^ but to date, the therapeutic potential of inhibition or degradation of BCL6 in vivo in lymphomas has not been thoroughly tested. Improved tool compounds with suitable pharmacokinetic properties along with strong binding affinity are required to support this objective.

**Figure 1:**
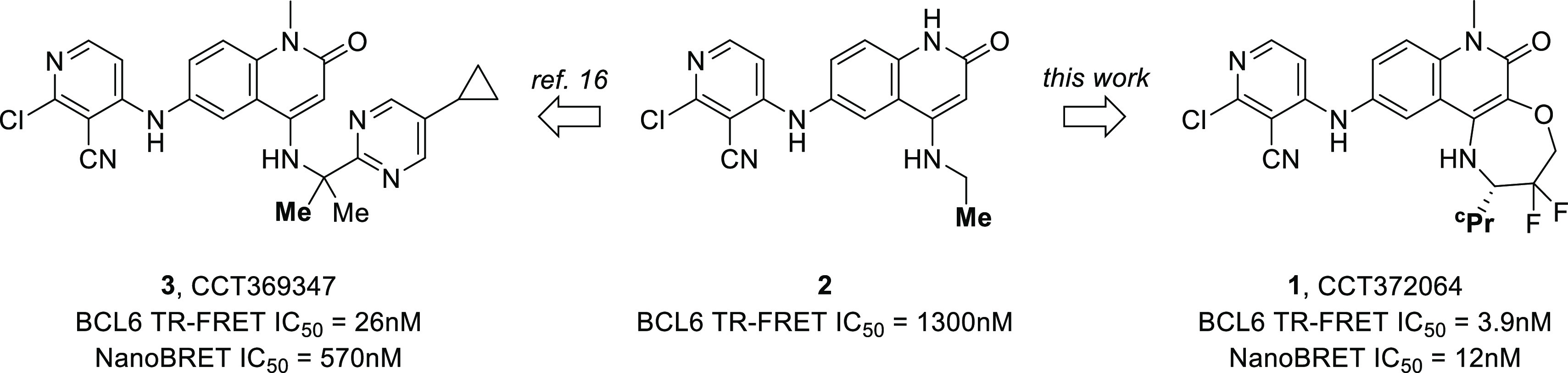
Potency improvements gained in the HDCH site through growing and cyclization strategies. The methyl or cyclopropyl group shown in bold occupies a hydrophobic region close to Val18.

Previously, both we and others have described compounds which bind to the BCL6 BTB domain and displace corepressors.^8−14^ Although the corepressors bind along a long, extended groove formed by dimerization of the BTB domain,^5,7^ reported hit compounds all bind in the same small area and form common interactions in the binding site: aromatic/hydrophobic contacts with Tyr58 and hydrogen bonding to the backbone C=O of Met51 and the backbone NH of Glu115. From these starting points, various approaches have been used to reach the high binding affinity required, including conformational restriction via macrocyclization^13^ and adding a further backbone H-bond interaction.^9^ In this study, we focus on further filling a largely hydrophobic subpocket defined by residues including His14, Asp17, Val18, and Cys53 (the “HDCH site”^15^) by fusing an additional ring onto a previously identified series of quinolinone inhibitors.^16^ Our design strategy focused on filling space and obtaining a close shape complementary to the protein surface. Progress was enabled by the development of short, efficient synthetic routes to access a variety of novel core structures designed using X-ray structural information. Our resulting lead compound **1** (CCT372064) shows a >300-fold improvement in activity compared with starting point **2** (Figure 1), gained entirely through improved binding in the HDCH site without modification to the 3-chloro-4-cyanopyridine substituent. In our subsequent paper, we report how the novel tricyclic core of **1** provided the basis for the design of a potent degrader of BCL6 suitable for testing the therapeutic hypothesis in vivo.

## Results and Discussion

We previously reported the identification of the quinolinone hit compound **2**, in which the terminal methyl of the *N*-ethyl group occupies a hydrophobic pocket close to Val18 in the HDCH site. Maintaining this methyl group in place, we further optimized affinity by growing the molecule into this broad binding pocket, displacing or interacting with bound water molecules.^16^ Although our resulting lead compound **3** (CCT369347) showed good potency of 26 nM in the biochemical time-resolved fluorescence energy transfer (TR-FRET) assay (Figure 1), we saw a substantial drop-off in the cellular nanoBRET assay (IC_50_ 570 nM), despite high passive permeability.^16^ Our aim was therefore to discover a chemical scaffold with improved binding affinity for the BTB domain of BCL6, which we expected in turn would lead to more potent activity in cellular assays.

**Figure 2:**
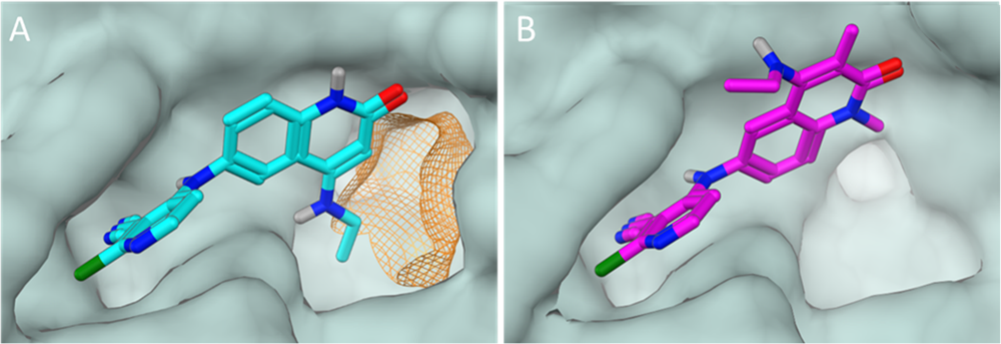
(A) X-ray structure of the BCL6 BTB domain with bound ligand **2** (PDB: 7OKH, cyan) showing the shape of the pocket (protein surface shown in gray). The unfilled pocket space potentially accessible from the quinolinone 3-position is highlighted in orange. (B) X-ray structure of the BCL6 BTB domain with bound ligand **4** (PDB: 7Q7S, magenta). The addition of a 3-quinolinone methyl group causes the 4-ethylamino group to twist, becoming orthogonal to the quinolinone ring. As a result, the quinolinone ring is forced to adopt a different binding mode, pointing the 4-substituent out to the solvent.

We hence inspected the X-ray structure of **2** (Figure 2A) for opportunities to design in additional interactions. We noticed that adding a substituent in the 3-position of the quinolinone ring may enable access to an unfilled region of the pocket. However, introducing such a substituent would be expected to cause a change in conformation for the 4-amino substituent to avoid steric clash. This was confirmed by synthesis of **4** (Table 1) and subsequent solving of its BCL6-bound crystal structure. This structure showed that the 3-methyl group induces rotation of the *N*-ethyl group (Figure 2B). As this alternate conformation cannot be accommodated by the protein, the knock-on effect is a “flip” of the whole quinolinone ring system which points the 4-amino group out to the solvent, resulting in a 4-fold reduction in biochemical potency.

**Table 1:**
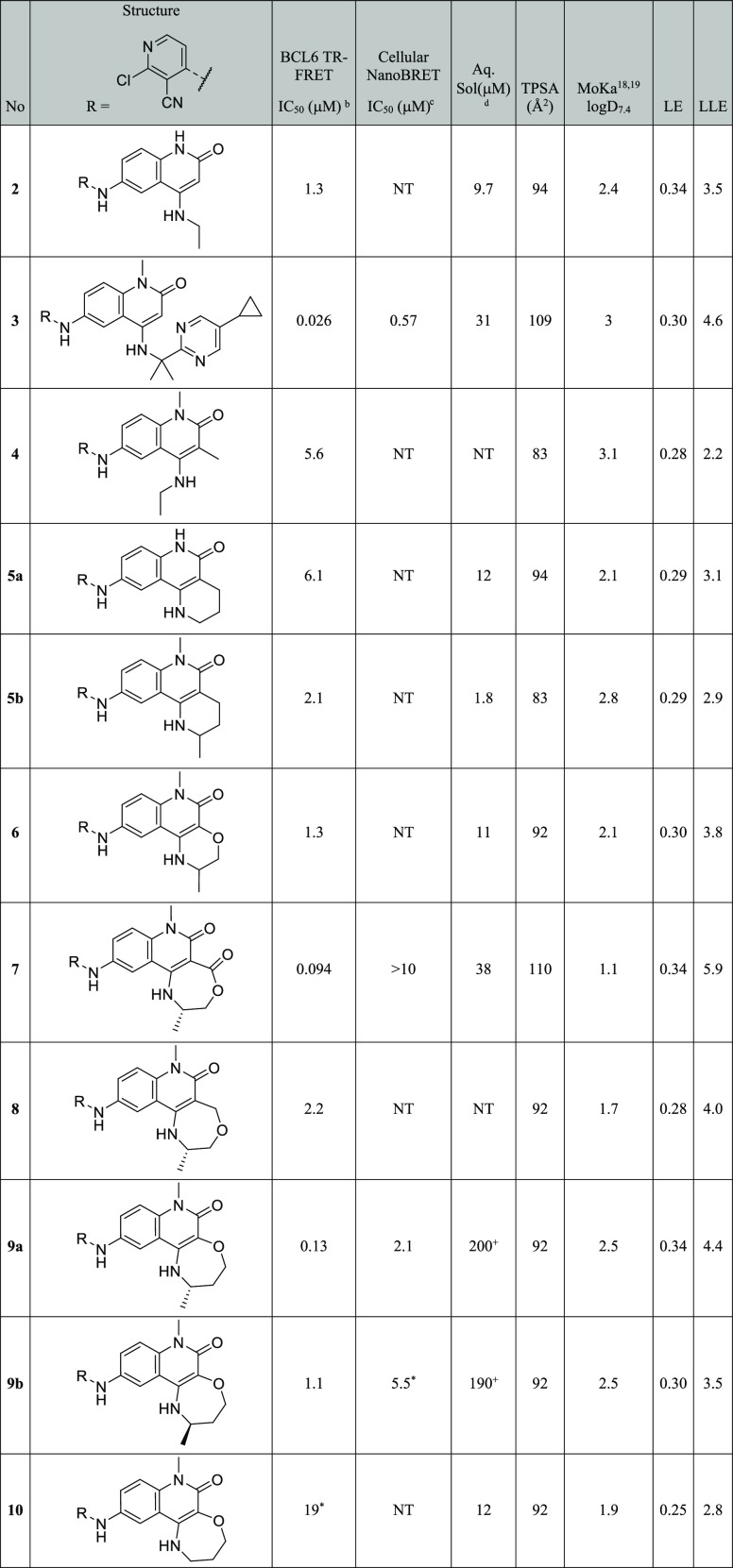
Biochemical and Cellular Data for 4-Aminoquinolinones and Fused-Tricyclic Derivatives^a^^18,19^

a Individual replicates and errors are shown in Table S1. NT = not tested.

b Quoted values represent the geometric mean of at least three replicates. *n* = 2 where indicated by *.

c Quoted values represent the geometric mean of two replicates. *n* = 1 where indicated by *.

d Kinetic solubility measured by HPLC in 10 mM phosphate-buffered saline buffer at pH 7.4 containing 1% DMSO. Where + shown, solubility was measured by NMR in HEPES buffer at pH 8, containing 4% DMSO.

To overcome this problem, we constrained 3- and 4-quinolinone substitution into a fused ring. We targeted the synthesis of a set of six- and seven-membered fused quinolinones to control the conformation of the quinolinone 3- and 4-substituents, aiming to more fully occupy the space in this region of the pocket (Table 1). Each of these novel cores required development of new synthetic routes, as described in more detail in the “synthesis” section below.

The IC_50_ values for these compounds are reported in Table 1. Compound **5a** shows no improvement over **4**, and we thus speculate that this may be adopting the same “flipped” binding mode. A methyl group was added to mimic the terminal methyl of **2** and hence maintain the interaction with the Val18 hydrophobic pocket. The resulting compound **5b** and ether analogue **6** showed >3-fold potency improvement over **5a**, but their activity was still no better than acyclic compound **2**. In contrast, our first seven-membered ring derivative **7** showed a >10-fold potency increase over **2** despite a reduction in lipophilicity. The large increase in lipophilic ligand efficiency (LLE) from 3.5 to 5.9 suggested that new interactions were likely being formed.^17^

**Figure 3:**
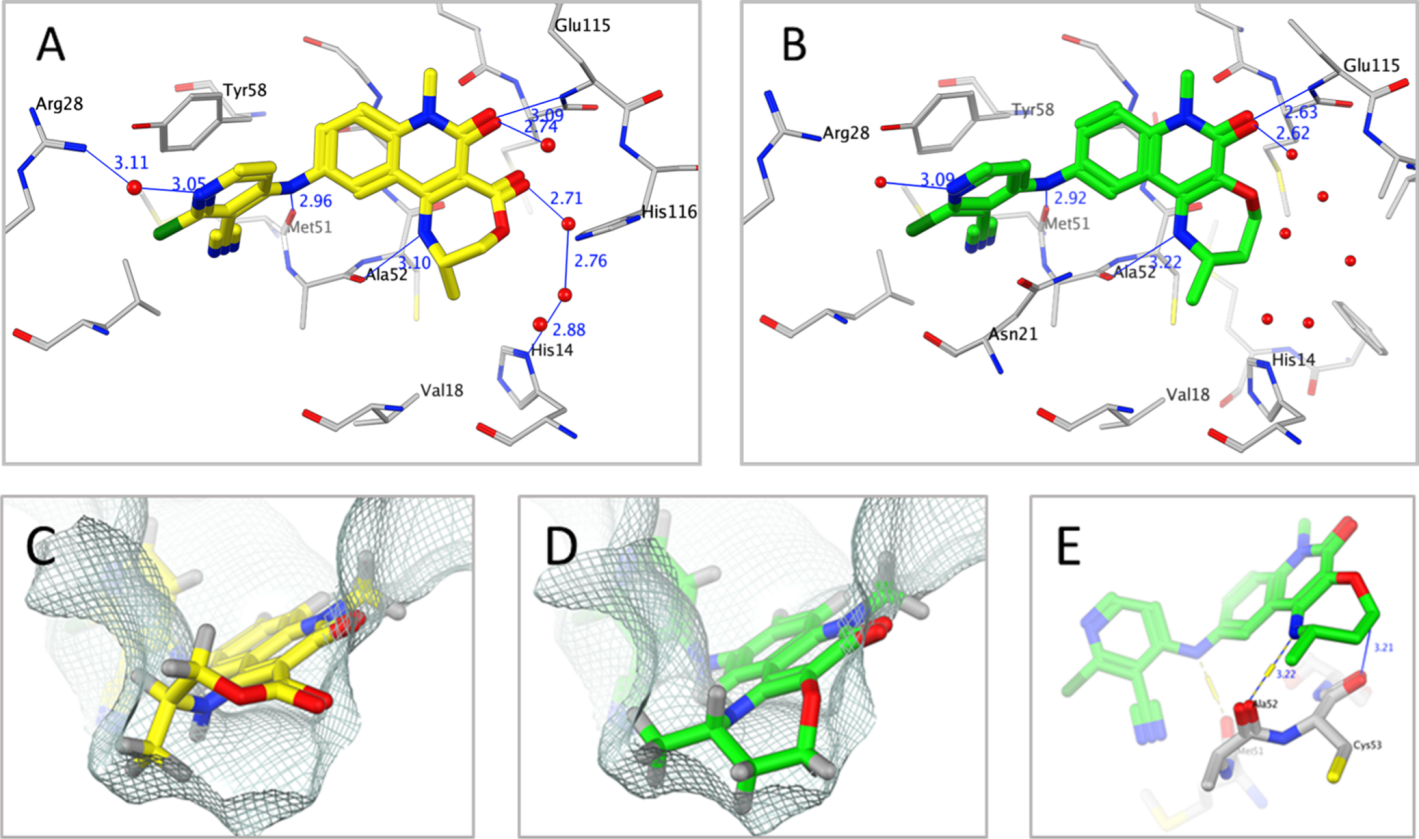
X-ray structures of the BCL6 BTB domain with bound ligands **7** [(A,C); yellow; PDB: 7Q7T] and **9a** [(B,D,E); green; PDB: 7Q7U]. The interaction surface [line, calculated using MOE (Chemical Computing Group)] is a zero-potential contour of the van der Waals potential of a probe atom—atoms sitting on the surface are at optimal distance for vdW interactions with the protein. Hydrogen atoms on the seven-membered ring of **9a** sit on this surface, showing that the pocket is well filled in this area (D), whereas the lactone ring of **7** (C) does not map closely to the surface, although it does form an additional strong H-bond to a water molecule (A). The carbonyl of Cys53 sits close to the ring carbon alpha to the ether oxygen of **9a** (E). Replacing this carbon with an oxygen (as in **8**) may be disfavored due to clashing lone pairs, preventing close surface contact.

We solved an X-ray structure of **7** bound to BCL6 (Figure 3A), which showed the same key interactions seen previously in the 4-aminoquinolinone series;^16^ the cyano-chloro-pyridine ring is clamped between Tyr58 and Asn21, forming a π–π interaction with Tyr58; there are H-bonding interactions to the backbone amides of Met51, Ala52, and Glu115; and the methyl group of the seven-membered ring sits in the lipophilic region of the pocket near Val18. In addition, a new interaction was observed between the lactone carbonyl and a conserved water molecule deep in the HDCH pocket. This water forms part of a network with two other water molecules, which in turn contact the main chain carbonyl of Cys53 and the His14 side chain.

Despite the high biochemical activity, no activity was observed in cells using the NanoBRET assay. We believed that this was due to the very low permeability (PAMPA *P*_e_ <0.2 × 10^–6^ cm s^–1^ at pH 7.4), driven in turn by the low hydrophobicity of **7** [clog*D*_7.4_ 1.1, topological polar surface area (TPSA) 109 Å^2^]. We decided to investigate the removal of the carbonyl in the seven-membered ring and prepared compound **8** (clog*D*_7.4_ 1.7, TPSA 92 Å^2^). Disappointingly, this change also removed most of the activity of the compound (Table 1). To understand the reason for this loss of activity, we reinspected the crystal structure of **7** and noted that the ring appeared to sit across the top of the pocket rather than align closely to the surface (Figure 3C). We hypothesized that this could be due to the backbone carbonyl of Cys53 at the back of the pocket, which could repel the ring oxygen of both lactone **7** and ether **8**. Moreover, we reasoned that moving the oxygen by one position—as in compound **9a**—would remove that repulsion and allow the seven-membered ring to form closer contacts with the protein surface, resulting in improved activity. Gratifyingly, **9a** showed a significantly increased TR-FRET activity of ∼100 nM, while having a much lower TPSA (92 Å^2^) and increased lipophilicity (Mo Kα log *D*_7.4_ 2.5) compared to **7**. This profile translated into a measurable, albeit modest, cellular activity (2 μM) in NanoBRET for **9a**.

X-ray crystallography confirmed our hypothesis that ether tricycle **9a** adopts a significantly different conformation to lactone tricycle **7** (Figure 3). While both positioned the terminal methyl group in the same location near Val18, the ether tricycle more closely follows the surface of the pocket, with the methylene group alpha to oxygen sitting 3.2 Å away from the carbonyl of Cys53 (Figure 3E). We hypothesized that the resulting displacement of an additional water molecule by **9a** and the hydrophobic contacts formed were able to compensate for the loss of the polar water-mediated interaction made by the carbonyl of **7**. Switching to methyl epimer **9b** or des-methyl **10** led to weaker activity in the TR-FRET assay, again reinforcing the importance of positioning the terminal methyl group in the Val18 pocket.

**Figure 4:**
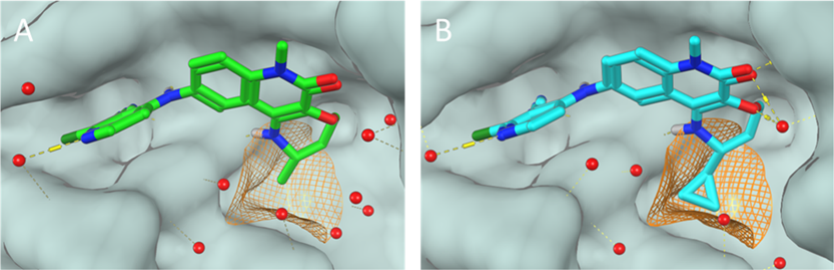
X-ray structures of the BCL6 BTB domain with bound ligands **9a** [(A); green; PDB: 7Q7U] and **12a** [(B); cyan; PDB: 7Q7V]. Molecular surface shown in gray. The interaction surface around the methyl group (orange grid, see Figure 2 legend for details) shows a triangular shape (A), which the cyclopropyl group of **12a** fills effectively (B).

Having identified a scaffold that effectively shape-matches the available space, we looked for opportunities to further optimize the potency. From the crystal structure of **9a**, it appeared that there was additional space around the methyl group close to Val18 that could be exploited (Figure 4A). This space appeared to be “triangular” in shape, and we speculated that a small alkyl or cycloalkyl group might fit into this pocket; we prepared a range of such groups to test this hypothesis. Racemic analogues were prepared initially due to building block availability, and the results are shown in Table 2.

**Table 2:**
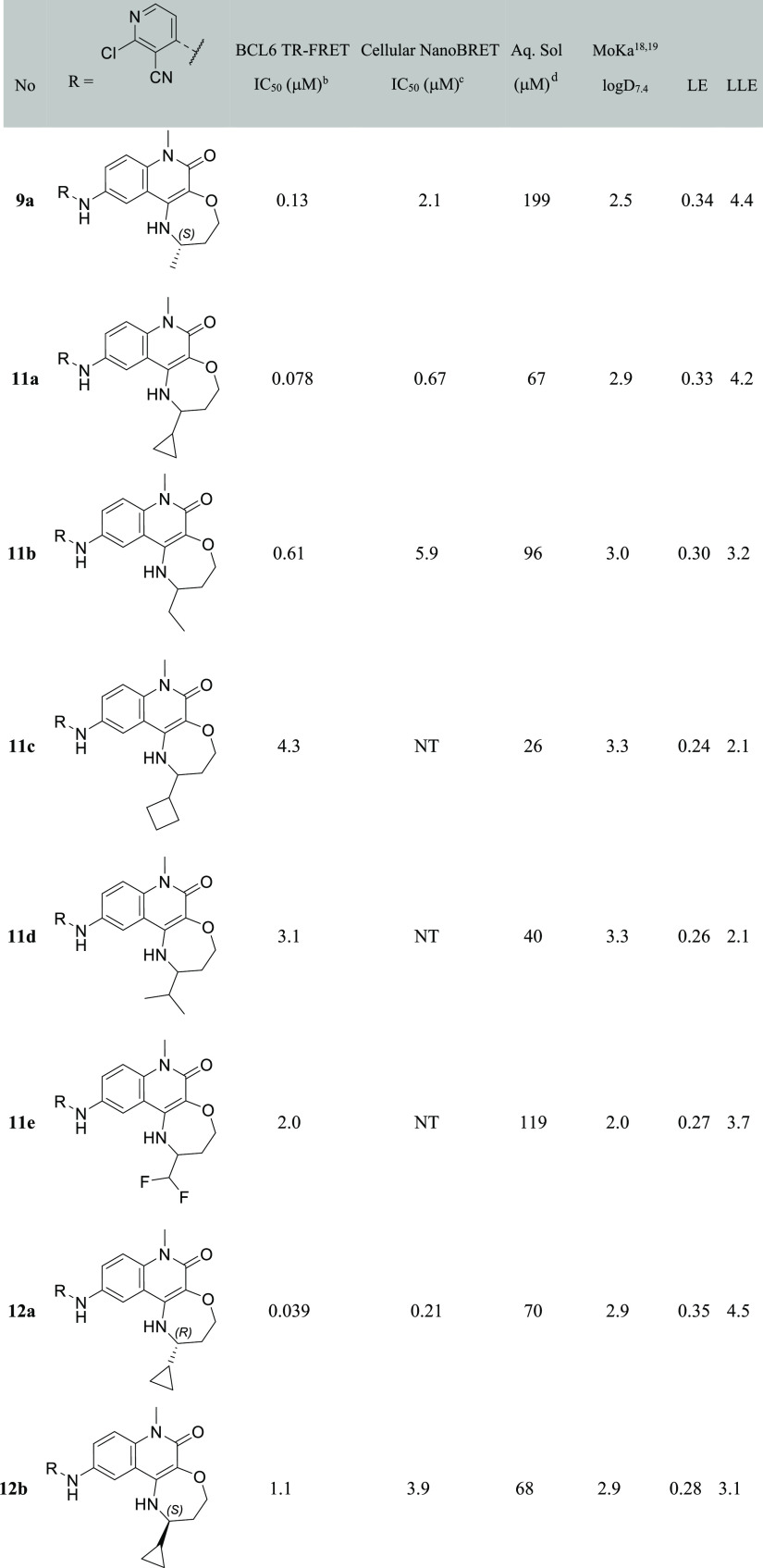
Biochemical and Cellular Data for Fused-7-Ring Tricyclic Quinolinones with Differing Groups in the Pocket Adjacent to Val18^a^

a Individual replicates and errors are shown in Table S1. NT = not tested.

b Quoted values represent the geometric mean of at least three replicates.

c Quoted values represent the geometric mean of at least two replicates.

d Kinetic solubility measured by NMR in HEPES buffer at pH 8, containing 4% DMSO.

Consistent with the pocket shape, cyclopropyl **11a** provided the optimal solution, with both smaller (ethyl, **11b**) and larger (cyclobutyl **11c** and isopropyl **11d**) substituents showing 8- to 55-fold reduction in activity in comparison. This highlights the importance of matching the geometric requirements of the pocket. Cyclobutyl and isopropyl substituents were not well tolerated as they do not fit into the triangular pocket—the larger bond angles (90–109.5°) between the methyl or methylene groups would lead to a clash with the protein. Reducing the size of this substituent by replacing each of the methyl groups of the isopropyl group with fluorine (**11e**) did not significantly recover this lost activity.

Single enantiomers of the cyclopropyl derivative were prepared, and as expected, the preferred enantiomer was (*R*)-cyclopropyl **12a**, in which the cyclopropyl points in the same direction as the (*S*)-methyl in **9a**. Although the potency gain from methyl to cyclopropyl is modest (∼3-fold), this improvement combined with a lipophilicity-driven increase in permeability led to a 10-fold increase in cellular assay activity for **12a** compared to **9a**.

The crystal structure of **12a** was solved and showed the same binding conformation as **9a** (Figures 4, 6). This supports our finding that the cyclopropyl group is the optimal solution in this region; it efficiently fills the additional “triangular” space, closely mapping to the surface.

**Figure 5:**
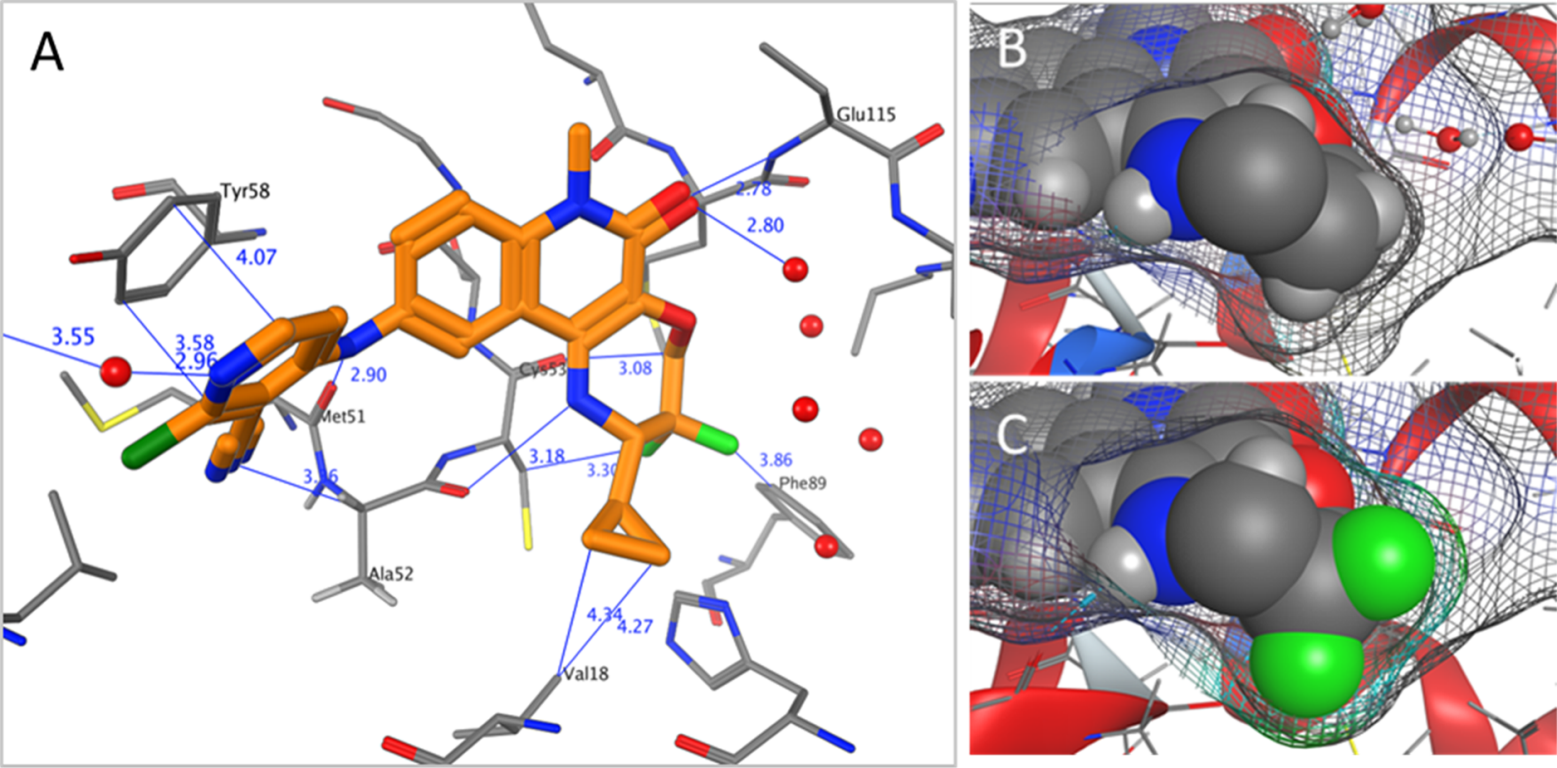
X-ray structure of the BCL6 BTB domain with bound ligand **1** [(A); orange; PDB 7Q7R], highlighting key distances between the protein and the ligand. Possible interactions observed include H-bonds with Met51 (backbone C=O), Glu155 (backbone NH), and Ala52 (backbone C=O); C–H···O interactions (Sutor bonds) with Cys53 (backbone C=O); vdW or dipole–dipole contacts between “δ−” fluorine atoms and “δ+” hydrogen atoms on Phe89 and Cys53; and vdW contacts with Val18 and π–π interactions between the electron-deficient pyridine ring and the electron-rich Tyr58 side chain. Close-up views of the CF_2_ group of **1** (C) show improved surface contact compared with the analogous CH_2_ group of **12a** (B). The cyclopropyl group has been truncated to methyl in (B,C) to enable a clear view of this region.

**Figure 6:**
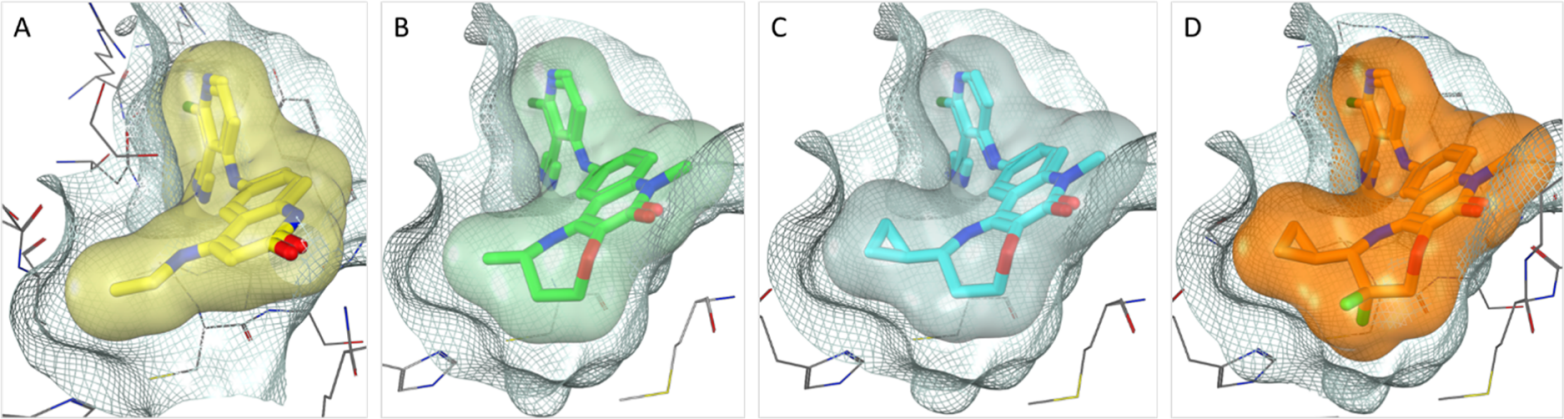
X-ray structures of the BCL6 BTB domain with bound ligand **1** [(D); orange] compared to **2** [(A); yellow], **9a** [(B); green], and **12a** [(C), cyan]. The protein surface is represented as a gray grid, and the ligand surface is shown in colors as above. The space in the pocket is more completely filled as potency is optimized from (A) through to (D).

Having identified the cyclopropyl ring as the optimal substituent adjacent to NH, we explored other positions of the seven-membered ring where the crystal structures showed potential for increasing the hydrophobic surface contact area (Table 3). First, methyl groups were incorporated alpha to the ether oxygen of **9a**, aiming to fill a small hydrophobic area. However, no potency improvement was observed for monomethyl **13a**, and dimethyl analogue **13b** showed a dramatic drop-off in potency, indicating that the space in this area was limited. Dimethyl or cyclopropyl *beta* to the oxygen (**13c** and **13d**, respectively) also reduced TR-FRET activity to micromolar levels, suggesting that these groups were too large. We therefore targeted difluoro substitution as a more subtle change. Adding difluoro substitution to **12a** led to a further potency breakthrough, with **1** (CCT372064) showing 4 nM activity in TR-FRET. This represents a 10-fold improvement for the addition of 2 fluorine atoms. This is a larger increase than would be expected from lipophilicity alone, as demonstrated by the increase in LLE from 4.5 (for **12a**) to 5.4 (for **1**). This can be explained by the improved shape matching between the ligand and the protein observed in the X-ray structure of **1** (Figures 5, 6). The electronegative fluorine atoms map closely to the protein surface, pointing toward a C–H bond in the Cys53 side chain and the aryl C–H of Phe89. In addition, the short distance (3.1 Å) between the backbone carbonyl of Cys53 and the ring CH_2_ suggests a possible Sutor bond,^20−22^ favored by the electron-withdrawing nature of the adjacent CF_2_ group.

**Table 3:**
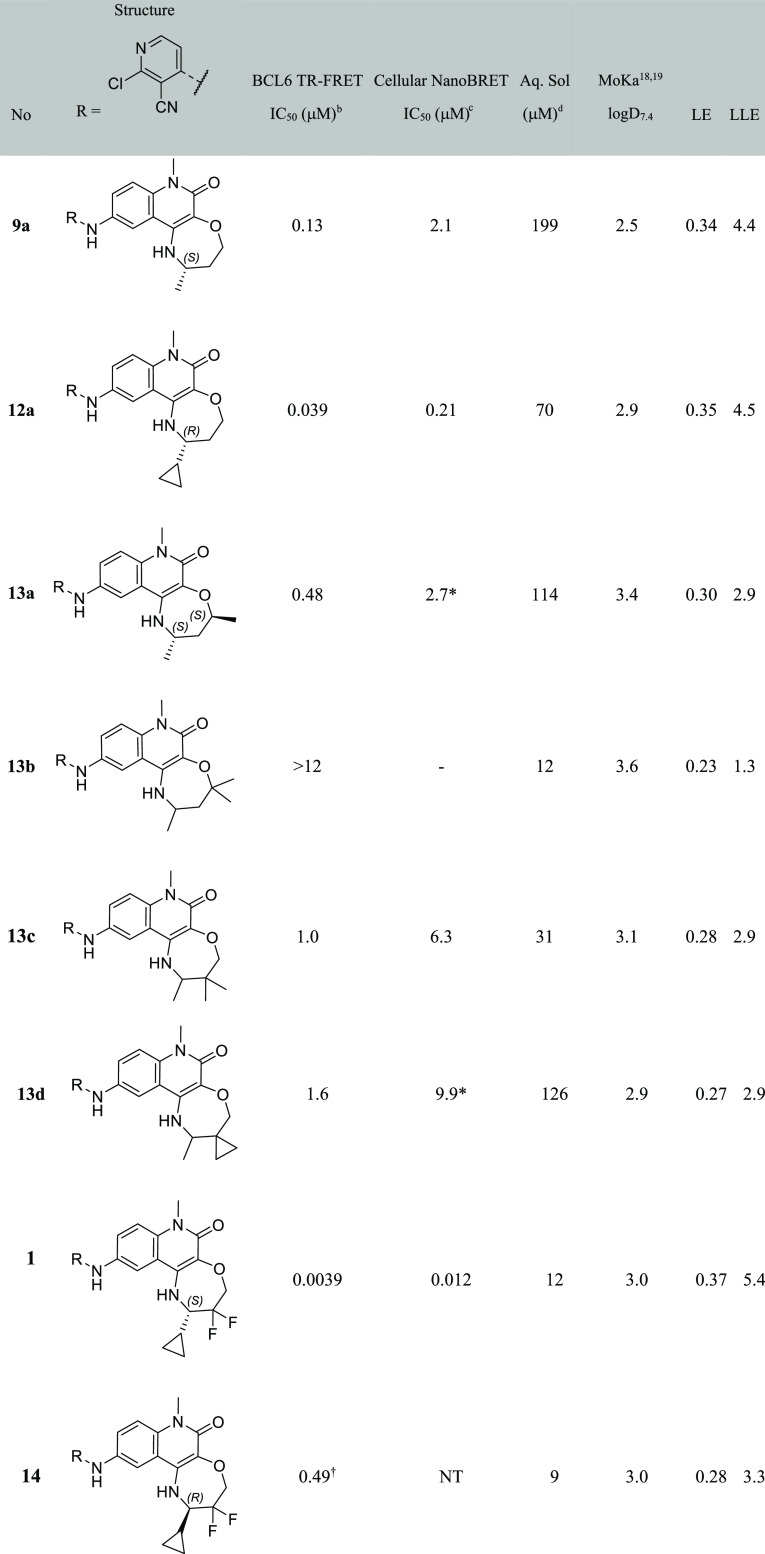
Biochemical and Cellular Data for Fused-7-ring Tricyclic Quinolinones with Additional Pocket-Facing Substitution^a^

a Individual replicates and errors are shown in Table S1. NT = not tested.

b Quoted values represent the geometric mean of at least three replicates. ^†^ indicates *n* = 2.

c Quoted values represent the geometric mean of two replicates. *n* = 1 where indicated by *.

d Kinetic solubility measured by NMR in HEPES buffer at pH 8, containing 4% DMSO.

Pleasingly, **1** showed potent cellular activity, with an IC_50_ value of 12 nM in the NanoBRET assay. This compound therefore shows not only a >300-fold improvement in biochemical potency over the ethylamine derivative **2** but also a 50-fold improvement in cellular potency (NanoBRET) over our previous best BCL6 inhibitor **3** (Figure 1). This was achieved through effective shape matching and space filling, without adding any new strong polar interactions (Figure 6). With this area of the pocket now filled, we had achieved our goal of discovering a potent, ligand efficient core. The optimization of this core into a potent degrader suitable for sustained depletion of BCL6 in vivo is described in a subsequent paper.

## Chemistry

**Scheme 1:**
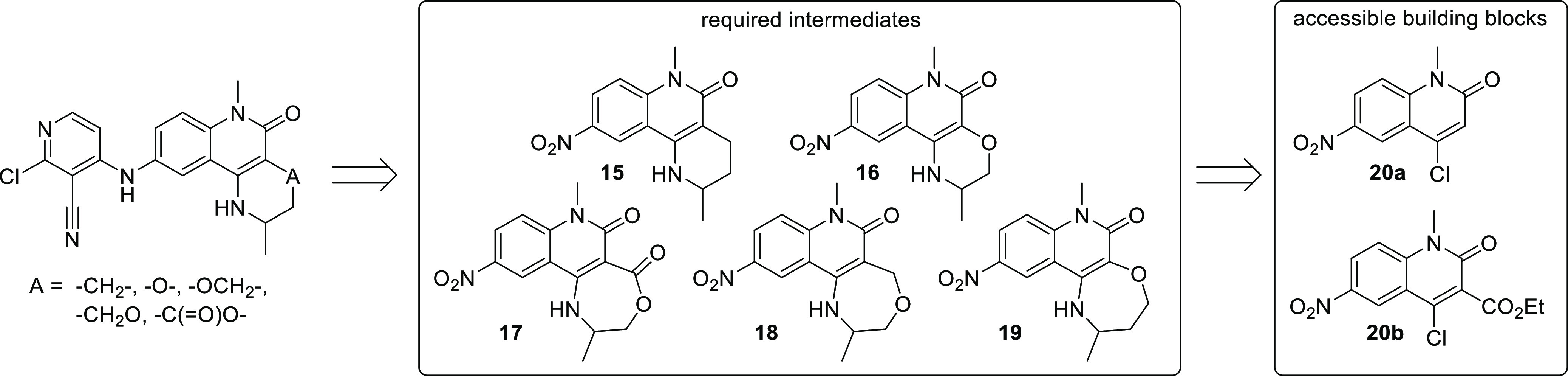
Synthetic Approach to Tricyclic Quinolinones

To enable our strategy of cyclization, we needed to rapidly develop and execute a range of varied synthetic routes to access largely unprecedented cores. We therefore targeted synthesis of novel nitro-substituted tricyclic cores **15–19**, which could be converted to final compounds by standard reduction and S_N_Ar reactions, and aimed to make these from building blocks we had previously prepared (Scheme 1).^16^

**Scheme 2:**
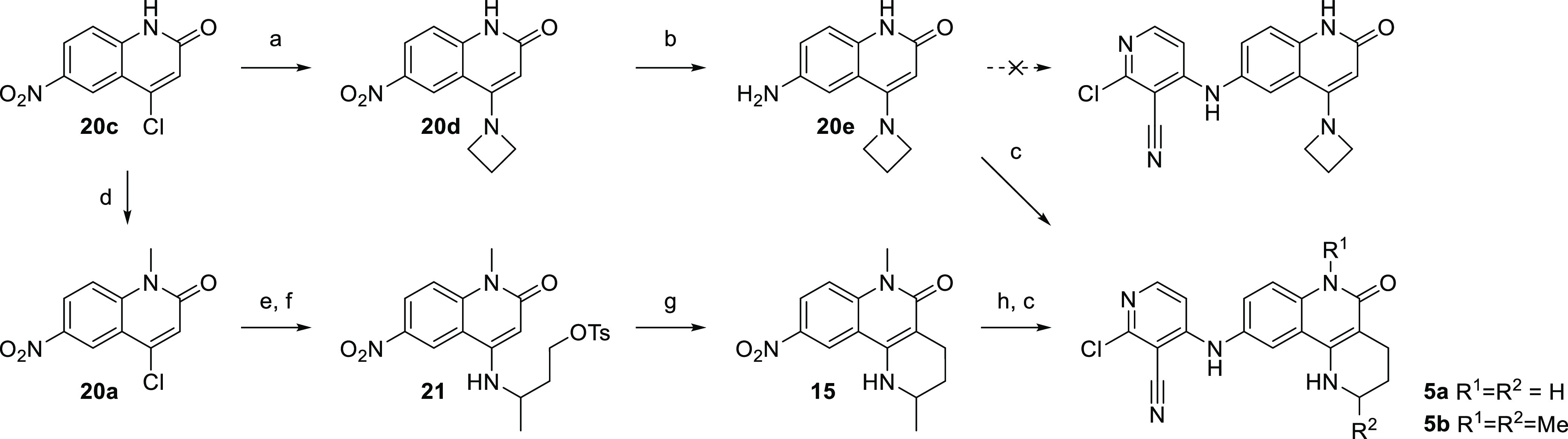
Synthesis of 2,3,4,6-tetrahydrobenzo[*h*][1,6]naphthyridin-5(1*H*)-ones (a) Azetidine, NMP, 160 °C μW, 1 h, 56%; (b) SnCl_2_, EtOH, 120 °C μW, 1 h, 97%; (c) 2,4-dichloropyridine-3-carbonitrile, TEA, NMP, 160 °C μW, 1 h, 8–51%; (d) NaH, DMF, 0 °C, 10 m, then MeI, rt, 1.25 h, 81%; (e) 3-aminobutan-1-ol, DIPEA, NMP, 160 °C, 20 h, 66%; (f) TsCl, py/DCM, 0 °C to rt, 20 h, 44%; (g) DIPEA, NMP, 160 °C μW, 1 h, 67%; (h) Pd/C, ammonium formate, EtOH/NMP, 60 °C, 0.5 h, 100%.

Previously reported synthetic routes to “piperidine-fused” quinolinones (2,3,4,6-tetrahydrobenzo[*h*][1,6]naphthyridin-5(1*H*)-ones, Table 1) were lengthy,^23,24^ and our desired substitution pattern was not precedented. However, while exploring azetidine substituents, a surprising solution presented itself: we observed a ring expansion of the azetidine of **20e** under the high-temperature S_N_Ar reaction conditions forming the desired fused compound **5a** (Scheme 2). We speculated that azetidine ring opening may be taking place, possibly by the chloride ion generated during the S_N_Ar. Intramolecular attack from the presumably nucleophilic quinolinone 3-position would then form the observed product. To investigate this hypothesis, we replaced the azetidine with alkyl tosylate **21** and were pleased to observe the desired cyclization to form target compound **5b**.

**Scheme 3:**
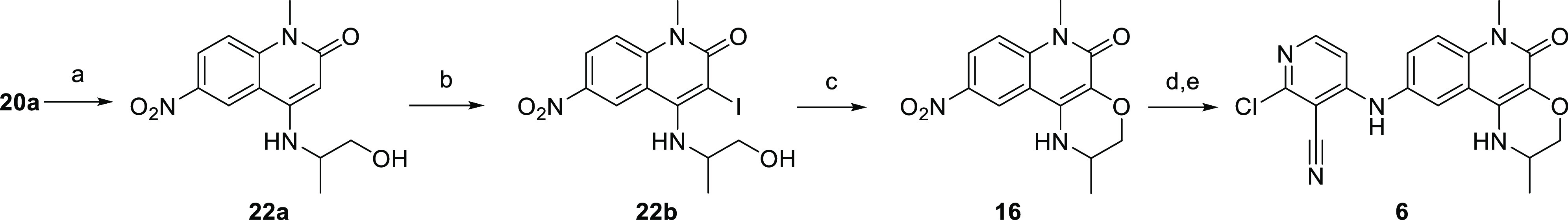
Synthesis of the 2,3-Dihydro-1*H*-[1,4]oxazino[2,3-*c*]quinolin-5(6*H*)-one Core (a) 2-Aminopropan-1-ol, DIPEA, NMP, 160 °C, 24 h, 87%; (b) iodine, methanol/water, 60 °C, 2 h, 38%; (c) 1,10-phen, CuI, cesium carbonate, NMP, 120 °C, 1 h, 41%; (d) Pd/C, ammonium formate, methanol, 80 °C, 1.5 h, 81–100%; (e) 2,4-dichloropyridine-3-carbonitrile, TEA, NMP, 160 °C, μW, 1.5 h, 60%.

The “fused morpholine” (2,3-dihydro-1*H*-[1,4]oxazino[2,3-*c*]quinolin-5(6*H*)-one, Scheme 3) core had not previously been reported in the literature. To enable rapid synthesis, we aimed to use existing 4-aminoquinolinone intermediates and therefore targeted making the key C–O bond via the Ullmann ether synthesis. Iodination of **22a** occurred selectively on the quinolinone 3-position, and copper-catalyzed C–O bond formation provided access to the desired target.

**Scheme 4:**
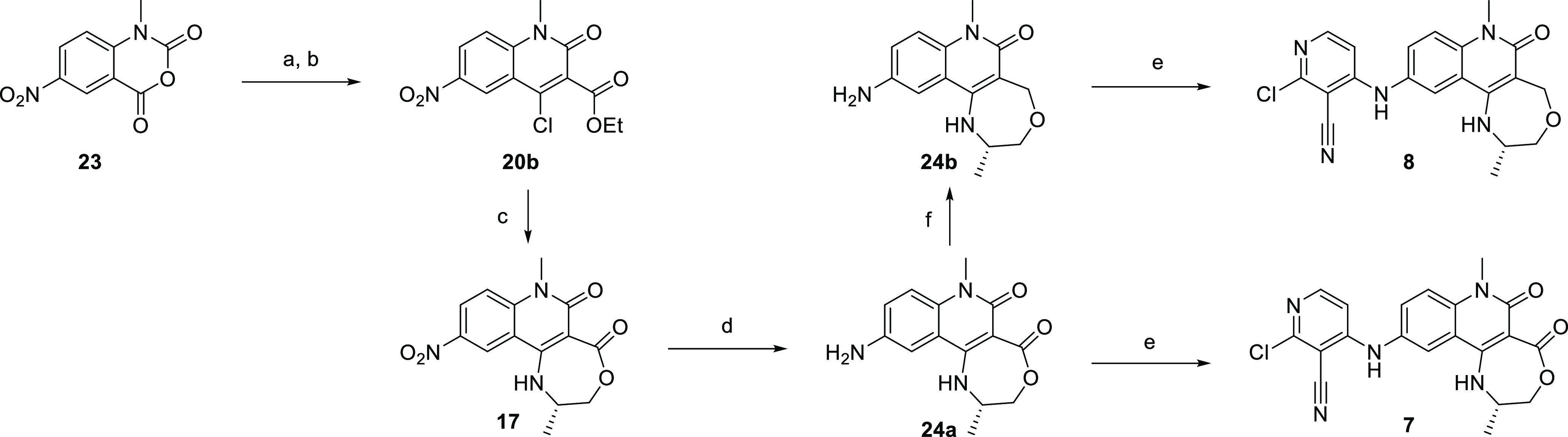
Synthesis of 2,3-Dihydro-[1,4]oxazepino[6,5-*c*]quinoline-5,6(1*H*,7*H*)-dione and 2,3,5,7-Tetrahydro-[1,4]oxazepino[6,5-*c*]quinolin-6(1*H*)-one Cores (a) Diethyl malonate, NaH, 0 °C–rt, 3 h, 94%; (b) POCl_3_, 80 °C, 2.5 h, 57%; (c) (*S*)-2-aminopropan-1-ol, DIPEA, NMP, 160 °C, 1 h, then LiCl 160 °C, 1 h, 37–70%; (d) H_2_, Pd/C, ethanol, rt, 16 h, 92%; (e) 2,4-dichloropyridine-3-carbonitrile, DIPEA, NMP, 160 °C, 1 h, 10–19%; (f) BF_3_·OEt_2_, THF, NaBH_4_, 0 °C, 2 h, 32%.

As the potency of **6** showed no improvement over the acyclic compounds, we moved on to explore seven-membered rings, aiming to more completely fill the available space in the pocket. Although our targeted cyclic lactone structures (2,3-dihydro-[1,4]oxazepino[6,5-*c*]quinoline-5,6(1*H*,7*H*)-diones, Scheme 4) were previously unreported, we had observed the formation of **17** as a byproduct during S_N_Ar reactions of ester **20b** with aminoalcohols.^16^ Following nitro reduction to **24a**, the resulting intermediate could be used to synthesise **7**. Access to a further target, **8**—containing the 2,3,5,7-tetrahydro-[1,4]oxazepino[6,5-*c*]quinolin-6(1*H*)-one core—required synthesis of ether **24b** (Scheme 4). This was prepared by reduction of the cyclic lactone group of **24a** using sodium borohydride in the presence of a Lewis acid.

**Scheme 5:**
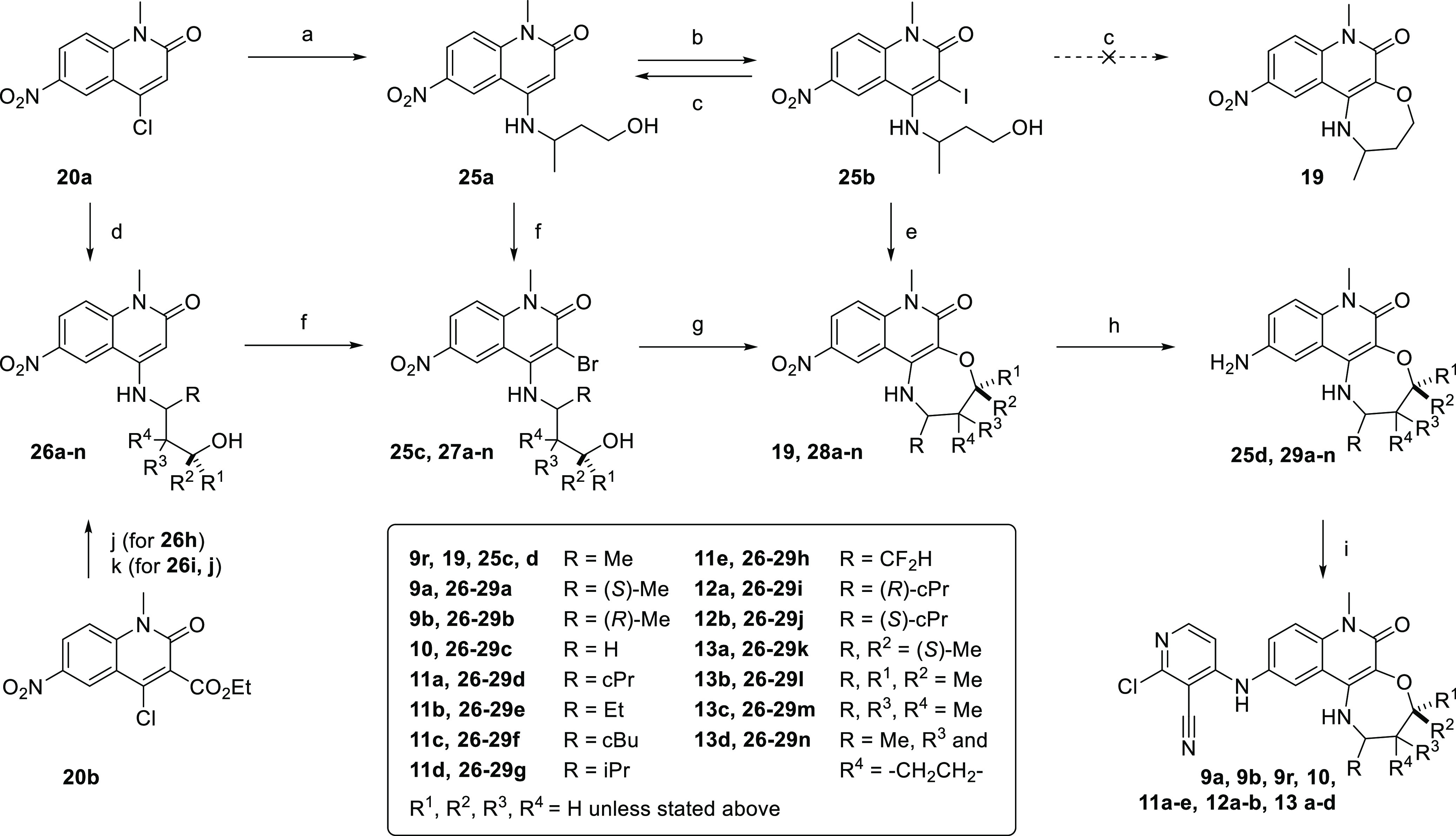
Synthetic Approaches to 1,2,3,4-Tetrahydro-[1,4]oxazepino[2,3-*c*]quinolin-6(7*H*)-ones (a) 3-Aminobutan-1-ol, DIPEA, NMP, 160 °C, 20 h, 66%; (b) iodine, methanol/water, 60 °C, 2 h, 38%; (c) 1,10-phen, CuI, cesium carbonate, NMP, 120 °C, 1 h, product not observed; (d) aminoalcohol, DIPEA or TEA, NMP, 160 °C, 1–4 d, 11–71%; (e) KO^*t*^Bu, THF/DMSO, μw, 100 °C, 10 min, 20%; (f) NBS, TFA, DCM, 0 °C–rt, 10–40 min, 40–77%; (g) KO^*t*^Bu, THF/DMSO, μw, 60 °C, 50–200 min, 7–51%; (h) ammonium formate, Pd/C, methanol, 80 °C, 20–30 min, 67–100%; (i) 2,4-dichloropyridine-3-carbonitrile, DIPEA or TEA, NMP, 160 °C, 60–90 min, 10–80%; (j) 3-amino-4,4-difluoro-butan-1-ol, DIPEA, NMP, 160 °C, 2 h then LiCl, 160 °C, 2 h, 70%; (k) 3-amino-3-cyclopropylpropan-1-ol, DIPEA, MeCN, 85 °C, 22 h then NaOH, THF/water, 85 °C, 6 h, 96%.

Our initial attempts to prepare the isomeric cyclic ethers (1,2,3,4-tetrahydro-[1,4]oxazepino[2,3-*c*]quinolin-6(7*H*)-ones, Scheme 5) from iodo-quinolinone **25b** by the Ullmann cyclization conditions used previously (Scheme 3) were unsuccessful, leading only to dehalogenation (Scheme 5, step c). With their conformation proposed to enable closer surface contact as described above, their synthesis remained a high priority. However, no syntheses of this core were previously reported, so we sought other methods for forming the desired C–O bond. We found an intriguing report suggesting that simple aryl ethers could be formed by microwave-heating alcohols with aryl halides in dimethyl sulfoxide (DMSO) in the presence of potassium *tert*-butoxide.^25^ Although the reported scope was both limited and quite different to our desired reaction, we applied these conditions to iodo-intermediate **25b**. We were delighted to observe the formation of desired intermediate **19**, albeit in a 1:1 ratio with dehalogenation product **25a**. We thought that the weak C–I bond may be favoring dehalogenation over the desired cyclisation and so instead prepared bromo-intermediate **25c** by an acid-catalyzed electrophilic bromination. Treatment of this intermediate with the cyclization conditions enabled improved conversion to products, and the resulting conditions were sufficiently effective to make final products **9a**, **9b**, **9r**, **10**, **11a–d**, **12a–b**, **13a–d**, and **14** (Scheme 5).

**Scheme 6:**
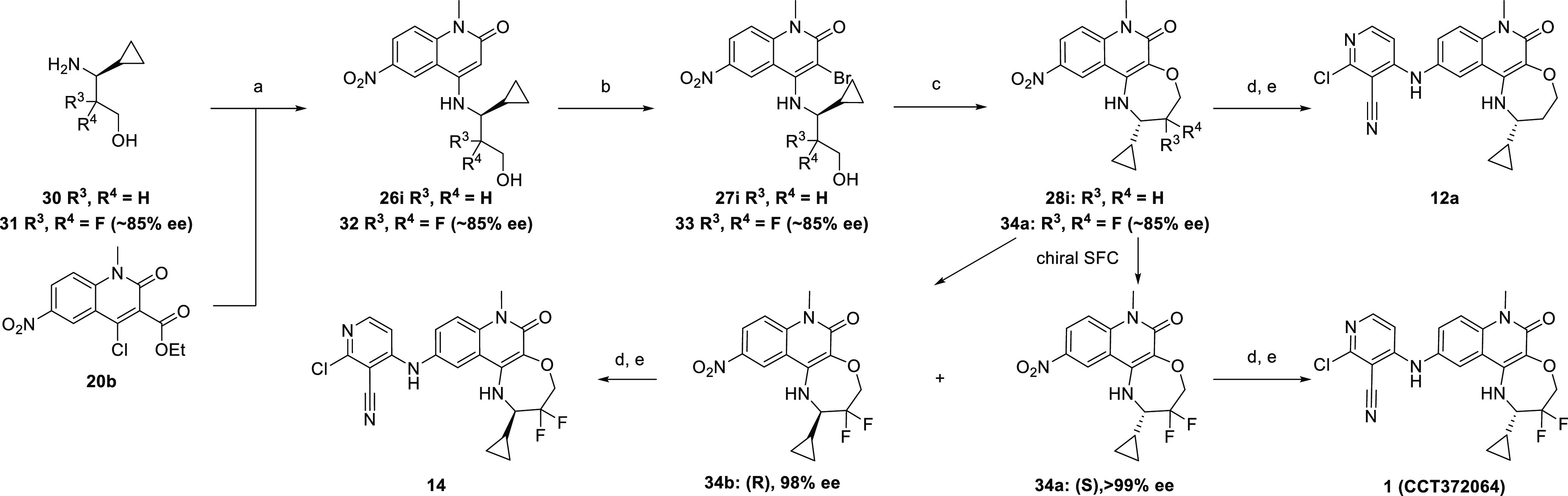
Synthesis of 2-Cyclopropyl-1,2,3,4-tetrahydro-[1,4]oxazepino[2,3-*c*]quinolin-6(7*H*)-ones (a) DIPEA (2.5 equiv), MeCN, 85 °C, 22 h, then 2 M NaOH (6 equiv), THF, 85 °C, 6 h, 96%; (b) NBS (1.5 equiv), TFA (5 equiv), DCM, 0 °C, 20 min, 58–79%; (c) LiOtBu (1 M in THF; 1.6 equiv), THF [0.1 M], 60 °C, 15 min, 99%; (d) H_2_, Pd/C, ethanol, 1–2 h, 99%; (e) 2,4-dichloropyridine-3-carbonitrile, TEA, NMP, 160 °C μW, 90 min, 51–53%.

We previously reported that replacing 4-chloroquinolinone **20a** with the more reactive chloro-ester **20b** allowed S_N_Ar reactions on the quinolinone core to proceed more readily.^16^ The activating ester group could then be removed by base-mediated hydrolysis/decarboxylation or treatment with lithium chloride. This increased reactivity enabled us to access electron-withdrawing substituents such as difluoromethyl (**11e**). We also applied this method to improve the yield for the synthesis of cyclopropyl compound **12a** following promising activity data. Replacing **20a** with ester **20b** allowed reduced temperature and higher yields, and desired intermediate **26i** was obtained in 96% yield over two steps, compared with 45% from the original route using **20a** (Scheme 6).

The microwave-assisted conditions for the key cyclization step to form the seven-membered rings gave modest and variable yields due to incomplete conversion, difficulty in separating the desired product from the unreacted starting material, and debromination to **26i** under the reaction conditions. Hence, we looked to optimize this reaction to make the synthesis of the core compatible with future scale-up. Optimization was carried out using the racemic methyl derivative **25c** (Table S4).

The standard reaction conditions under microwave irradiation afforded an approximately 3:1 ratio of cyclized product **19**/debromination product **25a**, reaching 91% conversion. Intermediate **19** was isolated in 41% yield. We wanted to switch to conventional heating to simplify scale-up, and this gave a similar ratio of ∼4:1 of **19/25a**. A range of bases and other additives were tried instead of the *tert*-butoxides, but product formation was not observed. Switching the counterion to sodium was tolerated, and the use of lithium *tert*-butoxide maintained a similar ratio of **19/25a** but gave improved conversion of the starting material.

The proposed mechanism for the published work is via a benzyne intermediate, and good yield was only obtained using DMSO as a solvent.^25^ However, this mechanism is not possible for our substrates, suggesting that a different reaction pathway must be occurring. We therefore undertook a short solvent screen. In our case, DMSO was not essential, and use of ether solvents or DCE minimized debromination, with tetrahydrofuran (THF) proving optimal. We could lower the equivalents of lithium *tert*-butoxide to 1.6 and increase the concentration of the reaction mixture to 0.1 M. Finally, we were able to significantly reduce the reaction time with complete conversion seen after only 15 min. An isolated yield of 81% of **19** was achieved after aqueous work-up with no further purification required.

Reoptimised conditions were used for the synthesis of the more potent cyclopropyl-containing tricyclic compounds (Scheme 6). Gratifyingly, under the new reaction conditions, cyclization occurred cleanly and a quantitative isolated yield was obtained for intermediate **28i** after aqueous work-up with no chromatography required. For the difluoro analogue, nitro intermediates **32, 33**, and **34a** were obtained in ∼85% ee due to the presence of the minor (*R*)-isomer as an impurity in the starting material (3*S*)-3-amino-3-cyclopropyl-2,2-difluoro-propan-1-ol hydrochloride, which we obtained commercially. These isomers were separated at the nitro stage by chiral supercritical fluid chromatography (SFC) and used to prepare lead compound **1** and its enantiomer **14**.

## Conclusions

**Figure 7:**
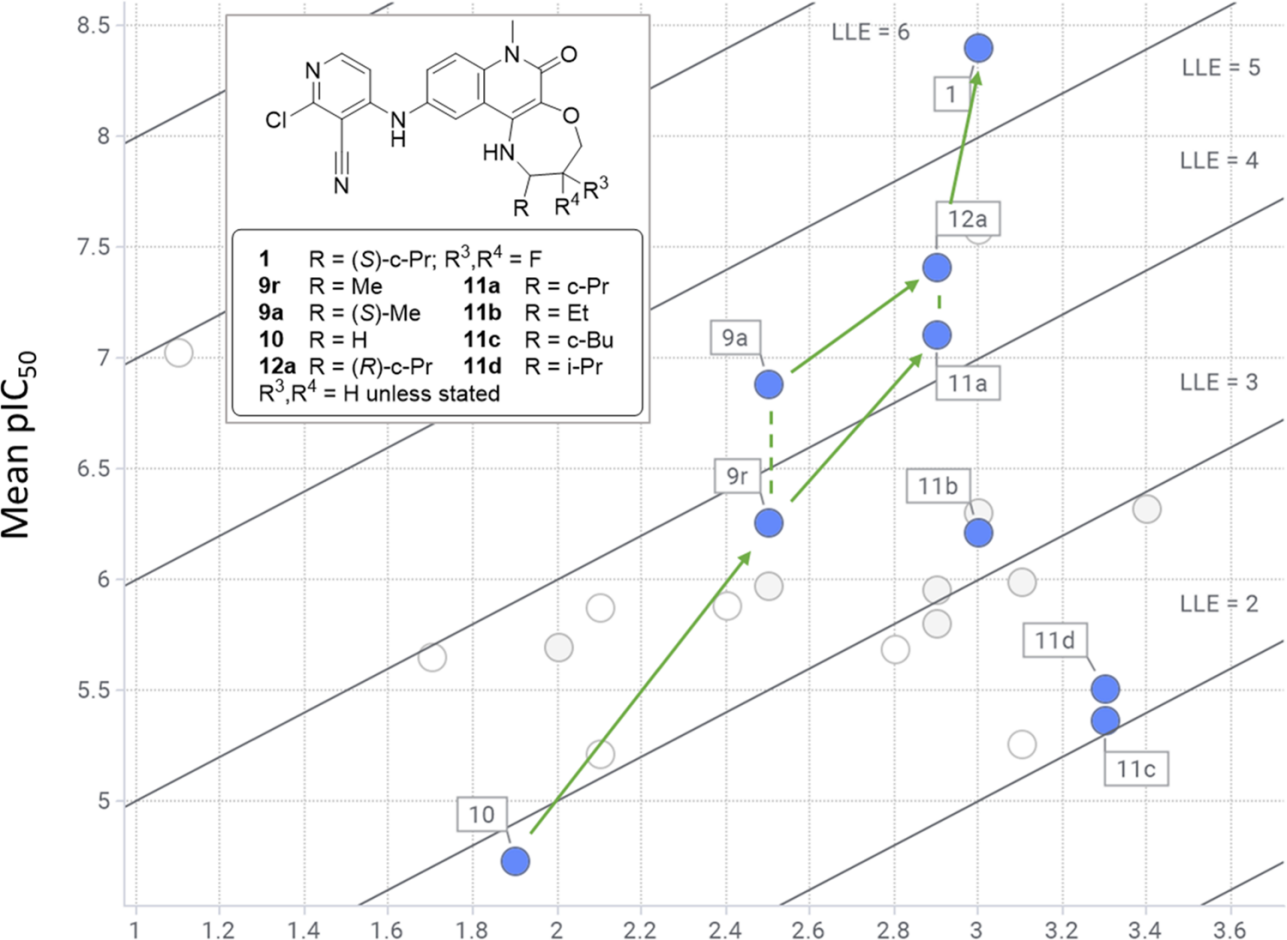
LLE plot of compounds from this study, reporting pIC50 vs calculated log *D*_7.4_ (Moka^18,19^), with iso-LLE lines. Hydrophobic substituents that improve shape complementarity generally exhibit increased LLE (**10**, **9a/r**, **11/12a**, **1**). In contrast, adding hydrophobic substituents that do not produce optimal surface contacts may maintain (**9r**, **11b**) or increase (**10**, **11c/d**) potency but show no improvement in LLE. Other compounds from the tricyclic ether series (**9b**, **11e**, **12b**, **13a**, **13c**, **13d**, **14**) are shown as gray circles, and those from other series presented in this paper (compounds **2–8**) are shown as white circles.

Obtaining sufficient binding affinity to inhibit protein–protein interactions can be challenging, and frequently, the resulting inhibitors are large and complex molecules, posing development challenges. To meet our objective of identifying chemical scaffolds with improved BCL6 binding affinity and a good ligand efficiency, we therefore focused on gaining as much binding affinity as possible from each area of the binding site. In this study, we particularly explored optimizing the shape complementarity in the BCL6 substrate binding region defined by residues Asp17, Val18, Cys53, and His14 (the HDCH site). Initial attempts to achieve this through introducing an additional substituent failed, likely because of the conformation effect on the adjacent substituent. We solved this issue by constraining both substituents into a ring and guided by X-ray crystallography designed a series of six- and seven-membered fused rings. A broad range of synthetic methods were used, taking advantage of available intermediates, to explore this diverse set of ring fusions. Cyclic lactone **7** provided the first validation of this approach, with high binding affinity, but poor permeability prevented further progression of this series. X-ray crystallography enabled us to prioritize possible single-point changes to the core structure—including moving the ether oxygen to form cyclic ether **9a**, predicted and later confirmed to have a 3D shape which more completely filled the corepressor binding pocket on the BTB domain of BCL6. The synthesis of this potent core required development of modified cyclization reaction conditions, and the broad scope of this reaction enabled us to explore a range of substituents, which we again prioritized based on X-ray structural information. We further optimized shape complementarity and achieved another breakthrough in potency by introducing a cyclopropyl group and then difluoro substituents to give our lead molecule **1**. The improvement in shape complementarity throughout this optimization can be seen in the comparison of crystal structures shown in Figure 6. The increase in LLE observed across this series (Figure 7) also demonstrates the potential utility of LLE plots in identifying compounds with improved shape complementarity. Overall, this work illustrates how optimizing shape complementarity leads to improved biological activity, while maintaining the overall lipophilicity under control and without introducing additional polar features.

Lead compound **1** is a potent and ligand-efficient inhibitor of BCL6 and shows excellent translation into the cellular NanoBRET assay (Table 4). Although our previous lead inhibitor **3** showed a >20-fold drop-off, **1** shows ∼3-fold from 3.9 nM (TR-FRET) to 12 nM (NanoBRET). We speculate that this discrepancy could result from differences in binding to the TR-FRET assay construct (BTB domain) and full length BCL6 used in the nanoBRET: this hypothesis has been previously proposed to explain mismatches between cell-free and cellular assays for a different series of BCL6 inhibitors.^26^ The improved cellular potency of **1** translates into increased antiproliferative activity in BCL6-high OCI-Ly1 and Karpas-422 cell lines, and selectivity over the BCL6-low OCI-Ly3 line is maintained (Table 4). With improved potency, a reduced molecular weight and polar surface area, and hence an increased ligand efficiency compared to **3** (Table 4), compound **1** (CCT372064) represents a new core structure for further elaboration toward the identification of candidate-quality BCL6 inhibitors; a subsequent publication will describe how this new core enabled the discovery of more potent, in vivo active degraders of BCL6.

**Table 4:** Comparison of Lead Compounds: Activity in Biochemical, Cellular, and Antiproliferative Effects; Permeability; Calculated Physicochemical Properties; and Ligand Efficiency Metrics

No	BCL6 TR-FRET IC50 (μM)	cellular NanoBRET IC_50_ (μM)	OCI-Ly1 GI_50_ (μM)	Karpas 422 GI_50_ (μM)	OCI-Ly3 GI_50_ (μM)	PAMPA (× 10^–6^ cm s^–1^) Pe (7.4)	MW	Moka^18,19^ log *D*_7.4_	TPSA Å^2^	LE	LLE
**2**	1.2	>30	NT	NT	NT	<0.1	340	2.4	94	0.35	3.5
**3** (CCT369347)	0.026	0.57	1.68	3.32	>10	35	486	3.0	109	0.30	4.6
**1** (CCT372064)	0.0039	0.012	0.17	0.19	3.4	5.5	458	3.0	92	0.36	5.3

## Experimental Section

### General Synthetic Information

All anhydrous solvents and reagents were obtained from commercial suppliers and used without further purification. Evaporation of solvents was carried out using a rotary evaporator under reduced pressure at a bath temperature of up to 60 °C. Flash column chromatography was carried out using a Biotage purification system using SNAP KP-Sil cartridges or on the reverse-phase mode using SNAP Ultra C18 cartridges. Semi-preparative separations were carried out using a 1200 series preparative high-performance liquid chromatography (HPLC) over a 15 min gradient elution. Microwave-assisted reactions were carried out using a Biotage initiator microwave system. Final compounds were purified to ≥95% purity. NMR data were collected on a Bruker AVANCE 500 spectrometer equipped with a 5 mm BBO/QNP probe or on a Bruker AVANCE Neo 600 spectrometer equipped with a 5 mm TCI Cryo-Probe. NMR data are presented in the form of chemical shift δ (multiplicity, coupling constants, integration) for major diagnostic protons, given in parts per million relative to tetramethylsilane, referenced to the internal deuterated solvent. High-resolution mass spectrometry (HRMS) was assessed using an Agilent 1200 series HPLC and a diode array detector coupled to a 6120 time of a flight mass spectrometer with a dual multimode APCI/ESI source or on a Waters Acquity UHPLC and a diode array detector coupled to a Waters G2 QToF mass spectrometer fitted with a multimode ESI/APCI source.

### Preparation of Compounds

Compounds **2**, **3**, **20a–c**, and **23** were prepared as previously reported.^16^

#### (*S*)-2-Chloro-4-((2-cyclopropyl-3,3-difluoro-7-methyl-6-oxo-1,2,3,4,6,7-hexahydro-[1,4]oxazepino[2,3-*c*]quinolin-10-yl)amino)nicotinonitrile (**1**, CCT372064)

##### Step 1: (*S*)-10-Amino-2-cyclopropyl-3,3-difluoro-7-methyl-1,2,3,4-tetrahydro-[1,4]oxazepino[2,3-*c*]quinolin-6(7*H*)-one

A mixture of **34a** (1.08 g, 3.09 mmol) and Pd/C (10 wt %, 108 mg) in ethanol (15 mL) was stirred at 60 °C under an atmosphere of H_2_ for 1 h. The reaction mixture was allowed to cool to rt and then filtered through Celite, and the solids were washed with ethanol (60 mL). The filtrate was concentrated under reduced pressure, affording the title compound (1.03 g, 100%) as an orange solid, which was used without purification in step 2. LCMS (2 min; ESI) RT 0.89 min; *m*/*z*: calcd for C_16_H_18_F_2_N_3_O_2_^+^ [M + H]^+^, 322.1362; found, 322.1370.

##### Step 2: 2-Chloro-4-[[(2*S*)-2-cyclopropyl-3,3-difluoro-7-methyl-6-oxo-2,4-dihydro-1*H*-[1,4]oxazepino[2,3-*c*]quinolin-10-yl]amino]pyridine-3-carbonitrile (**1**)

A mixture of the product of step 1 (12.5 mg, 0.039 mmol) with 2,4-dichloropyridine-3-carbonitrile (9.5 mg, 0.055 mmol) and triethylamine (TEA) (16 μL, 0.115 mmol) in *N*-methyl-2-pyrrolidinone (NMP) (0.6 mL) under argon was heated at 160 °C under microwave irradiation for 1.5 h. The reaction mixture was diluted with DMSO (0.8 mL) and purified using reverse-phase chromatography (10–85% methanol in water, 0.1% formic acid), affording **1** (9.4 mg, 53%) as an off-white solid. ^1^H NMR (600 MHz, CDCl_3_): δ 8.10 (d, *J* = 6.1 Hz, 1 H), 7.47–7.43 (m, 2 H), 7.38 (br s, 1 H), 6.98 (s, 1 H), 6.65 (d, *J* = 6.1 Hz, 1 H), 4.64 (dd, *J* = 16.3, 13.3 Hz, 1 H), 4.41 (ddd, *J* = 26.9, 13.3, 6.1 Hz, 1 H), 4.18–4.14 (m, 1 H), 3.74 (s, 3 H), 3.38–3.30 (m, 1 H), 1.38–1.31 (m, 1 H), 0.91–0.86 (m, 1 H), 0.77–0.68 (m, 2 H), 0.33–0.28 (m, 1 H); LCMS (4 min; ESI) RT 2.77 min *m*/*z*: calcd for C_22_H_19_ClF_2_N_5_O_2_^+^ [M + H]^+^, 458.1195; found, 458.1195.

#### 2-Chloro-4-((4-(ethylamino)-1,3-dimethyl-2-oxo-1,2-dihydroquinolin-6-yl)amino)nicotinonitrile (**4**)

##### Step 1: 4-Chloro-3-methyl-6-nitroquinolin-2(1*H*)-one

To a stirred solution of 2,4-dichloro-3-methyl-6-nitro-quinoline^27^ (480 mg, 1.87 mmol) in 1,4-dioxane (3.73 mL) was added conc. HCl (0.46 mL, 14.9 mmol) dropwise. The reaction mixture was refluxed for 18 h and then poured into ice water and stirred for 1 h. The resulting solid was filtered and dried under vacuum to afford the title compound (363 mg, 81%) as a white solid. The material was taken forward to the next step with no further purification. ^1^H NMR (500 MHz, DMSO-*d*_6_): δ 12.59 (s, 1 H), 8.57 (d, *J* = 2.5 Hz, 1 H), 8.37 (dd, *J* = 9.0, 2.5 Hz, 1 H), 7.47 (d, *J* = 9.0 Hz, 1 H), 2.24 (s, 3 H); LCMS (2 min; ToF) RT 1.44 min *m*/*z*: calcd for C_10_H_8_ClN_2_O_3_^+^ [M + H]^+^, 239.0218; found, 239.0200.

##### Step 2: 4-Chloro-1,3-dimethyl-6-nitroquinolin-2(1*H*)-one

To a solution of the product of step 1 (100 mg, 0.42 mmol) in dimethylformamide (DMF) (0.84 mL) at rt was added sodium hydride (60% in mineral oil, 33.5 mg, 0.84 mmol), followed by iodomethane (39 μL, 0.63 mmol). The mixture was stirred at rt for 1 h; then, water was added with care to the reaction mixture, forming an orange precipitate which was filtered under suction. The orange solid was washed several times with water. The residual solid was washed with DCM; then, the filtrate was separated, and the organic phase was concentrated. The resulting solid was combined with the solid isolated by filtration to give the title compound (101 mg, 95%). ^1^H NMR (500 MHz, DMSO-*d*_6_): δ 8.69 (d, *J* = 2.6 Hz, 1 H), 8.45 (dd, *J* = 9.3, 2.6 Hz, 1 H), 7.81 (d, *J* = 9.3 Hz, 1 H), 3.71 (s, 3 H), 2.31 (s, 3 H); LCMS (2 min; ToF) RT 1.46 min *m*/*z*: calcd for C_11_H_10_ClN_2_O_3_^+^ [M + H]^+^, 253.0374; found, 253.0350.

##### Step 3: 6-Amino-4-(ethylamino)-1,3-dimethylquinolin-2(1*H*)-one

To a suspension of the product of step 2 in NMP (1.42 mL) was added ethylamine (2.0 M in THF, 0.71 mL, 1.42 mmol), and the resulting mixture was heated to 200 °C for 12 h. After purification by HPLC (10–100% methanol in water, 0.1% formic acid), the title compound was isolated (7 mg, 19%, 91% purity by LCMS) as a pale-yellow solid, which was used without further purification in the next step. LCMS (2 min; ToF) RT 0.53 min *m*/*z*: calcd for C_13_H_18_N_3_O^+^ [M + H]^+^, 232.1444; found, 232.1422.

##### Step 4: 2-Chloro-4-((4-(ethylamino)-1,3-dimethyl-2-oxo-1,2-dihydroquinolin-6-yl)amino)nicotinonitrile (**4**)

A mixture of the product from step 3 (7 mg, 0.03 mmol), TEA (8.4 μL, 0.06 mmol), and 2,4-dichloropyridine-3-carbonitrile (6.3 mg, 0.036 mmol) in DMF (0.61 mL) was heated in the microwave for 1.5 h at 160 °C. The resulting mixture was purified by HPLC (40–100% methanol in water, 0.1% formic acid), affording **4** (4 mg, 36%) as a pale-yellow solid. ^1^H NMR (500 MHz, CDCl_3_): δ 8.05 (d, *J* = 6.1 Hz, 1 H), 7.66 (d, *J* = 2.4 Hz, 1 H), 7.43 (d, *J* = 8.9 Hz, 1 H), 7.38 (dd, *J* = 8.9, 2.4 Hz, 1 H), 6.97 (s, 1 H), 6.63 (d, *J* = 6.1 Hz, 1 H), 3.74 (s, 3 H), 3.31 (q, *J* = 7.1 Hz, 2 H), 2.23 (s, 3 H), 1.26 (t, *J* = 7.1 Hz, 3 H). LCMS (4 min; ToF) RT 2.68 min, *m*/*z*: calcd for C_19_H_19_ClN_5_O^+^ [M + H]^+^, 368.1273; found, 368.1275.

#### 2-Chloro-4-((5-oxo-1,2,3,4,5,6-hexahydrobenzo[*h*][1,6]naphthyridin-9-yl)amino)nicotinonitrile (**5a**)

##### Step 1: 4-(Azetidin-1-yl)-6-nitroquinolin-2(1*H*)-one (**20d**)

A suspension of **20c**(16) (43 mg, 0.19 mmol) and azetidine (77 μL, 1.15 mmol) in NMP (1.00 mL) was stirred at 160 °C under microwave irradiation for 1 h. Water was added, and the precipitate was collected by filtration. **20d** (26.5 mg, 56%, 91% purity by LCMS) was obtained as a yellow solid, which was used without further purification in step 2. ^1^H NMR (500 MHz, DMSO-*d*_6_): δ 11.51 (s, 1 H), 8.53 (d, *J* = 2.5 Hz, 1 H), 8.31 (dd, *J* = 9.1, 2.5 Hz, 1 H), 7.39 (d, *J* = 9.1 Hz, 1 H), 5.17 (s, 1 H), 4.35 (t, *J* = 7.5 Hz, 4 H), 2.43 (p, *J* = 7.5 Hz, 2 H); LCMS (2 min; ToF) RT 1.24 min, *m*/*z*: calcd for C_12_H_12_N_3_O_3_^+^ [M + H]^+^, 246.0884; found, 246.0886.

##### Step 2: 6-Amino-4-(azetidin-1-yl)quinolin-2(1*H*)-one (**20e**)

**20d** (20 mg, 0.082 mmol) and dichlorotin (62 mg, 0.33 mmol) were suspended in ethanol (4 mL). The mixture was heated in a microwave to 120 °C for 1 h. The solid was isolated by filtration and purified by SCX-2 to give **20e** (17 mg, 97%, 85% purity by LCMS). ^1^H NMR (500 MHz, DMSO-*d*_6_): δ 10.53 (s, 1 H), 6.97 (d, *J* = 8.6 Hz, 1 H), 6.89 (d, *J* = 2.3 Hz, 1 H), 6.76 (dd, *J* = 8.6, 2.4 Hz, 1 H), 5.01 (s, 1 H), 4.90 (s, 2 H), 4.16 (t, *J* = 7.4 Hz, 4 H), 2.38–2.28 (m, 2 H). LCMS (2 min; ToF) RT 0.17 min; *m*/*z*: calcd for C_12_H_14_N_3_O^+^ [M + H]^+^, 216.1131; found, 216.1145.

##### Step 3: 2-Chloro-4-((5-oxo-1,2,3,4,5,6-hexahydrobenzo[*h*][1,6]naphthyridin-9-yl)amino)nicotinonitrile (**5a**)

A suspension of **20e** (16 mg, 0.0743 mmol), 2,4-dichloropyridine-3-carbonitrile (19.3 mg, 0.11 mmol), and TEA (0.02 mL, 0.15 mmol) in NMP (1.5 mL) was stirred at 160 °C under microwave irradiation for 1 h. The resulting mixture was purified by HPLC to give **5a** (2 mg, 8%) as a yellow solid. ^1^H NMR (500 MHz, CD_3_OD): δ 7.98 (d, *J* = 6.3 Hz, 1 H), 7.75 (d, *J* = 1.9 Hz, 1 H), 7.51–7.30 (m, 2 H), 6.69 (d, *J* = 6.3 Hz, 1 H), 3.49–3.40 (m, 2 H), 2.64 (t, *J* = 6.1 Hz, 2 H), 1.95 (p, *J* = 6.1 Hz, 2 H). LCMS (4 min; ToF) Rt = 2.44 min, *m*/*z*: calcd for C_18_H_15_ClN_5_O [M + H]^+^, 352.0960; found, 352.0950.

#### 2-Chloro-4-((2,6-dimethyl-5-oxo-1,2,3,4,5,6-hexahydrobenzo[*h*][1,6]-naphthyridin-9-yl)amino)nicotinonitrile (**5b**)

##### Step 1: 4-((4-Hydroxybutan-2-yl)amino)-1-methyl-6-nitroquinolin-2(1*H*)-one

A mixture of **20a**(16) (250 mg, 1.05 mmol), 3-aminobutan-1-ol (280 mg, 3.14 mmol), and *N*,*N*-diisopropylethylamine (DIPEA) (0.36 mL, 2.10 mmol) in NMP (4.19 mL) was stirred at 160 °C for 20 h. The reaction mixture was allowed to cool to rt and then diluted with water and extracted with EtOAc. The organic extracts were combined, washed with water and brine, dried (Na_2_SO_4_), and concentrated under reduced pressure. Purification by flash chromatography (0 to 10% methanol in DCM) afforded the title compound (200 mg, 66%). ^1^H NMR (500 MHz, CDCl_3_): δ 8.48 (d, *J* = 2.5 Hz, 1 H), 8.36 (dd, *J* = 9.3, 2.5 Hz, 1 H), 7.38 (d, *J* = 9.3 Hz, 1 H), 6.05 (d, *J* = 6.6 Hz, 1 H), 5.80 (s, 1 H), 4.10–3.97 (m, 1 H), 3.96–3.82 (m, 2 H), 3.68 (s, 3 H), 2.08–1.95 (m, 2 H), 1.89 (dtd, *J* = 14.8, 6.3, 3.5 Hz, 1 H), 1.35 (d, *J* = 6.4 Hz, 3 H). LCMS (2 min; ToF) RT 1.20 min, *m*/*z*: calcd for C_14_H_18_N_3_O_4_^+^ [M + H]^+^, 292.1292; found, 292.1283.

##### Step 2: 3-((1-Methyl-6-nitro-2-oxo-1,2-dihydroquinolin-4-yl)amino)butyl 4-Methylbenzenesulfonate (**21**)

Tosyl chloride (196 mg, 1.03 mmol) was added to a stirred solution of the product from step 1 (100 mg, 0.34 mmol) and pyridine (3 mL, 37.2 mmol) in DCM (3.4 mL) at 0 °C. The reaction mixture was warmed to rt and stirred at this temperature for 20 h. The mixture was diluted with water and extracted with DCM. The organic extracts were combined, washed with 10% citric acid solution, dried (MgSO_4_), and concentrated under reduced pressure. Purification by flash chromatography (60 to 80% EtOAc in cyclohexane) afforded **21** (68 mg, 44%) as a yellow solid. ^1^H NMR (500 MHz, CDCl_3_): δ 8.43 (d, *J* = 2.4 Hz, 1 H), 8.39 (dd, *J* = 9.3, 2.4 Hz, 1 H), 7.77 (d, *J* = 8.2 Hz, 2 H), 7.41 (d, *J* = 9.3 Hz, 1 H), 7.28 (d, *J* = 8.2 Hz, 2 H), 5.74 (s, 1 H), 4.77 (d, *J* = 7.8 Hz, 1 H), 4.22 (t, *J* = 5.9 Hz, 2 H), 3.77 (app. hept, *J* = 6.7 Hz, 1 H), 3.69 (s, 3 H), 2.38 (s, 3 H), 2.09–2.02 (m, 2 H), 1.32 (d, *J* = 6.5 Hz, 3 H). LCMS (2 min; ToF) RT 1.42 min, *m*/*z*: calcd for C_21_H_24_N_3_O_6_S^+^ [M + H]^+^, 446.1380; found, 446.1368.

##### Step 3: 2,6-Dimethyl-9-nitro-2,3,4,6-tetrahydrobenzo[*h*][1,6]naphthyridin-5(1*H*)-one (**15**)

A mixture of **21** (from step 2, 34 mg, 0.076 mmol) and DIPEA (40 μL, 0.23 mmol) in NMP (0.76 mL) was heated at 160 °C under microwave irradiation for 1 h. The reaction mixture was allowed to cool to rt and then diluted with water and extracted with EtOAc. The organic extracts were combined, washed with water and brine, dried (MgSO_4_), and concentrated under reduced pressure. Purification by flash chromatography (0 to 10% methanol in DCM) afforded **15** (14 mg, 67%) as an orange solid. LCMS (2 min; ToF) RT 1.36 min, *m*/*z*: calcd for C_14_H_16_N_3_O_3_^+^ [M + H]^+^, 274.1186; found, 274.1161.

##### Step 4: 9-Amino-2,6-dimethyl-2,3,4,6-tetrahydrobenzo[*h*][1,6]naphthyridin-5(1*H*)-one

To a solution of **15** (from step 3, 14 mg, 0.0026 mmol) in ethanol (1 mL) and NMP (0.2 mL) was added Pd/C (10 wt %, 2.7 mg, 0.05 eq.), followed by ammonium formate (32 mg, 0.51 mmol). The resulting mixture was heated under an argon atmosphere at 60 °C in a sealed vial for 30 min and then filtered through Celite and purified using an SCX-2 (2 g) column, eluting with methanol (20 mL), followed by 2 N methanolic ammonia (20 mL). The ammonia fraction was concentrated under reduced pressure, affording the title compound (16 mg, 100%) as a yellow oil. LCMS (2 min; ToF) RT 0.62 min, *m*/*z*: calcd for C_14_H_18_N_3_O^+^ [M + H]^+^, 244.1444; found, 244.1443.

##### Step 5: 2-Chloro-4-((2,6-dimethyl-5-oxo-1,2,3,4,5,6-hexahydrobenzo[*h*][1,6]-naphthyridin-9-yl)amino)nicotinonitrile (**5b**)

To the product of step 4 (12 mg, 0.05 mmol) were added 2,4-dichloropyridine-3-carbonitrile (11 mg, 0.06 mmol), NMP (0.51 mL), and TEA (14 μL, 0.10 mmol). The resulting mixture was heated under an argon atmosphere in the microwave at 160 °C for 1 h. After cooling, the mixture was purified by HPLC (40–100% methanol in water, 0.1% formic acid), affording **5b** (10 mg, 51%) as an off-white solid. ^1^H NMR (500 MHz, CDCl_3_): δ 8.04 (d, *J* = 6.1 Hz, 1 H), 7.44–7.37 (m, 3 H), 6.92 (s, 1 H), 6.59 (d, *J* = 6.1 Hz, 1 H), 4.51 (s, 1 H), 3.70 (s, 3 H), 3.58–3.47 (m, 1 H), 2.86 (ddd, *J* = 17.6, 5.3, 3.9 Hz, 1 H), 2.60 (ddd, *J* = 17.6, 10.5, 5.9 Hz, 1 H), 2.09–2.00 (m, 1 H), 1.69–1.52 (m, 1 H), 1.34 (d, *J* = 6.4 Hz, 3H); LCMS (4 min; ESI) RT 2.60 min; *m*/*z*: calcd for C_20_H_19_ClN_5_O^+^ [M + H]^+^, 380.1278; found, 380.1280.

#### 2-Chloro-4-((2,6-dimethyl-5-oxo-2,3,5,6-tetrahydro-1*H*-[1,4]oxazino[2,3-*c*]quinolin-9-yl)amino)nicotinonitrile (**6**)

##### Step 1: 4-((1-Hydroxypropan-2-yl)amino)-1-methyl-6-nitroquinolin-2(1*H*)-one (**22a**)

A suspension of **20a**(16) (250 mg, 1.05 mmol), 2-aminopropan-1-ol (236 mg, 3.14 mmol), and DIPEA (0.36 mL, 2.10 mmol) in NMP (4.2 mL) was heated to 160 °C in a heating block for 24 h. The reaction mixture was allowed to cool to rt. Water (3 mL) was added to the reaction mixture, and after 5 min, a yellow precipitate formed. The mixture was added to water (20 mL). After 15 min, the precipitate was filtered, washed with water (100 mL), and dried, affording **22a** (252 mg, 87%) as a yellow solid. ^1^H NMR (500 MHz, DMSO-*d*_6_): δ 9.12 (d, *J* = 2.5 Hz, 1 H), 8.37 (dd, *J* = 9.4, 2.5 Hz, 1 H), 7.60 (d, *J* = 9.4 Hz, 1 H), 7.02 (d, *J* = 7.4 Hz, 1 H), 5.62 (s, 1 H), 4.82 (t, *J* = 5.8 Hz, 1 H), 3.66–3.52 (m, 5 H), 3.43–3.36 (m, 1 H), 1.22 (d, *J* = 6.4 Hz, 3 H); LCMS (2 min; ToF) RT 1.17 min; *m*/*z*: calcd for C_13_H_16_N_3_O_4_^+^ [M + H]^+^, 278.1135; found, 278.1120.

##### Step 2: 4-((1-Hydroxypropan-2-yl)amino)-3-iodo-1-methyl-6-nitroquinolin-2(1*H*)-one (**22b**)

To a mixture of **22a** (from step 1, 52 mg, 0.19 mmol) and iodine (145 mg, 0.57 mmol) under an argon atmosphere was added anhydrous methanol (1.2 mL), and the reaction mixture was heated at 60 °C under microwave irradiation for 30 min. Water (0.6 mL) was added, and the reaction mixture was heated at 60 °C under microwave irradiation for a further 90 min. The reaction mixture was allowed to cool to rt, diluted with methanol, and loaded onto silica. Purification by flash chromatography (0 to 15% methanol in DCM) afforded **22b** (29 mg, 38%) as a yellow solid. ^1^H NMR (500 MHz, CDCl_3_): δ 8.92 (d, *J* = 2.6 Hz, 1 H), 8.40 (dd, *J* = 9.3, 2.6 Hz, 1 H), 7.46 (d, *J* = 9.3 Hz, 1 H), 4.62 (d, *J* = 10.6 Hz, 1 H), 3.99–3.90 (m, 1 H), 3.86–3.76 (m, 5 H), 3.71 (dd, *J* = 11.2, 5.9 Hz, 1 H), 1.37 (d, *J* = 6.6 Hz, 3 H); LCMS (2 min; ToF) RT 1.31 min; *m*/*z*: calcd for C_13_H_15_IN_3_O_4_^+^ [M + H]^+^, 404.0102; found, 404.0108.

##### Step 3: 2,6-Dimethyl-9-nitro-2,3-dihydro-1*H*-[1,4]oxazino[2,3-*c*]quinolin-5(6*H*)-one (**16**)

To a mixture of **22b** (from step 2, 29 mg, 0.07 mmol), 1,10-phenanthroline (6 mg, 0.03 mmol), copper(I) iodide (3 mg, 0.02 mmol), and cesium carbonate (46 mg, 0.14 mmol) under an argon atmosphere was added anhydrous NMP (2.4 mL). The reaction mixture was heated at 120 °C under microwave irradiation for 1 h and then cooled to rt. Water (5 mL) was added, and the aqueous mixture was extracted with DCM (3 × 15 mL). The organic extracts were combined, dried (Na_2_SO_4_), and concentrated under reduced pressure. The crude product was purified by flash chromatography (0 to 15% methanol in DCM) and then further purified by SCX-2 and then by reverse-phase chromatography (10–100% methanol in water, 0.1% formic acid), affording **16** (8.2 mg, 41%) as a yellow solid. ^1^H NMR (500 MHz, CDCl_3_): δ 8.45 (d, *J* = 2.2 Hz, 1 H), 8.32 (dd, *J* = 9.3, 2.2 Hz, 1 H), 7.43 (d, *J* = 9.3 Hz, 1 H), 4.43 (br s, 1 H), 4.39 (dd, *J* = 10.5, 2.7 Hz, 1 H), 3.82 (dd, *J* = 10.5, 7.3 Hz, 1 H), 3.79 (s, 3 H), 3.76–3.72 (m, 1 H), 1.39 (d, *J* = 6.4 Hz, 3 H); LCMS (2 min; ToF) RT 1.21 min; *m*/*z*: calcd for C_13_H_14_N_3_O_4_^+^ [M + H]^+^, 276.0979; found, 276.0978.

##### Step 4: 9-Amino-2,6-dimethyl-2,3-dihydro-1*H*-[1,4]oxazino[2,3-*c*]quinolin-5(6*H*)-one

To a mixture of **16** (from step 3, 11 mg, 0.0392 mmol), Pd/C (10 wt %, 2 mg), and ammonium formate (13 mg, 0.20 mmol) under an argon atmosphere was added anhydrous methanol (0.4 mL), and the reaction mixture was stirred in a sealed vial at 80 °C for 90 min. The reaction mixture was allowed to cool to rt and filtered through Celite, and the solids were washed with methanol (20 mL). The filtrate was concentrated under reduced pressure, redissolved in methanol, and passed through an SCX-2 (2 g) column, washing with methanol (30 mL) and then eluting with 2 N methanolic ammonia (30 mL). The ammonia fraction was concentrated under reduced pressure, affording the title compound (7.8 mg, 81%) as an off-white solid, which was used without further purification. LCMS (2 min; ToF) RT 0.21 min; *m*/*z*: calcd for C_13_H_16_N_3_O_2_^+^ [M + H]^+^, 246.1237; found, 246.1253.

##### Step 5: 2-Chloro-4-((2,6-dimethyl-5-oxo-2,3,5,6-tetrahydro-1*H*-[1,4]oxazino[2,3-*c*]quinolin-9-yl)amino)nicotinonitrile (**6**)

To the product of step 4 (7.8 mg, 0.032 mmol) and 2,4-dichloropyridine-3-carbonitrile (9 mg, 0.052 mmol) under argon was added NMP (0.6 mL) and TEA (16 μL, 0.115 mmol). The resulting mixture was heated at 160 °C under microwave irradiation for 90 min. After cooling to rt, the mixture was diluted with DMSO and purified by reverse-phase chromatography (30–100% methanol in water, 0.1% formic acid) and then repurified by flash chromatography (0–15% methanol in DCM), affording **6** (7 mg, 60%) as an off-white solid. ^1^H NMR (600 MHz, CD_3_OD): δ 7.98 (d, *J* = 5.8 Hz, 1 H), 7.83 (s, 1 H), 7.62 (d, *J* = 8.8 Hz, 1 H), 7.47 (m, 1 H), 6.72 (d, *J* = 5.8 Hz, 1 H), 4.25 (d, *J* = 10.3 Hz, 1 H), 3.80–3.72 (m, 4 H), 3.70–3.64 (m, 1 H), 1.30 (d, *J* = 6.0 Hz, 3 H); LCMS (4 min; ToF) RT 2.51 min; *m*/*z*: calcd for C_19_H_17_ClN_5_O_2_^+^ [M + H]^+^, 382.1065; found, 382.1042.

#### (*S*)-2-Chloro-4-((2,7-dimethyl-5,6-dioxo-1,2,3,5,6,7-hexahydro-[1,4]oxazepino[6,5-*c*]quinolin-10-yl)amino)nicotinonitrile (**7**)

##### Step 1: (*S*)-2,7-Dimethyl-10-nitro-2,3-dihydro-[1,4]oxazepino[6,5-*c*]quinoline-5,6(1*H*,7*H*)-dione (**17**)

A suspension of **20b** (132 mg, 0.43 mmol), (*S*)-2-aminopropan-1-ol (64 mg, 0.85 mmol), and DIPEA (0.15 mL, 0.85 mmol) in NMP (1.5 mL) was heated at 160 °C under microwave irradiation for 1 h. The reaction mixture was allowed to cool to rt. Lithium chloride (108 mg, 2.55 mmol) was then added, and the mixture was heated at 160 °C under microwave irradiation for 1 h. The resulting mixture was purified by HPLC (40–100% methanol in water, 0.1% formic acid), affording **17** (48 mg, 37, 85% purity by LCMS) as a light-brown solid, which was used without further purification in subsequent steps. ^1^H NMR (500 MHz, DMF-*d*_7_): δ 9.20 (d, *J* = 2.4 Hz, 1 H), 8.48 (dd, *J* = 9.4, 2.4 Hz, 1 H), 8.20 (br s, 1 H), 7.72 (d, *J* = 9.4 Hz, 1 H), 4.72 (dd, *J* = 13.0, 1.2 Hz, 1 H), 4.49 (dd, *J* = 13.0, 5.1 Hz, 1 H), 4.20 (q, *J* = 6.4 Hz, 1 H), 3.66 (s, 3 H), 1.42 (d, *J* = 6.4 Hz, 3 H). LCMS (4 min; ToF) RT 1.05 min; *m*/*z*: calcd for C_14_H_14_N_3_O_5_^+^ [M + H]^+^, 304.0928; found, 304.0930.

##### Step 2: (*S*)-10-Amino-2,7-dimethyl-2,3-dihydro-[1,4]oxazepino[6,5-*c*]quinoline-5,6(1*H*,7*H*)-dione (**24a**)

A suspension of **17** (from step 1, 48 mg, 0.16 mmol) and Pd/C (10 wt %, 3.4 mg) in ethanol (4 mL) was stirred under an atmosphere of H_2_ for 16 h. The reaction mixture was filtered through Celite, and the solids washed with methanol. The filtrate was concentrated under reduced pressure, affording **24a** (40 mg, 92, 85% purity by LCMS) as a yellow oil, which was used without further purification. LCMS (2 min; ToF) RT 0.19 min; *m*/*z*: calcd for C_14_H_16_N_3_O_3_^+^ [M + H]^+^, 274.1186; found, 274.1179.

##### Step 3: (*S*)-2-Chloro-4-((2,7-dimethyl-5,6-dioxo-1,2,3,5,6,7-hexahydro-[1,4]oxazepino[6,5-*c*]quinolin-10-yl)amino)nicotinonitrile (**7**)

A suspension of DIPEA (13.4 μL, 0.077 mmol), 2,4-dichloropyridine-3-carbonitrile (6.2 mg, 0.036 mmol), and **24a** (from step 2, 7 mg, 0.026 mmol) in NMP (1.5 mL) was heated at 160 °C under microwave irradiation for 1 h. The crude reaction mixture was purified by HPLC (40–100% methanol in water, 0.1% formic acid), and the resulting light-brown solid was washed with diethyl ether and dried, affording **7** (1 mg, 10%). ^1^H NMR (500 MHz, DMF-*d*_7_): δ 8.84 (br s, 1H), 8.43 (d, *J* = 2.2 Hz, 1 H), 8.27 (d, *J* = 6.2 Hz, 1 H), 7.87 (dd, *J* = 9.0, 2.2 Hz, 1 H), 7.80 (s, 1 H), 7.78 (d, *J* = 9.0 Hz, 1 H), 7.03 (d, *J* = 6.2 Hz, 1 H), 4.81 (dd, *J* = 13.0, 1.5 Hz, 1 H), 4.63 (dd, *J* = 13.0, 5.5 Hz, 1 H), 4.24–4.30 (m, 1 H), 3.80 (s, 3 H), 1.52 (d, *J* = 6.6 Hz, 3 H); LCMS (4 min; ToF) RT 2.35 min; *m*/*z*: calcd for C_20_H_17_ClN_5_O_3_^+^ [M + H]^+^, 410.1014; found, 410.1008.

#### (*S*)-2-Chloro-4-((2,7-dimethyl-6-oxo-1,2,3,5,6,7-hexahydro-[1,4]oxazepino[6,5-*c*]quinolin-10-yl)amino)nicotinonitrile (**8**)

##### Step 1: (*S*)-10-Amino-2,7-dimethyl-2,3,5,7-tetrahydro-[1,4]oxazepino[6,5-*c*]quinolin-6(1*H*)-one (**24b**)

Boron trifluoride diethyl etherate (∼50% BF_3_; 0.1 mL, 0.41 mmol) was added to a stirred suspension of **24a** (from compound **7** synthesis, step 2, 12 mg, 0.042 mmol) in THF (4 mL) at 0 °C. The reaction mixture was stirred at 0 °C for 15 min, after which sodium borohydride (5 mg, 0.127 mmol) was added. The reaction mixture was stirred at 0 °C for a further 2 h. The reaction mixture was quenched with the addition of methanol and then concentrated under reduced pressure. Brine was added to the residue, and the aqueous mixture was extracted with EtOAc. The organic layer was dried (Na_2_SO_4_) and concentrated. Purification by flash chromatography (5% methanol in EtOAc) afforded **24b** (4 mg, 32%) as a yellow solid. LCMS (4 min; ToF) RT 0.69 min; *m*/*z*: calcd for C_14_H_18_N_3_O_2_^+^ [M + H]^+^, 260.1394; found, 260.1399.

##### Step 2: (*S*)-2-Chloro-4-((2,7-Dimethyl-6-oxo-1,2,3,5,6,7-hexahydro-[1,4]oxazepino[6,5-*c*]quinolin-10-yl)amino)nicotinonitrile (**8**)

A suspension of DIPEA (7.1 μL, 0.041 mmol), 2,4-dichloropyridine-3-carbonitrile (3.3 mg, 0.019 mmol), and **24b** (from step 1, 3.5 mg, 0.014 mmol) in NMP (1.5 mL) was stirred under microwave irradiation at 160 °C for 1 hr. The resulting mixture was purified first by HPLC and then by washing with ether to give **8** (1 mg, 19%, 0.0025 mmol) as a yellow solid. ^1^H NMR (600 MHz, CD_3_OD): δ 8.06 (d, *J* = 2.2 Hz, 1 H), 8.00 (d, *J* = 6.2 Hz, 1 H), 7.65 (d, *J* = 8.9 Hz, 1 H), 7.58 (dd, *J* = 8.9, 2.2 Hz, 1 H), 6.71 (d, *J* = 6.2 Hz, 1 H), 4.94 (d, *J* = 14.4 Hz, 1 H), 4.84 (d, *J* = 14.4 Hz, 1 H), 3.96 (m, 1 H), 3.93 (dd, *J* = 11.1, 3.0 Hz, 1 H), 3.72 (s, 3 H), 3.64 (dd, *J* = 11.1, 8.9 Hz, 1 H), 1.27 (d, *J* = 6.6 Hz, 3 H). LCMS (4 min; ToF) RT 2.53 min; *m*/*z*: calcd for C_20_H_19_ClN_5_O_2_^+^ [M + H]^+^, 396.1222; found, 396.1214.

#### (*S*)-2-Chloro-4-((2,7-dimethyl-6-oxo-1,2,3,4,6,7-hexahydro-[1,4]oxazepino-[2,3-*c*]quinolin-10-yl)amino)nicotinonitrile (**9a**)

##### Step 1: (*S*)-4-((4-Hydroxybutan-2-yl)amino)-1-methyl-6-nitroquinolin-2(1*H*)-one (**26a**)

To a mixture of **20a** (800 mg, 3.4 mmol) and (*S*)-3-aminobutan-1-ol (446 mg, 5.0 mmol) in a dry vial under argon was added anhydrous NMP (10 mL), followed by DIPEA (1.2 mL, 6.9 mmol). The reaction mixture was heated at 160 °C in a heating block for 20 h and then allowed to cool to rt and diluted with water (100 mL), and the aqueous mixture was extracted with EtOAc (100 mL). The organic extract was washed with water (2 × 25 mL). The aqueous washings were combined and further extracted with EtOAc (3 × 50 mL). The organic extracts were combined, dried (Na_2_SO_4_), and concentrated under reduced pressure. The crude reaction mixture was dry-loaded onto silica and purified by flash chromatography (0 to 10% methanol in DCM), affording **26a** (547 mg, 56%) as a yellow solid. ^1^H NMR (500 MHz, DMSO-*d*_6_): δ 9.11 (d, *J* = 2.5 Hz, 1 H), 8.37 (dd, *J* = 9.4, 2.5 Hz, 1 H), 7.60 (d, *J* = 9.4 Hz, 1 H), 7.11 (d, *J* = 7.9 Hz, 1 H), 5.59 (s, 1 H), 4.57 (t, *J* = 5.0 Hz, 1 H), 3.80–3.71 (m, 1 H), 3.55 (s, 3 H), 3.53–3.48 (m, 2 H), 1.94–1.87 (m, 1 H), 1.67–1.60 (m, 1 H), 1.23 (d, *J* = 6.4 Hz, 3 H). LCMS (2 min; ToF); RT 1.21 min; *m*/*z*: calcd for C_14_H_18_N_3_O_4_^+^ [M + H]^+^, 292.1292; found, 292.1274.

##### Step 2: (*S*)-3-Bromo-4-((4-hydroxybutan-2-yl)amino)-1-methyl-6-nitroquinolin-2(1*H*)-one (**27a**)

Trifluoroacetic acid (0.72 mL, 9.4 mmol) was added to a stirred mixture of *N*-bromosuccinimide (509 mg, 2.9 mmol) and **26a** (547 mg, 1.9 mmol) in anhydrous DCM (10 mL) at 0 °C under Ar. The reaction mixture was stirred at 0 °C for 10 min and at rt for 30 min and then diluted with ethyl acetate (100 mL) and washed with water (30 mL) and saturated aq. NaHCO_3_ (3 × 30 mL). The aqueous washings were combined and re-extracted with EtOAc (30 mL). The organic extracts were combined, dried (Na_2_SO_4_), and concentrated under reduced pressure and then dry-loaded onto silica and purified by flash chromatography (0 to 10% methanol in DCM), affording **27a** (532 mg, 77%) as a yellow solid. ^1^H NMR (500 MHz, DMSO-*d*_6_): δ 8.89 (d, *J* = 2.6 Hz, 1 H), 8.42 (dd, *J* = 9.4, 2.6 Hz, 1 H), 7.72 (d, *J* = 9.4 Hz, 1 H), 5.82 (d, *J* = 9.8 Hz, 1 H), 4.51 (t, *J* = 4.7 Hz, 1 H), 4.28–4.19 (m, 1 H), 3.69 (s, 3 H), 3.51–3.46 (m, 2 H), 1.90–1.82 (m, 1 H), 1.79–1.71 (m, 1 H), 1.29 (d, *J* = 6.5 Hz, 3 H). LCMS (2 min; ToF); RT 1.31 min; *m*/*z*: calcd for C_14_H_17_BrN_3_O_4_^+^ [M + H]^+^, 370.0397; found, 372.0390.

##### Step 3: (*S*)-2,7-Dimethyl-10-nitro-1,2,3,4-tetrahydro-[1,4]oxazepino[2,3-*c*]quinolin-6(7*H*)-one (**28a**)

To **27a** (111 mg, 0.30 mmol) under argon was added dry DMSO (4 mL) and then potassium *tert*-butoxide (1 M in THF; 0.54 mL, 0.54 mmol). The reaction mixture was heated at 60 °C under microwave irradiation for 50 min and then allowed to cool to rt. Water (10 mL) was added, followed by EtOAc (10 mL). The layers were separated, and the aqueous layer was further extracted with EtOAc (10 mL). The organic extracts were combined and concentrated under reduced pressure. The crude product was dissolved in DMSO (1.2 mL) and purified by reverse-phase chromatography (45–75% methanol in water, 0.1% formic acid), affording **28a** (36 mg, 41%) as a dark-yellow solid. ^1^H NMR (600 MHz, CDCl_3_): δ 8.93 (d, *J* = 2.1 Hz, 1 H), 8.33 (dd, *J* = 9.2, 2.1 Hz, 1 H), 7.40 (d, *J* = 9.2 Hz, 1 H), 4.49–4.38 (m, 2 H), 4.14–4.08 (m, 1 H), 4.01 (br s, 1 H), 3.76 (s, 3 H), 2.23–2.26 (m, 1 H), 1.91–1.84 (m, 1 H), 1.47 (d, *J* = 6.3 Hz, 3 H). LCMS (2 min; ToF); RT 1.26 min; *m*/*z*: calcd for C_14_H_16_N_3_O_4_^+^ [M + H]^+^, 290.1135; found, 290.1131.

##### Step 4: (*S*)-10-Amino-2,7-dimethyl-1,2,3,4-tetrahydro-[1,4]oxazepino[2,3-*c*]quinolin-6(7*H*)-one (**29a**)

A mixture of **28a** (35.8 mg, 0.124 mmol), Pd/C (10 wt %) (6.3 mg, 0.006 mmol), and ammonium formate (53.3 mg, 0.845 mmol) under argon in methanol (1.2 mL) was heated in a sealed vial to 80 °C for 20 min. Further, Pd/C (2.6 mg) and ammonium formate (32.5 mg) were added and heated to 80 °C for 10 min. The mixture was filtered through Celite, washing with methanol (40 mL). The filtrate was concentrated under reduced pressure, redissolved in methanol, and purified using an SCX-2 (2 g) column, washing with methanol (40 mL) and then eluting with 2 N methanolic ammonia (40 mL). The methanolic ammonia fraction was concentrated under reduced pressure, affording **29a** (21.5 mg, 67%, 0.083 mmol) as a dark-yellow solid, which was used without further purification. LCMS (2 min; ToF) RT 0.41 min *m*/*z*: calcd for C_14_H_18_N_3_O_2_^+^ [M + H]^+^, 260.1394; found, 260.1393.

##### Step 5: (*S*)-2-Chloro-4-((2,7-dimethyl-6-oxo-1,2,3,4,6,7-hexahydro-[1,4]oxazepino-[2,3-*c*]quinolin-10-yl)amino)nicotinonitrile (**9a**)

To a mixture of **29a** (8.7 mg, 0.034 mmol) and 2,4-dichloropyridine-3-carbonitrile (9.3 mg, 0.054 mmol) under argon was added NMP (0.6 mL), followed by TEA (14 μL, 0.10 mmol). The resulting mixture was heated at 160 °C under microwave irradiation for 90 min and then diluted with DMSO (0.8 mL) and purified using reverse-phase chromatography (10 to 100% methanol in water, 0.1% formic acid), affording **9a** (7.1 mg, 53%, 0.018 mmol) as an off-white solid. ^1^H NMR (600 MHz, CD_3_OD): δ 7.98 (d, *J* = 6.2 Hz, 1 H), 7.94 (d, *J* = 1.9 Hz, 1 H), 7.61 (d, *J* = 8.9 Hz, 1 H), 7.50 (dd, *J* = 8.9, 1.9 Hz, 1 H), 6.69 (d, *J* = 6.2 Hz, 1 H), 4.37–4.30 (m, 1 H), 4.28–4.22 (m, 1 H), 4.07–4.00 (m, 1 H), 3.73 (s, 3 H), 2.26–2.19 (m, 1 H),1.92–1.84 (m, 1 H), 1.38 (d, *J* = 6.6 Hz, 3 H); LCMS (4 min; ESI) RT 2.36 min; *m*/*z*: calcd for C_20_H_19_ClN_5_O_2_^+^ [M + H]^+^, 396.1222; found, 396.1208.

#### (*R*)-2-Chloro-4-((2,7-dimethyl-6-oxo-1,2,3,4,6,7-hexahydro-[1,4]oxazepino-[2,3-*c*]quinolin-10-yl)amino)nicotinonitrile (**9b**)

Prepared as described for its enantiomer, **9a**, starting from (*R*)-3-aminobutan-1-ol. ^1^H NMR as for **9a**. LCMS (4 min; ToF) RT = 2.58 min *m*/*z*: calcd for C_20_H_19_ClN_5_O_2_^+^ [M + H]^+^, 396.1222; found, 396.1213.

#### *rac*-2-Chloro-4-((2,7-dimethyl-6-oxo-1,2,3,4,6,7-hexahydro-[1,4]oxazepino-[2,3-*c*]quinolin-10-yl)amino)nicotinonitrile (**9r**)

Prepared as described for a single enantiomer, **9a**, starting from *rac*-3-aminobutan-1-ol. ^1^H NMR as for **9a**. LCMS (4 min; ToF) RT = 2.58 min *m*/*z*: calcd for C_20_H_19_ClN_5_O_2_^+^ [M + H]^+^, 396.1222; found, 396.1217.

#### 2-Chloro-4-((7-methyl-6-oxo-1,2,3,4,6,7-hexahydro-[1,4]oxazepino[2,3-*c*]quinolin-10-yl)amino)nicotinonitrile (**10**)

Prepared as described for **9a** starting from 3-aminopropanol. ^1^H NMR (600 MHz, DMSO-*d*_6_, 5% DCl): δ 8.04 (d, *J* = 6.2 Hz, 1 H), 7.96 (d, *J* = 1.9 Hz, 1 H), 7.56 (d, *J* = 8.9 Hz, 1 H), 7.46 (dd, *J* = 8.9, 1.9 Hz, 1 H), 6.71 (d, *J* = 6.2 Hz, 1 H), 4.17 (t, *J* = 6.3 Hz, 2 H), 3.60 (s, 3 H), 3.51 (br t, *J* = 5.4 Hz, 2 H), 2.04 (2H, m). LCMS (4 min; ToF) RT = 2.44 min *m*/*z*: calcd for C_19_H_17_ClN_5_O_2_^+^ [M + H]^+^, 382.1065; found, 382.1060.

#### 2-Chloro-4-((2-cyclopropyl-7-methyl-6-oxo-1,2,3,4,6,7-hexahydro-[1,4]oxazepino[2,3-*c*]quinolin-10-yl)amino)nicotinonitrile (**11a**)

Prepared by a five-step procedure as used for **9a**, starting from 3-amino-3-cyclopropylpropan-1-ol hydrochloride (107 mg, 0.70 mmol).

##### Step 1: 4-((1-Cyclopropyl-3-hydroxypropyl)amino)-1-methyl-6-nitroquinolin-2(1*H*)-one (**26d**)

Obtained as a yellow solid (47 mg, 44%). ^1^H NMR (500 MHz, DMSO-*d*_6_): δ 9.15 (d, *J* = 2.5 Hz, 1 H), 8.38 (dd, *J* = 9.4, 2.5 Hz, 1 H), 7.60 (d, *J* = 9.4 Hz, 1 H), 7.17 (d, *J* = 8.3 Hz, 1 H), 5.59 (s, 1 H), 4.51 (t, *J* = 5.0 Hz, 1 H), 3.59–3.53 (m, 4 H), 3.51–3.45 (m, 1 H), 3.27–3.22 (m, 1 H), 1.95–1.87 (m, 1 H), 1.84–1.77 (m, 1 H), 1.14–1.07 (m, 1 H), 0.53–0.47 (m, 1 H), 0.43–0.37 (m, 1 H), 0.28–0.23 (m, 2 H); LCMS (2 min; ToF) RT 1.31 min; *m*/*z*: calcd for C_16_H_20_N_3_O_4_^+^ [M + H]^+^, 318.1448; found, 318.1413.

##### Step 2: 3-Bromo-4-((1-cyclopropyl-3-hydroxypropyl)amino)-1-methyl-6-nitroquinolin-2(1*H*)-one (**27d**)

Obtained as a yellow solid (42 mg, 73%). ^1^H NMR (500 MHz, DMSO-*d*_6_): δ 8.94 (d, *J* = 2.6 Hz, 1 H), 8.41 (dd, *J* = 9.4, 2.6 Hz, 1 H), 7.73 (d, *J* = 9.4 Hz, 1 H), 5.66 (d, *J* = 10.3 Hz, 1 H), 4.52 (br s, 1 H), 3.70 (s, 3 H), 3.65–3.60 (m, 2 H), 3.56–3.48 (m, 1 H), 2.01–1.87 (m, 2 H), 1.15–1.06 (m, 1 H), 0.48–0.40 (m, 1 H), 0.35–0.28 (m, 1 H), 0.23–0.17 (m, 1 H), 0.08–0.02 (m, 1 H); LCMS (2 min; ToF) RT 1.38 min; *m*/*z*: calcd for C_16_H_19_BrN_3_O_4_^+^ [M + H]^+^, 396.0553; found, 396.0534.

##### Step 3: 2-Cyclopropyl-7-methyl-10-nitro-1,2,3,4-tetrahydro-[1,4]oxazepino[2,3-*c*]quinolin-6(7*H*)-one (**28d**)

Obtained as a dark-yellow solid (17 mg, 51%). ^1^H NMR (500 MHz, DMSO-*d*_6_): δ 9.03 (d, *J* = 2.6 Hz, 1 H), 8.31 (dd, *J* = 9.4, 2.6 Hz, 1 H), 7.60 (d, *J* = 9.4 Hz, 1 H), 6.62 (d, *J* = 4.4 Hz, 1 H), 4.26–4.18 (m, 2 H), 3.60 (s, 3 H), 2.94–2.87 (m, 1 H), 2.25–2.18 (m, 1 H), 2.05–1.99 (m, 1 H), 1.29–1.23 (m, 1 H), 0.57–0.49 (m, 2 H), 0.41–0.36 (m, 1 H), 0.30–0.24 (m, 1 H); LCMS (2 min, ToF) RT 1.37 min; *m*/*z*: calcd for C_16_H_18_N_3_O_4_^+^ [M + H]^+^, 316.1292; found, 316.1291.

##### Step 4: 10-Amino-2-cyclopropyl-7-methyl-1,2,3,4-tetrahydro-[1,4]oxazepino[2,3-*c*]quinolin-6(7*H*)-one (**29d**)

Obtained as a yellow solid (13 mg, 85%), which was used without further purification. LCMS (2 min; ToF) RT 0.91 min; *m*/*z*: calcd for C_16_H_20_N_3_O_2_^+^ [M + H]^+^, 286.1550; found, 286.1551.

##### Step 5: 2-Chloro-4-((2-cyclopropyl-7-methyl-6-oxo-1,2,3,4,6,7-hexahydro-[1,4]oxazepino[2,3-*c*]quinolin-10-yl)amino)nicotinonitrile (**11a**)

Obtained as an off-white solid (7 mg, 69%). ^1^H NMR (600 MHz, CD_3_OD): δ 7.99 (d, *J* = 6.2 Hz, 1 H), 7.95 (d, *J* = 2.1 Hz, 1 H), 7.61 (d, *J* = 8.9 Hz, 1 H), 7.51 (dd, *J* = 8.9, 2.1 Hz, 1 H), 6.74 (d, *J* = 6.2 Hz, 1 H), 4.41–4.35 (m, 1 H), 4.24–4.19 (m, 1 H), 3.72 (s, 3 H), 2.92 (td, *J* = 9.4, 3.7 Hz, 1 H), 2.37–2.29 (m, 1 H), 2.12–2.06 (m, 1 H), 1.24–1.16 (m, 1 H), 0.66–0.57 (m, 2 H), 0.39–0.34 (m, 1 H), 0.32–0.28 (m, 1 H); LCMS (4 min; ToF) RT 2.73 min; *m*/*z*: calcd for C_22_H_21_ClN_5_O_2_^+^ [M + H]^+^, 422.1378; found, 422.1369.

#### 2-Chloro-4-((2-ethyl-7-methyl-6-oxo-1,2,3,4,6,7-hexahydro-[1,4]oxazepino[2,3-*c*]quinolin-10-yl)amino)nicotinonitrile (**11b**)

##### Step 1: 4-((1-Hydroxypentan-3-yl)amino)-1-methyl-6-nitroquinolin-2(1*H*)-one (**26e**)

Prepared from **20a** (170 mg, 0.71 mmol) and 3-aminopentan-1-ol (110 mg, 1.07 mmol) using the procedure described for **26a** (**9a** step 1). **26e** (94 mg, 43%) was obtained as a yellow solid. ^1^H NMR (500 MHz, DMSO-*d*_6_): δ 9.17 (d, *J* = 2.5 Hz, 1 H), 8.38 (dd, *J* = 9.3, 2.5 Hz, 1 H), 7.61 (d, *J* = 9.3 Hz, 1 H), 7.04 (d, *J* = 8.3 Hz, 1 H), 5.62 (s, 1 H), 4.54 (t, *J* = 4.9 Hz, 1 H), 3.61 (q, *J* = 6.9 Hz, 1 H), 3.56 (s, 3 H), 3.52–3.38 (m, 2 H), 1.83 (td, *J* = 13.7, 5.9 Hz, 1 H), 1.76–1.67 (m, 1 H), 1.69–1.59 (m, 2 H), 0.91 (t, *J* = 7.4 Hz, 3 H). LCMS (2 min; ToF) RT 1.29 min; *m*/*z*: calcd for C_15_H_20_N_3_O_4_^+^ [M + H]^+^, 306.1448; found, 306.1447.

##### Step 2: 3-Bromo-4-((1-hydroxypentan-3-yl)amino)-1-methyl-6-nitroquinolin-2(1*H*)-one (**27e**)

Prepared by bromination of **26e** (94 mg, 0.31 mmol) using the procedure described for **27b** (**9a** step 2). **27e** (47 mg, 40%) was obtained as a yellow solid. ^1^H NMR (500 MHz, DMSO- *d*_*6*_): δ 8.94 (d, *J* = 2.6 Hz, 1 H), 8.41 (dd, *J* = 9.3, 2.6 Hz, 1 H), 7.72 (d, *J* = 9.3 Hz, 1 H), 5.72 (d, *J* = 10.1 Hz, 1 H), 4.50 (s, 1 H), 4.14 (dt, *J* = 10.1, 6.2 Hz, 1 H), 3.69 (s, 3 H), 3.50 (d, *J* = 6.2 Hz, 2 H), 1.80 (ddd, *J* = 8.9, 7.5, 4.6 Hz, 2 H), 1.78–1.59 (m, 2 H), 0.94 (t, *J* = 7.4 Hz, 3 H); LCMS (2 min; ToF) RT 1.40 min; *m*/*z*: calcd for C_15_H_19_BrN_3_O_4_^+^ [M + H]^+^, 384.0553; found, 384.0529.

##### Step 3: 2-Ethyl-7-methyl-10-nitro-1,2,3,4-tetrahydro-[1,4]oxazepino[2,3-*c*]quinolin-6(7*H*)-one (**28e**)

Prepared by cyclization of **27e** (19 mg, 0.05 mmol) using the procedure described for **28a** (**9a** step 3). The reaction mixture was purified by HPLC (40–100% methanol in water, 0.1% formic acid), affording **28e** as a brown oil (2.5 mg, 17%). ^1^H NMR (500 MHz, CD_3_OD): δ 8.94 (d, *J* = 2.5 Hz, 1 H), 8.36 (dd, *J* = 9.3, 2.5 Hz, 1 H), 7.65 (d, *J* = 9.3 Hz, 1 H), 4.43–4.30 (m, 2 H), 3.86–3.77 (m, 1 H), 3.75 (s, 3 H), 2.30 (dddd, *J* = 13.9, 8.4, 6.9, 4.1 Hz, 1 H), 1.97–1.84 (m, 2 H), 1.81–1.69 (m, 1 H), 1.07 (t, *J* = 7.4 Hz, 3 H); LCMS (2 min; ToF) RT 1.35 min; *m*/*z*: calcd for C_15_H_18_N_3_O_4_^+^ [M + H]^+^, 304.1292; found, 304.1285.

##### Step 4: 10-Amino-2-ethyl-7-methyl-1,2,3,4-tetrahydro-[1,4]oxazepino[2,3-*c*]quinolin-6(7*H*)-one (**29e**)

Prepared by transfer hydrogenation of **28e** (2.5 mg, 0.008 mmol) using the procedure described for **29a** (**9a** step 4), affording **29e** (2 mg, 89%) as a yellow oil, used without purification in the next step. LCMS (2 min; ToF) RT 0.84 min; *m*/*z*: calcd for C_15_H_20_N_3_O_2_^+^ [M + H]^+^, 274.1550; found, 274.1542.

##### Step 5: 2-Chloro-4-((2-ethyl-7-methyl-6-oxo-1,2,3,4,6,7-hexahydro-[1,4]oxazepino[2,3-*c*]quinolin-10-yl)amino)nicotinonitrile (**11b**)

Prepared from **29e** (2 mg) using the procedure described for **7**. Additional purification by passing the product through an SCX-2 column afforded **11b** as an off-white solid (1 mg, 33%). ^1^H NMR (600 MHz, CD_3_OD): δ 7.99 (d, *J* = 6.2 Hz, 1 H), 7.96 (d, *J* = 2.3 Hz, 1 H), 7.63 (d, *J* = 9.0 Hz, 1 H), 7.52 (dd, *J* = 9.0, 2.3 Hz, 1 H), 6.73 (d, *J* = 6.2 Hz, 1 H), 4.46–4.26 (m, 2 H), 3.84–3.77 (m, 1 H), 3.75 (s, 3 H), 2.34–2.25 (m, 1 H), 1.93–1.85 (m, 1 H), 1.85–1.80 (m, 1 H), 1.75–1.65 (m, 1 H), 1.04 (t, *J* = 7.4 Hz, 3 H); LCMS (4 min; ToF) RT 2.69 min; *m*/*z*: calcd for C_21_H_21_ClN_5_O_2_^+^ [M + H]^+^, 410.1378; found, 410.1372.

#### 2-Chloro-4-((2-cyclobutyl-7-methyl-6-oxo-1,2,3,4,6,7-hexahydro-[1,4]oxazepino[2,3-*c*]quinolin-10-yl)amino)nicotinonitrile (**11c**)

##### Step 1: 4-((1-Cyclobutyl-3-hydroxypropyl)amino)-1-methyl-6-nitroquinolin-2(1*H*)-one (**26f**)

Prepared from **20a** (181 mg, 0.76 mmol) and 3-amino-3-cyclobutylpropan-1-ol hydrochloride (251 mg, 1.52 mmol) using the procedure described for **26a** with a reaction time of 4 days to give **26f** as a yellow solid (158 mg, 63%). 1H NMR (500 MHz, DMSO-*d*_6_): δ 9.16 (d, *J* = 2.5 Hz, 1 H), 8.37 (dd, *J* = 9.4, 2.5 Hz, 1 H), 7.59 (d, *J* = 9.4 Hz, 1 H), 6.95 (d, *J* = 8.8 Hz, 1 H), 5.70 (s, 1 H), 4.47 (t, *J* = 4.9 Hz, 1 H), 3.76–3.67 (m, 1 H), 3.54 (s, 3 H), 3.47–3.42 (m, 1 H), 3.42–3.35 (m, 1 H), 2.65–2.55 (m, 1 H), 2.00–1.88 (m, 2 H), 1.82–1.70 (m, 4 H), 1.69–1.64 (m, 2 H); LCMS (2 min; ESI); RT 1.28 min; *m*/*z*: calcd for C_17_H_22_N_3_O_4_^+^ [M + H]^+^, 332.1605; found, 332.1614.

##### Step 2: 3-Bromo-4-((1-cyclobutyl-3-hydroxypropyl)amino)-1-methyl-6-nitroquinolin-2(1*H*)-one (**27f**)

Trifluoroacetic acid (0.19 mL, 2.48 mmol) was added to a stirred mixture of *N*-bromosuccinimide (128 mg, 0.72 mmol) and **26f** (158 mg, 0.48 mmol) in dichloromethane (3.2 mL) at 0 °C under Ar. The reaction mixture was stirred at 0 °C for 15 min and then diluted with EtOAc (20 mL) and washed with saturated aq. NaHCO_3_ (2 × 15 mL). The aqueous washings were re-extracted with EtOAc (20 mL). The organic extracts were combined, dried (Na_2_SO_4_), and concentrated under reduced pressure and then purified by flash chromatography (0 to 10% methanol in DCM), affording **27f** (132 mg, 68%) as a yellow solid. ^1^H NMR (500 MHz, DMSO-*d*_6_): δ 8.96 (d, *J* = 2.6 Hz, 1 H), 8.42 (dd, *J* = 9.4, 2.6 Hz, 1 H), 7.73 (d, *J* = 9.4 Hz, 1 H), 5.44 (d, *J* = 10.5 Hz, 1 H), 4.52 (br s, 1 H), 4.17–4.08 (m, 1 H), 3.69 (s, 3 H), 3.58 (t, *J* = 6.7 Hz, 2 H), 2.62–2.57 (m, 1 H), 1.96–1.89 (m, 1 H), 1.78–1.69 (m, 5 H), 1.64–1.56 (m, 2 H); LCMS (2 min; ESI); RT 1.37 min; *m*/*z*: calcd for C_17_H_21_BrN_3_O_4_^+^ [M + H]^+^, 410.0710; found, 410.0709.

##### Step 3: 2-Cyclobutyl-7-methyl-10-nitro-1,2,3,4-tetrahydro-[1,4]oxazepino[2,3-*c*]quinolin-6(7*H*)-one (**28f**)

Prepared by cyclization of **27f** (132 mg, 0.32 mmol) according to the procedure used for **28a** to afford the title compound as a yellow solid (36 mg, 33%). ^1^H NMR (500 MHz, DMSO-*d*_6_): δ 8.97 (d, *J* = 2.5 Hz, 1 H), 8.29 (dd, *J* = 9.3, 2.5 Hz, 1 H), 7.60 (d, *J* = 9.3 Hz, 1 H), 6.32 (d, *J* = 4.9 Hz, 1 H), 4.23–4.18 (m, 1 H), 4.18–4.12 (m, 1 H), 3.68–3.62 (m, 1 H), 3.60 (s, 3 H), 2.71–2.64 (m, 1 H), 2.15–2.08 (m, 1 H), 2.07–2.00 (m, 2 H), 1.84–1.70 (m, 5 H); LCMS (2 min; ESI); RT 1.39 min; *m*/*z*: calcd for C_17_H_20_N_3_O_4_^+^ [M + H]^+^, 330.1448; found, 330.1455.

##### Step 4: 10-Amino-2-cyclobutyl-7-methyl-1,2,3,4-tetrahydro-[1,4]oxazepino[2,3-*c*]quinolin-6(7*H*)-one (**29f**)

Prepared by transfer hydrogenation of **28f** (36 mg, 0.11 mmol) according to the procedure used for **29a**. The reaction time was 20 min, affording **29f** (32 mg, 99%) as a dark-orange solid. LCMS (2 min; ESI) RT 0.95 min; *m*/*z*: calcd for C_17_H_22_N_3_O_2_^+^ [M + H]^+^, 300.1707; found, 300.1716.

##### Step 5: 2-Chloro-4-((2-cyclobutyl-7-methyl-6-oxo-1,2,3,4,6,7-hexahydro-[1,4]oxazepino[2,3-*c*]quinolin-10-yl)amino)nicotinonitrile (**11c**)

Prepared from **29f** (17 mg, 0.056 mmol) according to the procedure used for **9a** (step 5). Additional purification by HPLC (55–80% methanol in water, 0.1% formic acid) afforded **11c** as an off-white solid (9 mg, 36%). ^1^H NMR (600 MHz, CDCl_3_): δ 8.07 (d, *J* = 6.1 Hz, 1 H), 7.42 (d, *J* = 8.9 Hz, 1 H), 7.37 (dd, *J* = 8.9, 2.1 Hz, 1 H), 7.28–7.27 (m, 1 H), 6.96 (s, 1 H), 6.64 (d, *J* = 6.1 Hz, 1 H), 4.49–4.43 (m, 1 H), 4.36–4.30 (m, 1 H), 3.80 (br s, 1 H), 3.77–3.72 (m, 4 H), 2.58–2.49 (m, 1 H), 2.26–2.17 (m, 2 H), 2.16–2.10 (m, 1 H), 2.02–1.93 (m, 1 H), 1.91–1.79 (m, 3 H), 1.72–1.65 (m, 1 H); LCMS (4 min; ESI) RT 2.90 min; *m*/*z*: calcd for C_23_H_23_ClN_5_O_2_^+^ [M + H]^+^, 436.1540; found, 436.1548.

#### 2-Chloro-4-((2-isopropyl-7-methyl-6-oxo-1,2,3,4,6,7-hexahydro-[1,4]oxazepino[2,3-*c*]quinolin-10-yl)amino)nicotinonitrile (**11d**)

##### Step 1: 4-((1-Hydroxy-4-methylpentan-3-yl)amino)-1-methyl-6-nitroquinolin-2(1*H*)-one (**26g**)

Prepared from **20a** (150 mg, 0.63 mmol) and 3-amino-4-methylpentan-1-ol (111 mg, 0.94 mmol) using the procedure described for **26a**. Purification was by HPLC (40–100% methanol in water, 0.1% formic acid), affording **26g** (23 mg, 11%) as a deep-yellow solid. ^1^H NMR (500 MHz, DMSO-*d*_6_): δ 9.22 (d, *J* = 2.5 Hz, 1 H), 8.38 (dd, *J* = 9.4, 2.5 Hz, 1 H), 7.61 (d, *J* = 9.4 Hz, 1 H), 7.00 (d, *J* = 8.5 Hz, 1 H), 5.59 (s, 1 H), 4.47 (t, *J* = 4.9 Hz, 1 H), 3.55 (s, 3 H), 3.54–3.46 (m, 2 H), 3.44–3.38 (m, 1 H), 1.95 (dq, *J* = 12.4, 6.3 Hz, 1 H), 1.88–1.71 (m, 2 H), 0.94 (dd, *J* = 8.3, 6.8 Hz, 6 H). LCMS (2 min; ToF) RT 1.36 min; *m*/*z*: calcd for C_16_H_22_N_3_O_4_^+^ [M + H]^+^, 320.1605; found, 320.1579.

##### Step 2: 3-Bromo-4-((1-hydroxy-4-methylpentan-3-yl)amino)-1-methyl-6-nitroquinolin-2(1*H*)-one (**27g**)

Trifluoroacetic acid (28 μL, 0.36 mmol) was added to a solution of **26g** (23 mg, 0.072 mmol) and *N*-bromosuccinimide (19 mg, 0.11 mmol) at 0 °C. The resulting solution was stirred at 0 °C for 10 min and then at rt for 15 min. Volatiles were removed, and the crude was purified by HPLC (40–100% methanol in water, 0.1% formic acid). **27g** (13 mg, 45%) was obtained as a yellow solid after freeze-drying. ^1^H NMR (500 MHz, DMSO-*d*_6_): δ 9.00 (d, *J* = 2.6 Hz, 1 H), 8.42 (dd, *J* = 9.4, 2.6 Hz, 1 H), 7.73 (d, *J* = 9.4 Hz, 1 H), 5.55 (d, *J* = 10.5 Hz, 1 H), 4.50 (t, *J* = 4.7 Hz, 1 H), 4.24–4.15 (m, 1 H), 3.69 (s, 3 H), 3.55 (m, 2 H), 1.97–1.87 (m, 1 H), 1.82–1.68 (m, 2 H), 0.94 (d, *J* = 6.8 Hz, 3 H), 0.90 (d, *J* = 6.8 Hz, 3 H). LCMS (2 min; ToF) RT 1.46 min; *m*/*z*: calcd for C_16_H_21_BrN_3_O_4_^+^ [M + H]^+^, 398.0710; found, 398.0669.

##### Step 3: 2-Isopropyl-7-methyl-10-nitro-1,2,3,4-tetrahydro-[1,4]oxazepino[2,3-*c*]quinolin-6(7*H*)-one (**28g**)

Prepared by cyclization of **27g** (13 mg, 0.033 mmol) using the procedure described for **28a**. The reaction mixture was purified by HPLC (40–100% methanol in water, 0.1% formic acid), affording **28g** as a brown oil (1.5 mg, 14%). ^1^H NMR (500 MHz, CD_3_OD): δ 8.94 (d, *J* = 2.5 Hz, 1 H), 8.38 (dd, *J* = 9.3, 2.5 Hz, 1 H), 7.67 (d, *J* = 9.3 Hz, 1 H), 4.48–4.27 (m, 2 H), 3.75 (s, 3 H), 3.59 (ddd, *J* = 9.4, 7.8, 3.8 Hz, 1 H), 2.32–2.23 (m, 1 H), 2.20–2.08 (m, 1 H), 2.02–1.94 (m, 1 H), 1.11 (d, *J* = 6.6 Hz, 3 H), 1.07 (d, *J* = 6.6 Hz, 3 H). LCMS (2 min; ToF) RT 1.43 min; *m*/*z*: calcd for C_16_H_20_N_3_O_4_^+^ [M + H]^+^, 318.1448; found, 318.1442.

##### Step 4: 10-Amino-2-isopropyl-7-methyl-1,2,3,4-tetrahydro-[1,4]oxazepino[2,3-*c*]quinolin-6(7*H*)-one (**29g**)

A mixture of **28g** (2.5 mg, 0.008 mmol), ammonium formate (3.30 mg, 0.053 mmol), and Pd/C (10 wt %, 0.3 mg) in methanol (3.00 mL) was stirred under microwave irradiation at 80 °C for 10 min. The reaction mixture was allowed to cool to rt, filtered, and purified by SCX-2 to give **29g** (2 mg, 88%, 0.007 mmol) as a pink oil. ^1^H NMR (500 MHz, CD_3_OD): δ 7.34 (d, *J* = 9.0 Hz, 1 H), 7.18 (d, *J* = 2.4 Hz, 1 H), 7.07 (dd, *J* = 9.0, 2.4 Hz, 1 H), 4.41–4.32 (m, 1 H), 4.31–4.22 (m, 1 H), 3.66 (s, 3 H), 3.61–3.55 (m, 1 H), 2.27–2.15 (m, 1 H), 2.11–2.03 (m, 1H), 2.00–1.90 (m, 2H), 1.10 (d, *J* = 6.6 Hz, 3 H), 1.07 (d, *J* = 6.6 Hz, 3 H). LCMS (2 min; ToF) RT 1.00 min; *m*/*z*: calcd for C_16_H_22_N_3_O_2_^+^ [M + H]^+^, 288.1707; found, 288.1701.

##### Step 5: 2-Chloro-4-((2-isopropyl-7-methyl-6-oxo-1,2,3,4,6,7-hexahydro-[1,4]oxazepino[2,3-*c*]quinolin-10-yl)amino)nicotinonitrile (**11d**)

Prepared from **29g** (2 mg, 0.007 mmol) using the procedure described for **7**. Further purification by SCX-2 gave **11d** (0.5 mg, 17%) as a light-brown solid. ^1^H NMR (600 MHz, CD_3_OD): δ 7.99 (d, *J* = 6.2 Hz, 1 H), 7.95 (d, *J* = 2.3 Hz, 1 H), 7.63 (d, *J* = 9.0 Hz, 1 H), 7.52 (dd, *J* = 9.0, 2.3 Hz, 1 H), 6.75 (d, *J* = 6.2 Hz, 1 H), 4.42–4.37 (m, 1 H), 4.37–4.31 (m, 1 H), 3.75 (s, 3 H), 3.64–3.53 (m, 1 H), 2.31–2.20 (m, 1 H), 2.06 (dt, *J* = 13.6, 6.9 Hz, 1 H), 1.99–1.94 (m, 1 H), 1.07 (d, *J* = 6.6 Hz, 3 H), 1.05 (d, *J* = 6.6 Hz, 3 H). LCMS (4 min; ToF) RT 2.80 min; *m*/*z*: calcd for C_22_H_23_ClN_5_O_2_^+^ [M + H]^+^, 424.1535; found, 424.1519.

#### 2-Chloro-4-((2-(difluoromethyl)-7-methyl-6-oxo-1,2,3,4,6,7-hexahydro-[1,4]oxazepino[2,3-*c*]quinolin-10-yl)amino)nicotinonitrile (**11e**)

##### Step 1: 4-((1,1-Difluoro-4-hydroxybutan-2-yl)amino)-1-methyl-6-nitroquinolin-2(1*H*)-one (**26h**)

To a mixture of **20b** (115 mg, 0.37 mmol) and 3-amino-4,4-difluoro-butan-1-ol (69 mg, 0.59 mmol) under argon was added NMP (1.5 mL), followed by DIPEA (0.32 mL, 1.83 mmol). The reaction mixture was heated at 160 °C under microwave irradiation for 2 h. Lithium chloride (30 mg, 0.70 mmol) was added, and the reaction mixture was heated at 160 °C under microwave irradiation for 2 h and then cooled to rt and added to water (15 mL). The resulting aqueous mixture was extracted with EtOAc (3 × 15 mL). The organic extracts were combined, dried (Na_2_SO_4_), and concentrated under reduced pressure and then purified by reverse-phase chromatography (10–80% methanol in water, 0.1% formic acid), affording **26h** (85 mg, 70%) as a pale-yellow solid. ^1^H NMR (500 MHz, DMSO-*d*_6_): δ 9.20 (d, *J* = 2.5 Hz, 1 H), 8.40 (dd, *J* = 9.4, 2.5 Hz, 1 H), 7.64 (d, *J* = 9.4 Hz, 1 H), 7.19 (d, *J* = 8.7 Hz, 1 H), 6.13 (dt, *J* = 55.6 (*J*_*H-F*_), 3.3 Hz, 1 H), 5.81 (s, 1 H), 4.67 (t, *J* = 4.9 Hz, 1 H), 4.25–4.13 (m, 1 H), 3.59–3.52 (m, 4 H), 3.47–3.40 (m, 1 H), 1.98–1.90 (m, 1 H), 1.90–1.83 (m, 1 H); LCMS (2 min; ToF); RT 1.18 min; *m*/*z*: calcd for C_14_H_16_F_2_N_3_O_4_^+^ [M + H]^+^, 328.1103; found, 328.1078.

##### Step 2: 3-Bromo-4-((1,1-difluoro-4-hydroxybutan-2-yl)amino)-1-methyl-6-nitroquinolin-2(1*H*)-one (**27h**)

Trifluoroacetic acid (0.1 mL, 1.3 mmol) was added to a stirred mixture of *N*-bromosuccinimide (69 mg, 0.39 mmol) and **26h** (85 mg, 0.26 mmol) in DCM (1.8 mL) at 0 °C under argon. The reaction mixture was stirred at 0 °C for 10 min and then diluted with EtOAc (20 mL) and washed with saturated aq. NaHCO_3_ (2 × 20 mL). The aqueous washings were further extracted with EtOAc (20 mL). The organic extracts were combined, dried (Na_2_SO_4_), and concentrated under reduced pressure and then purified by flash chromatography (0 to 10% methanol in DCM), affording the title compound (52 mg, 49%) as a pale-yellow solid. ^1^H NMR (500 MHz, DMSO-*d*_6_): δ 9.07 (d, *J* = 2.5 Hz, 1 H), 8.43 (dd, *J* = 9.4, 2.5 Hz, 1 H), 7.75 (d, *J* = 9.4 Hz, 1 H), 6.17 (dt, *J* = 55.6 (*J*_*H-F*_), 3.2 Hz, 1 H), 5.98 (d, *J* = 10.8, 1 H), 4.67 (t, *J* = 4.7 Hz, 1 H), 4.58–4.47 (m, 1 H), 3.71 (s, 3 H), 3.67–3.58 (m, 2 H), 2.01–1.93 (m, 1 H), 1.93–1.85 (m, 1 H); LCMS (2 min; ToF); RT 1.27 min; *m*/*z*: calcd for C_14_H_15_BrF_2_N_3_O_4_^+^ [M + H]^+^, 406.0209; found, 406.0202.

##### Step 3: 2-(Difluoromethyl)-7-methyl-10-nitro-1,2,3,4-tetrahydro-[1,4]oxazepino[2,3-*c*]quinolin-6(7*H*)-one (**28h**)

Prepared by cyclization of **27h** (52 mg, 0.13 mmol) according to the procedure used for **28a**. The reaction time was 65 min, affording **28h** as a yellow solid (16 mg, 38%). ^1^H NMR (500 MHz, DMSO-*d*_6_): δ 9.00 (d, *J* = 2.5 Hz, 1 H), 8.34 (dd, *J* = 9.4, 2.5 Hz, 1 H), 7.64 (d, *J* = 9.4 Hz, 1 H), 6.88 (d, *J* = 5.7 Hz, 1 H), 6.34 (dt, *J* = 56.5 (*J*_H-F_), 5.6 Hz, 1 H), 4.30–4.19 (m, 2 H), 4.02–3.92 (m, 1 H), 3.62 (s, 3 H), 2.33–2.25 (m, 1 H), 2.17–2.10 (m, 1 H); LCMS (2 min; ToF); RT 1.24 min; *m*/*z*: calcd for C_14_H_14_F_2_N_3_O_4_^+^ [M + H]^+^, 326.0947; found, 326.0971.

##### Step 4: 10-Amino-2-(difluoromethyl)-7-methyl-1,2,3,4-tetrahydro-[1,4]oxazepino[2,3-*c*]quinolin-6(7*H*)-one (**29h**)

Prepared from **28h** (16 mg, 0.05 mmol), according to the procedure used for **29a**. The reaction time was 20 min, affording **29h** (14 mg, 96%) as a dark-yellow solid. LCMS (2 min; ESI) RT 0.61 min; *m*/*z*: calcd for C_14_H_16_F_2_N_3_O_2_^+^ [M + H]^+^, 296.1205; found, 296.1224.

##### Step 5: 2-Chloro-4-((2-(difluoromethyl)-7-methyl-6-oxo-1,2,3,4,6,7-hexahydro-[1,4]oxazepino[2,3-*c*]quinolin-10-yl)amino)nicotinonitrile (**11e**)

Prepared from **29h** (7 mg, 0.025 mmol) according to the procedure used for **9a**, affording **11e** as an off-white solid (6 mg, 52%). ^1^H NMR (600 MHz, CDCl_3_): δ 8.08 (d, *J* = 6.1 Hz, 1 H), 7.45–7.40 (m, 3 H), 6.94 (br s, 1 H), 6.63 (d, *J* = 6.1 Hz, 1 H), 6.04 (dt, *J* = 55.8 Hz (*J*_*H-F*_), 4.8 Hz, 1 H), 4.53–4.44 (m, 2 H), 4.24 (br d, *J* = 3.4 Hz, 1 H), 4.14–4.05 (m, 1 H), 3.75 (s, 3 H), 2.40–2.33 (m, 1 H), 2.25–2.18 (m, 1 H); LCMS (4 min; ToF) RT 2.56 min; *m*/*z*: calcd for C_20_H_17_ClF_2_N_5_O_2_^+^ [M + H]^+^, 432.1033; found, 432.1027.

#### (*R*)-2-Chloro-4-((2-cyclopropyl-7-methyl-6-oxo-1,2,3,4,6,7-hexahydro-[1,4]oxazepino[2,3-*c*]quinolin-10-yl)amino)nicotinonitrile (**12a**)

##### Step 1: (*R*)-4-((1-Cyclopropyl-3-hydroxypropyl)amino)-1-methyl-6-nitroquinolin-2(1*H*)-one (**26i**)

Method A: According to the same procedure as **26a**, reaction time 4 d at 160 °C, from **20a** (500 mg, 2.1 mmol) and (*R*)-3-amino-3-cyclopropylpropan-1-ol (541 mg, 3.6 mmol), **26i** obtained as a yellow solid (298 mg, 45%).

Method B: (*R*)-3-Amino-3-cyclopropylpropan-1-ol (0.79 g, 6.8 mmol), **20b** (1.51 g, 4.8 mmol), DIPEA (2.1 mL, 12.1 mmol), and anhydrous acetonitrile (24 mL) in a dry flask under argon were heated at 85 °C for 22 h. The reaction mixture was concentrated under reduced pressure to remove MeCN. THF (15 mL) was added, followed by sodium hydroxide (2 M, 14 mL, 28 mmol). The reaction mixture was heated at 85 °C for 6 h. The reaction mixture was cooled to rt, diluted with water (30 mL), and acidified to pH 5 with 3 M HCl. The resulting precipitate was filtered, washed with water (70 mL), and dried, affording **26i** (1.48 g, 96%) as a dark-yellow solid. ^1^H NMR (500 MHz, DMSO-*d*_6_): δ 9.15 (d, *J* = 2.5 Hz, 1 H), 8.38 (dd, *J* = 9.4, 2.5 Hz, 1 H), 7.60 (d, *J* = 9.4 Hz, 1 H), 7.17 (d, *J* = 8.3 Hz, 1 H), 5.59 (s, 1 H), 4.51 (t, *J* = 5.0 Hz, 1 H), 3.59–3.53 (m, 4 H), 3.51–3.45 (m, 1 H), 3.27–3.22 (m, 1 H), 1.95–1.87 (m, 1 H), 1.84–1.77 (m, 1 H), 1.14–1.07 (m, 1 H), 0.53–0.47 (m, 1 H), 0.43–0.37 (m, 1 H), 0.28–0.23 (m, 2 H); LCMS (2 min; ESI) RT 1.17 min; *m*/*z*: calcd for C_16_H_20_N_3_O_4_^+^ [M + H]^+^, 318.1448; found, 318.1452.

##### Step 2: (*R*)-3-Bromo-4-((1-cyclopropyl-3-hydroxypropyl)amino)-1-methyl-6-nitroquinolin-2(1*H*)-one (**27i**)

Trifluoroacetic acid (1.8 mL, 23.5 mmol) was added to a stirred mixture of *N*-bromosuccinimide (1.25 g, 7 mmol) and **26i** (1.48 g, 4.67 mmol) in DCM (30 mL) at 0 °C under Ar. The reaction mixture was stirred at 0 °C for 15 min and then diluted with DCM (30 mL) and washed with saturated aq. NaHCO_3_ (2 × 50 mL). The aqueous washings were further extracted with DCM (30 mL). The organic extracts were combined, washed with brine (50 mL), dried (Na_2_SO_4_), and concentrated under reduced pressure. The resulting product was recrystallized from ethanol to give 498 mg of the desired product; the filtrate was dry-loaded onto silica, and purification by flash chromatography (0 to 10% methanol in DCM) afforded a further 582 mg of the desired product. Product batches were combined to give **27i** (1.08 g, 58%) as a yellow solid. ^1^H NMR (500 MHz, DMSO-*d*_6_): δ 8.94 (d, *J* = 2.6 Hz, 1 H), 8.41 (dd, *J* = 9.4, 2.6 Hz, 1 H), 7.73 (d, *J* = 9.4 Hz, 1 H), 5.67 (d, *J* = 10.3 Hz, 1 H), 4.52 (t, *J* = 4.7 Hz, 1 H), 3.70 (s, 3 H), 3.65–3.60 (m, 2 H), 3.56–3.48 (m, 1 H), 2.01–1.87 (m, 2 H), 1.15–1.06 (m, 1 H), 0.48–0.40 (m, 1 H), 0.35–0.28 (m, 1 H), 0.23–0.17 (m, 1 H), 0.08–0.02 (m, 1 H); LCMS (2 min; ESI) RT 1.25 min; *m*/*z*: calcd for C_16_H_19_BrN_3_O_4_^+^ [M + H]^+^, 396.0553; found, 396.0584.

##### Step 3: (*R*)-2-Cyclopropyl-7-methyl-10-nitro-1,2,3,4-tetrahydro-[1,4]oxazepino[2,3-*c*]quinolin-6(7*H*)-one (**28i**)

Method A: To **27i** (374 mg, 0.94 mmol) under argon was added DMSO (12.6 mL), followed by potassium *tert*-butoxide (1 M in THF, 1.7 mL, 1.7 mmol), and the reaction mixture was heated at 60 °C under microwave irradiation for 50 min. To a further portion of **27i** (290 mg, 0.73 mmol) under argon was added DMSO (9.8 mL), followed by potassium *tert*-butoxide (1 M in THF, 1.32 mL, 1.32 mmol), and the reaction mixture was heated at 60 °C under microwave irradiation for 50 min. The reaction mixtures were combined, and water (100 mL) was added, followed by EtOAc (100 mL). The layers were separated, and the aqueous layer was further extracted with EtOAc (2 × 100 mL). The organic extracts were combined and concentrated under reduced pressure. The crude product was dissolved in DMSO (1.5 mL) and purified by reverse-phase chromatography (45–65% methanol in water, 0.1% formic acid) to give **28i** (247 mg, 47%) as a yellow solid.

Method B: Lithium *tert*-butoxide (1 M in THF; 2.35 mL, 2.35 mmol) was added to a suspension of **27i** (582 mg, 1.47 mmol) in anhydrous THF (14.7 mL) under Ar. A reflux condenser was fitted, and the reaction mixture was heated at 60 °C for 15 min. The reaction mixture was cooled to rt. Water (10 mL) was added, and the aqueous mixture was extracted with DCM (4 × 10 mL). The organic extracts were combined, washed with brine (2 × 10 mL), dried (Na_2_SO_4_), and concentrated under reduced pressure, affording **28i** (463 mg, 99%) as an orange solid. ^1^H NMR (500 MHz, DMSO-*d*_6_): δ 9.03 (d, *J* = 2.6 Hz, 1 H), 8.31 (dd, *J* = 9.4, 2.6 Hz, 1 H), 7.60 (d, *J* = 9.4 Hz, 1 H), 6.62 (d, *J* = 4.4 Hz, 1 H), 4.26–4.18 (m, 2 H), 3.60 (s, 3 H), 2.94–2.87 (m, 1 H), 2.25–2.18 (m, 1 H), 2.05–1.99 (m, 1 H), 1.29–1.23 (m, 1 H), 0.57–0.49 (m, 2 H), 0.41–0.36 (m, 1 H), 0.30–0.24 (m, 1 H); LCMS (2 min; ESI) RT 1.25 min; *m*/*z*: calcd for C_16_H_18_N_3_O_4_^+^ [M + H]^+^, 316.1292; found, 316.1304.

##### Step 4: (*R*)-10-Amino-2-cyclopropyl-7-methyl-1,2,3,4-tetrahydro-[1,4]oxazepino[2,3-*c*]quinolin-6(7*H*)-one (**29i**)

**28i** (463 mg, 1.47 mmol), Pd/C (10 wt %, 46 mg), and ethanol (10 mL) were stirred at 60 °C under an atmosphere of H_2_ for 2 h. The reaction mixture was allowed to cool to rt. The reaction mixture was filtered through Celite, and the solids were washed with EtOH (50 mL). The filtrate was concentrated under reduced pressure, affording **29i** (414 mg, 99%) as an orange solid. LCMS (2 min; ESI) RT 0.76 min; *m*/*z*: calcd for C_16_H_20_N_3_O_2_^+^ [M + H]^+^, 286.1550; found, 286.1563.

##### Step 5: (*R*)-2-Chloro-4-((2-cyclopropyl-7-methyl-6-oxo-1,2,3,4,6,7-hexahydro-[1,4]oxazepino[2,3-*c*]quinolin-10-yl)amino)nicotinonitrile (**12a**)

To **29i** (12 mg, 0.042 mmol) and 2,4-dichloropyridine-3-carbonitrile (10.4 mg, 0.06 mmol) under argon was added NMP (0.6 mL), followed by TEA (18 μL, 0.129 mmol). The reaction mixture was heated to 160 °C under microwave irradiation for 90 min and then was diluted with DMSO (0.8 mL) and purified using reverse-phase chromatography (10–80% methanol in water, 0.1% formic acid), affording **12a** (9.1 mg, 51%) as an off-white solid. ^1^H NMR (600 MHz, CD_3_OD): δ 7.99 (d, *J* = 6.2 Hz, 1 H), 7.95 (d, *J* = 2.1 Hz, 1 H), 7.61 (d, *J* = 8.9 Hz, 1 H), 7.51 (dd, *J* = 8.9, 2.1 Hz, 1 H), 6.74 (d, *J* = 6.2 Hz, 1 H), 4.41–4.35 (m, 1 H), 4.24–4.19 (m, 1 H), 3.72 (s, 3 H), 2.92 (td, *J* = 9.4, 3.7 Hz, 1 H), 2.37–2.29 (m, 1 H), 2.12–2.06 (m, 1 H), 1.24–1.16 (m, 1 H), 0.66–0.57 (m, 2 H), 0.39–0.34 (m, 1 H), 0.32–0.28 (m, 1 H); LCMS (4 min; ESI) RT 2.57 min; *m*/*z*: calcd for C_22_H_21_ClN_5_O_2_^+^ [M + H]^+^, 422.1378; found, 422.1383.

#### (*S*)-2-Chloro-4-((2-cyclopropyl-7-methyl-6-oxo-1,2,3,4,6,7-hexahydro-[1,4]oxazepino[2,3-*c*]quinolin-10-yl)amino)nicotinonitrile (**12b**)

##### Step 1: (*S*)-4-((1-Cyclopropyl-3-hydroxypropyl)amino)-1-methyl-6-nitroquinolin-2(1*H*)-one (**26j**)

Prepared as for **26i** (**12a** step 1, method B), from **20b** (800 mg, 2.57 mmol) and (*S*)-3-amino-3-cyclopropylpropan-1-ol (356 mg, 3.09 mmol). Additional purification by flash chromatography was required to afford the title compound as a yellow solid (576 mg, 70%). ^1^H NMR (500 MHz, DMSO-*d*_6_): δ 9.15 (d, *J* = 2.5 Hz, 1 H), 8.38 (dd, *J* = 9.4, 2.5 Hz, 1 H), 7.60 (d, *J* = 9.4 Hz, 1 H), 7.17 (d, *J* = 8.3 Hz, 1 H), 5.59 (s, 1 H), 4.51 (br s, 1 H), 3.59–3.53 (m, 4 H), 3.51–3.45 (m, 1 H), 3.27–3.22 (m, 1 H), 1.95–1.87 (m, 1 H), 1.84–1.77 (m, 1 H), 1.14–1.07 (m, 1 H), 0.53–0.47 (m, 1 H), 0.43–0.37 (m, 1 H), 0.28–0.23 (m, 2 H); LCMS (2 min; ESI) RT 1.18 min; *m*/*z*: calcd for C_16_H_20_N_3_O_4_^+^ [M + H]^+^, 318.1448; found, 318.1451.

##### Step 2: (*S*)-3-Bromo-4-((1-cyclopropyl-3-hydroxypropyl)amino)-1-methyl-6-nitroquinolin-2(1*H*)-one (**27j**)

Prepared by bromination of **26j** (77 mg, 0.24 mmol) using the procedure described for **27a** to give **27j** as a yellow solid (68 mg, 71%). ^1^H NMR (500 MHz, DMSO-*d*_6_): δ 8.94 (d, *J* = 2.6 Hz, 1 H), 8.41 (dd, *J* = 9.4, 2.6 Hz, 1 H), 7.73 (d, *J* = 9.4 Hz, 1 H), 5.66 (d, *J* = 10.3 Hz, 1 H), 4.52 (t, *J* = 4.7 Hz, 1 H), 3.70 (s, 3 H), 3.65–3.60 (m, 2 H), 3.56–3.48 (m, 1 H), 2.01–1.87 (m, 2 H), 1.15–1.06 (m, 1 H), 0.48–0.40 (m, 1 H), 0.35–0.28 (m, 1 H), 0.23–0.17 (m, 1 H), 0.08–0.02 (m, 1 H); LCMS (2 min; ESI) RT 1.25 min; *m*/*z*: calcd for C_16_H_19_BrN_3_O_4_^+^ [M + H]^+^, 396.0553; found, 396.0580.

##### Step 3: (*S*)-2-Cyclopropyl-7-methyl-10-nitro-1,2,3,4-tetrahydro-[1,4]oxazepino[2,3-*c*]quinolin-6(7*H*)-one (**28j**)

Prepared from **27j** (68 mg, 0.17 mmol) using the procedure described for **28a**. Obtained **28j** as a dark-yellow solid (20 mg, 37%). ^1^H NMR (500 MHz, DMSO-*d*_6_): δ 9.03 (d, *J* = 2.6 Hz, 1 H), 8.31 (dd, *J* = 9.4, 2.6 Hz, 1 H), 7.60 (d, *J* = 9.4 Hz, 1 H), 6.62 (d, *J* = 4.4 Hz, 1 H), 4.26–4.18 (m, 2 H), 3.60 (s, 3 H), 2.94–2.87 (m, 1 H), 2.25–2.18 (m, 1 H), 2.05–1.99 (m, 1 H), 1.29–1.23 (m, 1 H), 0.57–0.49 (m, 2 H), 0.41–0.36 (m, 1 H), 0.30–0.24 (m, 1 H); LCMS (2 min; ToF) RT 1.37 min; *m*/*z*: calcd for C_16_H_18_N_3_O_4_^+^ [M + H]^+^, 316.1292; found, 316.1289.

##### Step 4: (*S*)-10-Amino-2-cyclopropyl-7-methyl-1,2,3,4-tetrahydro-[1,4]oxazepino[2,3-*c*]quinolin-6(7*H*)-one (**29j**)

Prepared by transfer hydrogenation of **28j** (20 mg, 0.06 mmol) using the procedure described for **29a**. Obtained a yellow solid (17 mg, 92%). LCMS (2 min; ToF) RT 0.92 min; *m*/*z*: calcd for C_16_H_20_N_3_O_2_^+^ [M + H]^+^, 286.1550; found, 286.1554.

##### Step 5: (*S*)-2-Chloro-4-((2-cyclopropyl-7-methyl-6-oxo-1,2,3,4,6,7-hexahydro-[1,4]oxazepino[2,3-*c*]quinolin-10-yl)amino)nicotinonitrile (**12b**)

Prepared from **29j** (5 mg, 0.018 mmol) using the procedure described for **12a** to give the title compound as an off-white solid (5 mg, 71%). ^1^H NMR (600 MHz, CD_3_OD): δ 7.99 (d, *J* = 6.2 Hz, 1 H), 7.95 (d, *J* = 2.1 Hz, 1 H), 7.61 (d, *J* = 8.9 Hz, 1 H), 7.51 (dd, *J* = 8.9, 2.1 Hz, 1 H), 6.74 (d, *J* = 6.2 Hz, 1 H), 4.41–4.35 (m, 1 H), 4.24–4.19 (m, 1 H), 3.72 (s, 3 H), 2.92 (td, *J* = 9.4, 3.7 Hz, 1 H), 2.37–2.29 (m, 1 H), 2.12–2.06 (m, 1 H), 1.24–1.16 (m, 1 H), 0.66–0.57 (m, 2 H), 0.39–0.34 (m, 1 H), 0.32–0.28 (m, 1 H); LCMS (4 min; ToF) RT 2.73 min; *m*/*z*: calcd for C_22_H_21_ClN_5_O_2_^+^ [M + H]^+^, 422.1378; found, 422.1369.

#### 2-Chloro-4-(((2*S*,4*S*)-2,4,7-trimethyl-6-oxo-1,2,3,4,6,7-hexahydro-[1,4]oxazepino[2,3-*c*]quinolin-10-yl)amino)nicotinonitrile (**13a**)

##### Step 1: 4-(((2*S*,4*S*)-4-Hydroxypentan-2-yl)amino)-1-methyl-6-nitroquinolin-2(1*H*)-one (**26k**)

Prepared as for **26a** starting from (2*S*,4*S*)-4-aminopentan-2-ol (31 mg, 0.30 mmol)^28,29^ with a 40 h reaction time at 160 °C. **26k** (41 mg, 63%, 0.1340 mmol) was obtained as a brown solid after purification by flash chromatography (0–10% methanol in DCM). ^1^H NMR (500 MHz, CDCl_3_): δ 8.52 (d, *J* = 2.4 Hz, 1 H), 8.40 (dd, *J* = 9.4, 2.4 Hz, 1 H), 7.44 (d, *J* = 9.4 Hz, 1 H), 6.81 (br s, 1 H), 5.99 (s, 1 H), 4.42–4.34 (m, 1 H), 4.01–3.93 (m, 1 H), 3.72 (s, 3 H), 1.93 (ddd, *J* = 14.7, 9.9, 3.9 Hz, 1 H), 1.79 (dd, *J* = 14.7, 5.7, 2.3 Hz, 1 H), 1.37 (d*, J* = 6.6 Hz, 3 H), 1.34 (d*, J* = 6.2 Hz, 3 H). LCMS (2 min; ToF); RT 1.27 min; *m*/*z*: calcd for C_15_H_20_N_3_O_4_^+^ [M + H]^+^, 306.1448; found, 306.1444.

##### Step 2: 3-Bromo-4-(((2*S*,4*S*)-4-hydroxypentan-2-yl)amino)-1-methyl-6-nitroquinolin-2(1*H*)-one (**27k**)

Prepared from **26k** using the procedure described for **27a**. **27k** (22 mg, 44%) was obtained as a yellow solid. ^1^H NMR (500 MHz, DMSO-*d*_6_): δ 8.92 (d, *J* = 2.5 Hz, 1 H), 8.42 (dd, *J* = 9.4, 2.5 Hz, 1 H), 7.72 (d, *J* = 9.4 Hz, 1 H), 5.91 (d, *J* = 9.8 Hz, 1 H), 4.55 (d, *J* = 4.9 Hz, 1 H), 4.35–4.25 (m, 1 H), 3.78–3.71 (m, 1 H), 3.68 (s, 3 H), 1.82–1.76 (m, 1 H), 1.63–1.56 (m, 1 H), 1.29 (d, *J* = 6.5 Hz, 3 H), 1.05 (d, *J* = 6.1 Hz, 3 H); LCMS (2 min; ESI) RT 1.26 min; *m*/*z*: calcd for C_15_H_19_BrN_3_O_4_^+^ [M + H]^+^, 384.0553; found, 384.0563.

##### Step 3: (2*S*,4*S*)-2,4,7-Trimethyl-10-nitro-1,2,3,4-tetrahydro-[1,4]oxazepino[2,3-*c*]quinolin-6(7*H*)-one (**28k**)

Prepared from **27k** using the procedure described for **28a**. After 2 h of heating, a 4:6 ratio mixture of the cyclized product to the dehalogenated starting material was obtained. The desired product **28k** was isolated by reverse-phase chromatography (45–65% methanol in water, 0.1% formic acid) as a yellow solid (2 mg, 12%). ^1^H NMR (500 MHz, CDCl_3_): δ 8.40 (d, *J* = 2.4 Hz, 1 H), 8.32 (dd, *J* = 9.3, 2.4 Hz, 1 H), 7.38 (d, *J* = 9.3 Hz, 1 H), 4.60–4.53 (m, 1 H), 4.27–4.19 (m, 1 H), 3.95 (br s, 1 H), 3.75 (s, 3 H), 2.05–1.94 (m, 2 H), 1.46 (d, *J* = 6.3 Hz, 3 H), 1.42 (d, *J* = 6.6 Hz, 3 H); LCMS (2 min; ESI); RT 1.26 min; *m*/*z*: calcd for C_15_H_18_N_3_O_4_^+^ [M + H]^+^, 304.1292; found, 304.1294.

##### Step 4: (2*S*,4*S*)-10-Amino-2,4,7-trimethyl-1,2,3,4-tetrahydro-[1,4]oxazepino[2,3-*c*]quinolin-6(7*H*)-one (**29k**)

Prepared from **28k** using the procedure described for **29a**. **29k** (2 mg, 100%) was obtained as dark-yellow solid. LCMS (2 min; ToF); RT 0.79 min; *m*/*z*: calcd for C_15_H_20_N_3_O_2_^+^ [M + H]^+^, 274.1550; found, 274.1552.

##### Step 5: 2-Chloro-4-(((2*S*,4*S*)-2,4,7-trimethyl-6-oxo-1,2,3,4,6,7-hexahydro-[1,4]oxazepino[2,3-*c*]quinolin-10-yl)amino)nicotinonitrile (**13a**)

Prepared from **29k** using the procedure described for 9a. **13a** (1 mg, 33%) was obtained as an off-white solid. ^1^H NMR (600 MHz, CD_3_OD): δ 7.99 (d, *J* = 6.2 Hz, 1 H), 7.94 (d, *J* = 2.2 Hz, 1 H), 7.62 (d, *J* = 8.9 Hz, 1 H), 7.50 (dd, *J* = 8.9, 2.2 Hz, 1 H), 6.70 (d, *J* = 6.2 Hz, 1 H), 4.53–4.47 (m, 1 H), 4.23–4.17 (m, 1 H), 3.74 (s, 3 H), 2.06–2.00 (m, 1 H), 1.98–1.93 (m, 1 H), 1.41 (d, *J* = 6.3 Hz, 3 H), 1.36 (d, *J* = 6.7 Hz, 3 H). LCMS (4 min; ESI) RT 2.70 min; *m*/*z*: calcd for C_21_H_21_ClN_5_O_2_^+^ [M + H]^+^, 410.1384; found, 410.1389.

#### 2-Chloro-4-((2,4,4,7-tetramethyl-6-oxo-1,2,3,4,6,7-hexahydro-[1,4]oxazepino[2,3-*c*]quinolin-10-yl)amino)nicotinonitrile (**13b**)

##### Step 1: 4-((4-Hydroxy-4-methylpentan-2-yl)amino)-1-methyl-6-nitroquinolin-2(1*H*)-one (**26l**)

Prepared as for **26a** starting from 4-amino-2-methylpentan-2-ol hydrochloride (105 mg, 0.68 mmol) with a 40 h reaction time at 160 °C. **26l** (78 mg, 71%) was obtained as a red-brown solid. ^1^H NMR (500 MHz, DMSO-*d*_6_): δ 9.00 (d, *J* = 2.5 Hz, 1 H), 8.37 (dd, *J* = 9.4, 2.5 Hz, 1 H), 7.60 (d, *J* = 9.4 Hz, 1 H), 7.17 (d, *J* = 7.3 Hz, 1 H), 5.61 (s, 1 H), 4.51 (s, 1 H), 3.82–3.75 (m, 1 H), 3.55 (s, 3 H), 1.97 (dd, *J* = 14.3, 7.6 Hz, 1 H), 1.60 (dd, *J* = 14.3, 4.4 Hz, 1 H), 1.22 (d, *J* = 6.4 Hz, 3 H), 1.12 (s, 6 H); LCMS (2 min; ToF) RT 1.21 min; *m*/*z*: calcd for C_16_H_22_N_3_O_4_^+^ [M + H]^+^, 320.1605; found, 320.1601 [M + H]^+^.

##### Step 2: 3-Bromo-4-((4-hydroxy-4-methylpentan-2-yl)amino)-1-methyl-6-nitroquinolin-2(1*H*)-one (**27l**)

Prepared from **26l** using the procedure described for **27a**. **27l** (68 mg, 71%) was obtained as a dark-yellow solid. ^1^H NMR (500 MHz, DMSO-*d*_6_): δ 8.92 (d, *J* = 2.5 Hz, 1 H), 8.41 (dd, *J* = 9.4, 2.5 Hz, 1 H), 7.71 (d, *J* = 9.4 Hz, 1 H), 6.14 (d, *J* = 8.9 Hz, 1 H), 4.50–4.43 (m, 2 H), 3.68 (s, 3 H), 1.88 (dd, *J* = 14.3, 6.9 Hz, 1 H), 1.72 (dd, *J* = 14.3, 5.2 Hz, 1 H), 1.27 (d, *J* = 6.4 Hz, 3 H), 1.14 (s, 3 H), 1.10 (s, 3 H); LCMS (2 min; ToF) RT 1.32 min; *m*/*z*: calcd for C_16_H_21_BrN_3_O_4_^+^ [M + H]^+^, 398.0710; found, 398.0710 [M + H]^+^.

##### Step 3: 2,4,4,7-Tetramethyl-10-nitro-1,2,3,4-tetrahydro-[1,4]oxazepino[2,3-*c*]quinolin-6(7*H*)-one (**28l**)

Prepared from **27l** using the procedure described for **28a**. After an additional 2.5 h of heating at 80 °C, a 1:4 ratio of cyclized product **28l** to dehalogenated starting material **26l** was obtained. The mixture was purified by reverse-phase chromatography (45–65% methanol in water, 0.1% formic acid) to give **28l** as a dark-yellow solid (4 mg, 7%). ^1^H NMR (500 MHz, CD_3_OD): δ 8.91 (d, *J* = 2.6 Hz, 1 H), 8.38 (dd, *J* = 9.4, 2.6 Hz, 1 H), 7.64 (d, *J* = 9.4 Hz, 1 H), 4.50–4.44 (m, 1 H), 3.73 (s, 3 H), 2.14–2.09 (m, 1 H), 1.88–1.84 (m, 1 H), 1.46 (s, 3 H), 1.38 (d, *J* = 6.6 Hz, 3 H), 1.33 (s, 3 H); LCMS (2 min; ToF) RT 1.30 min; *m*/*z*: calcd for C_16_H_20_N_3_O_4_^+^ [M + H]^+^, 318.1448; found, 318.1458.

##### Step 4: 10-Amino-2,4,4,7-tetramethyl-1,2,3,4-tetrahydro-[1,4]oxazepino[2,3-*c*]quinolin-6(7*H*)-one (**29l**)

Prepared from **28l** using the procedure described for **29a**. **29l** (3 mg, 88%) was obtained and used without further purification. LCMS (2 min; ToF) RT 0.98 min; *m*/*z*: calcd for C_16_H_22_N_3_O_2_^+^ [M + H]^+^, 288.1707; found, 288.1684.

##### Step 5: 2-Chloro-4-((2,4,4,7-tetramethyl-6-oxo-1,2,3,4,6,7-hexahydro-[1,4]oxazepino[2,3-*c*]quinolin-10-yl)amino)nicotinonitrile (**13b**)

Prepared from **29l** using the procedure described for **9a**. **13b** (1 mg, 24%) was obtained as an off-white solid. ^1^H NMR (600 MHz, DMSO-*d*_6_): δ 9.57 (br s, 1 H), 8.00 (d, *J* = 5.5 Hz, 1 H), 7.94 (s, 1 H), 7.47–7.40 (m, 2 H), 6.60 (d, *J* = 6.1 Hz, 1 H), 5.66 (s, 1 H), 4.31–4.24 (m, 1 H), 3.55 (s, 3 H), 1.95 (dd, *J* = 14.6, 9.9 Hz, 1 H), 1.74 (d, *J* = 14.6 Hz, 1 H), 1.34 (s, 3 H), 1.25–1.22 (m, 6 H); LCMS (4 min; ToF) RT 2.78 min; *m*/*z*: calcd for C_22_H_23_ClN_5_O_2_^+^ [M + H]^+^, 424.1535; found, 424.1530.

#### 2-Chloro-4-((2,3,3,7-tetramethyl-6-oxo-1,2,3,4,6,7-hexahydro-[1,4]oxazepino[2,3-*c*]quinolin-10-yl)amino)nicotinonitrile (**13c**)

##### Step 1: 4-((4-Hydroxy-3,3-dimethylbutan-2-yl)amino)-1-methyl-6-nitroquinolin-2(1*H*)-one (**26m**)

Prepared from 3-amino-2,2-dimethylbutan-1-ol hydrochloride (146 mg, 0.95 mmol) using the procedure described for **26a**. Obtained **26m** (90 mg, 58%) as a yellow solid. ^1^H NMR (500 MHz, DMSO-*d*_6_): δ 8.84 (d, *J* = 2.5 Hz, 1 H), 8.38 (dd, *J* = 9.4, 2.5 Hz, 1 H), 7.61 (d, *J* = 9.4 Hz, 1 H), 7.12 (d, *J* = 8.7 Hz, 1 H), 5.65 (s, 1 H), 5.21 (t, *J* = 4.8 Hz, 1 H), 3.70–3.63 (m, 1 H), 3.55 (s, 3 H), 3.46 (dd, *J* = 10.6, 4.8 Hz, 1 H), 3.26 (dd, *J* = 10.6, 4.8 Hz, 1 H), 1.17 (d, *J* = 6.6 Hz, 3 H), 0.97 (s, 3 H), 0.87 (s, 3 H); LCMS (2 min; ToF); RT 1.36 min; *m*/*z*: calcd for C_16_H_22_N_3_O_4_^+^ [M + H]^+^, 320.1605; found, 320.1602.

##### Step 2: 3-Bromo-4-((4-hydroxy-3,3-dimethylbutan-2-yl)amino)-1-methyl-6-nitroquinolin-2(1*H*)-one (**27m**)

Prepared from **26m** using the procedure described for **27a**. **27m** (44 mg, 41%) was obtained as a yellow solid. ^1^H NMR (500 MHz, DMSO-*d*_6_): δ 8.83 (d, *J* = 2.5 Hz, 1 H), 8.41 (dd, *J* = 9.4, 2.5 Hz, 1 H), 7.73 (d, *J* = 9.4 Hz, 1 H), 6.29–6.21 (m, 1 H), 5.24–5.18 (m, 1 H), 4.12–4.04 (m, 1 H), 3.68 (s, 3 H), 3.49 (dd, *J* = 10.4, 4.3 Hz, 1 H), 3.23 (dd, *J* = 10.4, 4.1 Hz, 1 H), 1.27 (d, *J* = 6.6 Hz, 3 H), 1.02 (s, 3 H), 0.85 (s, 3 H); LCMS (2 min; ToF); RT 1.47 min; *m*/*z*: calcd for C_16_H_21_BrN_3_O_4_^+^ [M + H]^+^, 398.0700; found, 398.0688.

##### Step 3: 2,3,3,7-Tetramethyl-10-nitro-1,2,3,4-tetrahydro-[1,4]oxazepino[2,3-*c*]quinolin-6(7*H*)-one (**28m**)

Prepared from **27m** using the procedure described for **28a**. **28m** (11 mg, 32%) was obtained as a yellow solid. ^1^H NMR (500 MHz, DMSO-*d*_6_): δ 9.03 (d, *J* = 2.6 Hz, 1 H), 8.28 (dd, *J* = 9.4, 2.6 Hz, 1 H), 7.60 (d, *J* = 9.4 Hz, 1 H), 6.20 (d, *J* = 5.0 Hz, 1 H), 3.86 (s, 2 H), 3.68–3.65 (m, 1 H), 3.60 (s, 3 H), 1.21 (d, *J* = 7.0 Hz, 3 H), 1.06 (s, 3 H), 0.83 (s, 3 H); LCMS (2 min; ToF); RT 1.42 min; *m*/*z*: calcd for C_16_H_20_N_3_O_4_^+^ [M + H]^+^, 318.1448; found, 318.1444.

##### Step 4: 10-Amino-2,3,3,7-tetramethyl-1,2,3,4-tetrahydro-[1,4]oxazepino[2,3-*c*]quinolin-6(7*H*)-one (**29m**)

Prepared from **28m** using the procedure described for **29a**. **29m** (7 mg, 71%) was obtained as a yellow solid. LCMS (2 min; ToF) RT 0.96 min; *m*/*z*: calcd for C_16_H_22_N_3_O_2_^+^ [M + H]^+^, 288.1707; found, 288.1704.

##### Step 5: 2-Chloro-4-((2,3,3,7-tetramethyl-6-oxo-1,2,3,4,6,7-hexahydro-[1,4]oxazepino[2,3-*c*]quinolin-10-yl)amino)nicotinonitrile (**13c**)

Prepared from **29m** using the procedure described for **9a**. **13c** (5 mg, 80%) was obtained as an off-white solid. ^1^H NMR (600 MHz, CDCl_3_): δ 8.09–8.07 (m, 1 H), 7.41 (d, *J* = 8.8 Hz, 1 H), 7.39–7.36 (m, 1 H), 7.32 (br s, 1 H), 6.94 (br s, 1 H), 6.63 (d, *J* = 6.0 Hz, 1 H), 4.07 (d, *J* = 11.9 Hz, 1 H), 4.05 (d, *J* = 11.9 Hz, 1 H), 3.87–3.81 (m, 1 H), 3.74 (s, 3 H), 3.62 (br s, 1 H), 1.25 (d, *J* = 6.8 Hz, 3 H), 1.15 (s, 3 H), 0.87 (s, 3 H); LCMS (4 min; ToF) RT 2.80 min; *m*/*z*: calcd for C_22_H_23_ClN_5_O_2_^+^ [M + H]^+^, 424.1535; found, 424.1510.

#### 2-Chloro-4-((2′,7′-dimethyl-6′-oxo-1′,2′,6′,7′-tetrahydro-4′*H*-spiro[cyclopropane-1,3′-[1,4]oxazepino[2,3-*c*]quinolin]-10′-yl)amino)nicotinonitrile (**13d**)

##### Step 1: 4-((1-(1-(Hydroxymethyl)cyclopropyl)ethyl)amino)-1-methyl-6-nitroquinolin-2(1*H*)-one (**26n**)

Prepared from (1-(1-aminoethyl)cyclopropyl)methanol hydrochloride (144 mg, 0.95 mmol) using the procedure described for **26a**. **26n** (105 mg, 68%) was obtained as a beige solid. ^1^H NMR (500 MHz, DMSO-*d*_6_): δ 8.91 (d, *J* = 2.5 Hz, 1 H), 8.37 (dd, *J* = 9.3, 2.5 Hz, 1 H), 7.60 (d, *J* = 9.3 Hz, 1 H), 7.06 (d, *J* = 7.4 Hz, 1 H), 5.66 (s, 1 H), 5.12 (t, *J* = 5.3 Hz, 1 H), 3.72–3.66 (m, 1 H), 3.55 (s, 3 H), 3.47–3.38 (m, 2 H), 1.31 (d, *J* = 6.6 Hz, 3 H), 0.54–0.48 (m, 1 H), 0.41–0.36 (m, 1 H), 0.35–0.29 (m, 2 H); LCMS (2 min; ToF); RT 1.31 min; *m*/*z*: calcd for C_16_H_20_N_3_O_4_^+^ [M + H]^+^, 318.1448; found, 318.1441.

##### Step 2: 3-Bromo-4-((1-(1-(hydroxymethyl)cyclopropyl)ethyl)amino)-1-methyl-6-nitroquinolin-2(1*H*)-one (**27n**)

Prepared from **26n** using the procedure described for **27a**. **27n** (92 mg, 70%) was obtained as a yellow solid. ^1^H NMR (500 MHz, CDCl_3_): δ 8.82 (d, *J* = 2.5 Hz, 1 H), 8.41 (dd, *J* = 9.4, 2.5 Hz, 1 H), 7.72 (d, *J* = 9.4 Hz, 1 H), 6.59 (d, *J* = 9.1 Hz, 1 H), 5.36 (br s, 1 H), 4.04 (d, *J* = 11.4 Hz, 1 H), 3.75–3.71 (m, 1 H), 3.68 (s, 3 H), 2.97 (d, *J* = 11.4 Hz, 1 H), 1.36 (d, *J* = 6.7 Hz, 3 H), 0.58–0.53 (m, 1 H), 0.53–0.49 (m, 1 H), 0.49–0.45 (m, 1 H), 0.38–0.33 (m, 1 H); LCMS (2 min; ToF); RT 1.42 min; *m*/*z*: calcd for C_16_H_19_BrN_3_O_4_^+^ [M + H]^+^, 396.0553; found, 396.0542.

##### Step 3: 2′,7′-Dimethyl-10′-nitro-1′,2′-dihydro-4′H-spiro[cyclopropane-1,3′-[1,4]oxazepino[2,3-*c*]quinolin]-6′(7′*H*)-one (**28n**)

Prepared from **27n** using the procedure described for **28a**. A modified reaction time of 90 min was used. **28n** (13 mg, 18%) was obtained as an orange solid. ^1^H NMR (500 MHz, CDCl3): δ 8.98 (d, *J* = 2.5 Hz, 1 H), 8.32 (dd, *J* = 9.4, 2.5 Hz, 1 H), 7.61 (d, *J* = 9.4 Hz, 1 H), 6.80 (d, *J* = 5.4 Hz, 1 H), 4.22 (d, *J* = 11.4 Hz, 1 H), 3.61 (s, 3 H), 3.57 (d, *J* = 11.4 Hz, 1 H), 3.29–3.24 (m, 1 H), 1.36 (d, *J* = 6.8 Hz, 3 H), 0.59–0.54 (m, 1 H), 0.50–0.45 (m, 1 H), 0.43–0.38 (m, 1 H), 0.35–0.30 (m, 1 H); LCMS (2 min; ToF); RT 1.32 min; *m*/*z*: calcd for C_16_H_18_N_3_O_4_^+^ [M + H]^+^, 316.1292; found, 316.1292.

##### Step 4: 10′-Amino-2′,7′-dimethyl-1′,2′-dihydro-4′*H*-spiro[cyclopropane-1,3′-[1,4]oxazepino[2,3-*c*]quinolin]-6′(7′*H*)-one (**29n**)

Prepared from **28n** using the procedure described for **29a**. **29n** (12 mg, 99%) was obtained as a yellow solid. LCMS (2 min; ToF) RT 0.74 min; *m*/*z*: calcd for C_16_H_20_N_3_O_2_^+^ [M + H]^+^, 286.1550; found, 286.1555.

##### Step 5: 2-Chloro-4-((2′,7′-dimethyl-6′-oxo-1′,2′,6′,7′-tetrahydro-4′*H*-spiro[cyclopropane-1,3′-[1,4]oxazepino[2,3-*c*]quinolin]-10′-yl)amino)nicotinonitrile (**13d**)

Prepared from **29n** using the procedure described for **9a**. **13d** (4 mg, 46%) was obtained as an off-white solid. ^1^H NMR (600 MHz, CD_3_OD): δ 7.98 (d, *J* = 6.2 Hz, 1 H), 7.90 (d, *J* = 2.3 Hz, 1 H), 7.62 (d, *J* = 9.0 Hz, 1 H), 7.51 (dd, *J* = 9.0, 2.3 Hz, 1 H), 6.70 (d, *J* = 6.2 Hz, 1 H), 4.35 (d, *J* = 11.4 Hz, 1 H), 3.74 (s, 3 H), 3.67 (d, *J* = 11.4 Hz, 1 H), 3.32–3.30 (m, 1 H), 1.44 (d, *J* = 6.8 Hz, 3 H), 0.60–0.55 (m, 2 H), 0.49–0.45 (m, 1 H), 0.39–0.36 (m, 1 H); LCMS (4 min; ToF) RT 2.63 min; *m*/*z*: calcd for C_22_H_21_ClN_5_O_2_^+^ [M + H]^+^, 422.1378; found, 422.1356.

#### (*R*)-2-Chloro-4-((2-cyclopropyl-3,3-difluoro-7-methyl-6-oxo-1,2,3,4,6,7-hexahydro-[1,4]oxazepino[2,3-*c*]quinolin-10-yl)amino)nicotinonitrile (**14**)

Prepared as for **1** starting from **34b**. The product was further purified by column chromatography (0–5% methanol in DCM) to give the title compound (1.4 mg, 7%). ^1^H NMR (600 MHz, DMSO-*d*_6_): δ 9.62 (s, 1 H), 8.10 (d, *J* = 2.3 Hz, 1 H), 8.06 (d, *J* = 6.1 Hz, 1 H), 7.54 (d, *J* = 8.9 Hz, 1 H), 7.48 (dd, *J* = 8.9, 2.3 Hz, 1 H), 6.64 (d, *J* = 6.2 Hz, 1 H), 6.39–6.33 (m, 1 H), 4.54–4.33 (m, 2 H), 3.59 (s, 3 H), 3.27–3.18 (m, 1 H), 1.34–1.24 (m, 1 H), 0.78–0.69 (m, 1 H), 0.55–0.45 (m, 2 H), 0.36–0.26 (m, 1 H); LCMS (4 min; ToF) RT 2.51 min; *m*/*z*: calcd for C_22_H_19_ClF_2_N_5_O_2_^+^ [M + H]^+^, 458.1190; found, 458.1190.

#### (*S*)-4-((1-Cyclopropyl-2,2-difluoro-3-hydroxypropyl)amino)-1-methyl-6-nitroquinolin-2(1*H*)-one (**32**)

To a mixture of (*S*)-3-amino-3-cyclopropyl-2,2-difluoropropan-1-ol hydrochloride **31** [1.99 g, 10.6 mmol (supplied as ∼90% ee by SIA Enamine, Latvia)] and **20b** (3.0 g, 9.7 mmol) under argon was added anhydrous acetonitrile (15 mL), followed by DIPEA (4.2 mL, 24.1 mmol). The reaction mixture was heated at 160 °C under microwave irradiation for 15 h. 2 M sodium hydroxide (30 mL, 60 mmol) was added, and the reaction mixture was heated at 85 °C for 2 h. The reaction mixture was cooled to rt. Water (80 mL) was added, and the reaction mixture was acidified to pH 4–5 with 3 M HCl. The resulting precipitate was filtered, washed with water (200 mL), and dried under vacuum, affording **32** (3.18 g, 93%) as a beige solid. ^1^H NMR (500 MHz, DMSO-*d*_6_): δ 9.31 (d, *J* = 2.5 Hz, 1 H), 8.40 (dd, *J* = 9.4, 2.5 Hz, 1 H), 7.62 (d, *J* = 9.4 Hz, 1 H), 7.48 (d, *J* = 8.7 Hz, 1 H), 5.72 (s, 1 H), 5.60 (t, *J* = 6.1 Hz, 1 H), 3.90–3.71 (m, 2 H), 3.57–3.45 (m, 4 H), 1.38–1.29 (m, 1 H), 0.71–0.64 (m, 1 H), 0.63–0.56 (m, 1 H), 0.53–0.46 (m, 1 H), 0.27–0.20 (m, 1 H); LCMS (2 min; ESI) RT 1.15 min; *m*/*z*: calcd for C_16_H_18_F_2_N_3_O_4_^+^ [M + H]^+^, 354.1260; found, 354.1270.

#### (*S*)-3-Bromo-4-((1-cyclopropyl-2,2-difluoro-3-hydroxypropyl)amino)-1-methyl-6-nitroquinolin-2(1*H*)-one (**33**)

Trifluoroacetic acid (3.44 mL, 44.9 mmol) was added to a stirred mixture of **32** (3.18 g, 8.99 mmol) and freshly recrystallized *N*-bromosuccinimide (1.60 g, 8.98 mmol) in anhydrous DCM (60 mL) at 0 °C under Ar. The reaction mixture was stirred at 0 °C for 10 min. The reaction mixture was diluted with DCM (100 mL) and washed with saturated aq. NaHCO_3_ (3 × 80 mL). The aqueous washings were further extracted with DCM (100 mL). The organic extracts were combined, washed with brine (80 mL), dried (Na_2_SO_4_), and concentrated under reduced pressure, affording **33** (3.77 g, 97%) as a yellow solid. ^1^H NMR (500 MHz, DMSO-*d*_6_): δ 8.95 (d, *J* = 2.5 Hz, 1 H), 8.43 (dd, *J* = 9.4, 2.5 Hz, 1 H), 7.75 (d, *J* = 9.4 Hz, 1 H), 5.86 (d, *J* = 11.1 Hz, 1 H), 5.63 (t, *J* = 5.9 Hz, 1 H), 4.05–3.95 (m, 1 H), 3.89–3.74 (m, 2 H), 3.71 (s, 3 H), 1.29–1.21 (m, 1 H), 0.68–0.62 (m, 1 H), 0.62–0.51 (m, 2 H), 0.50–0.44 (m, 1 H); LCMS (2 min; ESI) RT 1.31 min; *m*/*z*: calcd for C_16_H_17_BrF_2_N_3_O_4_^+^ [M + H]^+^, 432.0365; found, 432.0369.

#### (*S*)-2-Cyclopropyl-3,3-difluoro-7-methyl-10-nitro-1,2,3,4-tetrahydro-[1,4]oxazepino-[2,3-*c*]quinolin-6(7*H*)-one (**34a**) and (*R*)-2-Cyclopropyl-3,3-difluoro-7-methyl-10-nitro-1,2,3,4-tetrahydro-[1,4]oxazepino-[2,3-*c*]quinolin-6(7*H*)-one (**34b**)

Lithium *tert*-butoxide (1 M in THF; 13.9 mL, 13.9 mmol) was added to a suspension of **33** (3.77 g, 8.71 mmol) in THF (87 mL) under Ar. The reaction mixture was heated at 60 °C for 15 min and then cooled to rt. Water (100 mL) was added, and the aqueous mixture was extracted with DCM (3 × 100 mL). The organic extracts were combined, washed with brine (2 × 100 mL), dried (Na_2_SO_4_), and concentrated under reduced pressure, affording **34a** (3.01 g, 98%) as a yellow solid. ^1^H NMR (500 MHz, DMSO-*d*_6_): δ 9.12 (d, *J* = 2.5 Hz, 1 H), 8.35 (dd, *J* = 9.4, 2.5 Hz, 1 H), 7.66 (d, *J* = 9.4 Hz, 1 H), 7.01 (d, *J* = 4.4 Hz, 1 H), 4.54–4.37 (m, 2 H), 3.62 (s, 3 H, NCH_3_), 3.29–3.22 (m, 1 H), 1.39–1.31 (m, 1 H), 0.76–0.69 (m, 1 H), 0.58–0.49 (m, 2 H), 0.37–0.30 (m, 1 H); LCMS (2 min; ESI) RT 1.29 min; *m*/*z*: calcd for C_16_H_16_F_2_N_3_O_4_^+^ [M + H]^+^, 352.1103; found, 352.1105. Due to the presence of the minor enantiomer in starting material **31**, a solution of this product (est. 90% ee) at 25 mg/mL in DCM/methanol (9:1) was purified by SFC by Reach Separations, Nottingham, using Chiralpak SA, 30:70% methanol/CO_2_, 0.2% v/v NH_3_. Combined fractions of the major product were dissolved in DCM and heptane, evaporated, and dried in a vacuum oven to give **34a** as a yellow solid (1.6 g, 67%). Chiral purity was assessed by SFC (YMA Amylose-C, 30:70 MeOH/CO_2_; 0.2% v/v NH_3_), which showed 99% ee. Fractions containing the minor isomer **34b** were repurified by the same method, affording this compound as an orange solid (143 mg, 6%). Chiral purity was assessed by SFC (as above), which showed 98% ee. Compounds **34a** and **34b** were used without further purification in the preparation of **1** and **14**.

## Supporting Information Available

Molecular formula strings with associated biochemical assay data and calculated properties (CSV)
Experimental methods; protein production, purification, and crystallography (BCL6 constructs used, methods for expression, purification, and crystallography, including data collection, processing, and refinement); biological assay conditions (methods for TR-FRET, NanoBRET, and cell proliferation assays); physicochemical assay conditions (methods for NMR and HPLC solubility assays); synthetic and analytical methods (general information on synthesis and purification, NMR, and LCMS analysis); analytical data; HPLC/MS traces for key compounds; chiral SFC traces; summary statistics and individual replicate values; crystallographic data collection and refinement statistics; and conditions used in the optimization of the cyclization reaction to form **19** (PDF)
PDB 7Q7R validation report (PDF)
PDB 7Q7S validation report (PDF)
PDB 7Q7T validation report (PDF)
PDB 7Q7U validation report (PDF)
PDB 7Q7V validation report (PDF)

## Accession Codes

Atomic coordinates and structure factors for the crystal structures of BCL6 with compounds **1**, **4**, **7, 9a**, and **12a** can be accessed using PDB codes 7Q7R, 7Q7S, 7Q7T, 7Q7U, and 7Q7V, respectively. The authors will release the atomic coordinates and experimental data upon article publication.

## Abbreviations

BCL6: B-cell lymphoma 6
BCOR: BCL6 corepressor
BTB [domain]: broad-complex tramtrack and bric a brac, also known as a POZ domain
DCE: 1,2-dichloroethane
DIPEA: *N*,*N*-diisopropylethylamine
DLBCL: diffuse large B-cell lymphoma
GC: germinal center
HDCH site: region of the BCL6 binding pocket defined in ref (15)
LE: ligand efficiency
LLE: lipophilic ligand efficiency
NanoBRET: a BRET-based assay format commercialized by Promega that uses NanoLuc luciferase to generate the donor signal and a HaloTag ligand as the fluorescent energy acceptor
NBS: *N*-bromosuccinimide
NCOR: nuclear receptor corepressor
PAMPA: parallel artificial membrane permeability assay
rt: room temperature (approx. 20 °C)
RT: retention time
TEA: triethylamine
TPSA: topological polar surface area
TR-FRET: time-resolved fluorescence energy transfer
