# Supplementary material for: Optimizing Shape Complementarity Enables the Discovery of Potent Tricyclic BCL6 Inhibitors
Source: J Med Chem. 2022 Jun 3;65(12):8169–90. doi: 10.1021/acs.jmedchem.1c02174 (PMC9234963; doi:10.1021/acs.jmedchem.1c02174)
Supplement: Supplementary file 3 — jm1c02174_si_003.pdf [file jm1c02174_si_003.pdf]

Full wwPDB X-ray Structure Validation Report (i)

Nov 11, 2021 – 03:41 pm GMT

PDB ID	:	7Q7S
Title	:	Crystal structure of human BCL6 BTB domain in complex with compound 4
Deposited on	:	2021-11-09
Resolution	:	1.44 Å(reported)

This is a Full wwPDB X-ray Structure Validation Report.

This report is produced by the wwPDB biocuration pipeline after annotation of the structure.

We welcome your comments at *validation@mail.wwpdb.org*
A user guide is available at
https://www.wwpdb.org/validation/2017/XrayValidationReportHelp
with specific help available everywhere you see the (i) symbol.

CONFIDENTIAL VALIDATION REPORT

The following versions of software and data (see references (i)) were used in the production of this report:

MolProbity	:	4.02b-467
Mogul	:	1.8.4 (270009), CSD as541be (2020)
Xtriage (Phenix)	:	1.13
EDS	:	2.23.2
buster-report	:	1.1.7 (2018)
Percentile statistics	:	20191225.v01 (using entries in the PDB archive December 25th 2019)
Refmac	:	5.8.0267
CCP4	:	7.1.010 (Gargrove)
Ideal geometry (proteins)	:	Engh & Huber (2001)
Ideal geometry (DNA, RNA)	:	Parkinson et al. (1996)
Validation Pipeline (wwPDB-VP)	:	2.23.2

1 Overall quality at a glance (i)

The following experimental techniques were used to determine the structure:
X-RAY DIFFRACTION

The reported resolution of this entry is 1.44 Å.

Percentile scores (ranging between 0-100) for global validation metrics of the entry are shown in the following graphic. The table shows the number of entries on which the scores are based.

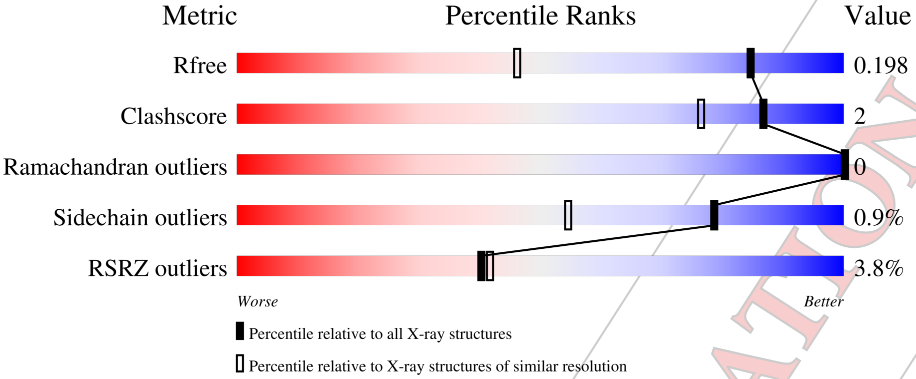

Metric	Whole archive (#Entries)	Similar resolution (#Entries, resolution range(Å))
R_{free}	130704	2021 (1.46-1.42)
Clashscore	141614	2086 (1.46-1.42)
Ramachandran outliers	138981	2047 (1.46-1.42)
Sidechain outliers	138945	2047 (1.46-1.42)
RSRZ outliers	127900	1993 (1.46-1.42)

The table below summarises the geometric issues observed across the polymeric chains and their fit to the electron density. The red, orange, yellow and green segments of the lower bar indicate the fraction of residues that contain outliers for >=3, 2, 1 and 0 types of geometric quality criteria respectively. A grey segment represents the fraction of residues that are not modelled. The numeric value for each fraction is indicated below the corresponding segment, with a dot representing fractions <=5% The upper red bar (where present) indicates the fraction of residues that have poor fit to the electron density. The numeric value is given above the bar.

Mol	Chain	Length	Quality of chain
1	A	128	4%<br>92% 5% •
2	B	6	100%

CONFIDENTIAL VALIDATION REPORT

2 Entry composition (i)

There are 6 unique types of molecules in this entry. The entry contains 1401 atoms, of which 0 are hydrogens and 0 are deuteriums.

In the tables below, the ZeroOcc column contains the number of atoms modelled with zero occupancy, the AltConf column contains the number of residues with at least one atom in alternate conformation and the Trace column contains the number of residues modelled with at most 2 atoms.

- Molecule 1 is a protein called B-cell lymphoma 6 protein.

Mol	Chain	Residues	Atoms					ZeroOcc	AltConf	Trace
			Total	C	N	O	S			
1	A	125	1102	692	191	207	12	0	15	0

There are 3 discrepancies between the modelled and reference sequences:

Chain	Residue	Modelled	Actual	Comment	Reference
A	2	GLY	-	expression tag	UNP P41182
A	3	PRO	-	expression tag	UNP P41182
A	4	GLY	-	expression tag	UNP P41182

- Molecule 2 is a protein called ALA-TRP-VAL-ILE-PRO-ALA.

Mol	Chain	Residues	Atoms				ZeroOcc	AltConf	Trace
			Total	C	N	O			
2	B	6	40	29	6	5	0	0	1

- Molecule 3 is 2-chloranyl-4-[[4-(ethylamino)-1,3-dimethyl-2-oxidanylidene-quinolin-6-yl]amino]pyridine-3-carbonitrile (three-letter code: 97S) (formula: $C_{19}H_{18}ClN_5O$) (labeled as "Ligand of Interest" by depositor).

CONFIDENTIAL VALIDATION REPORT

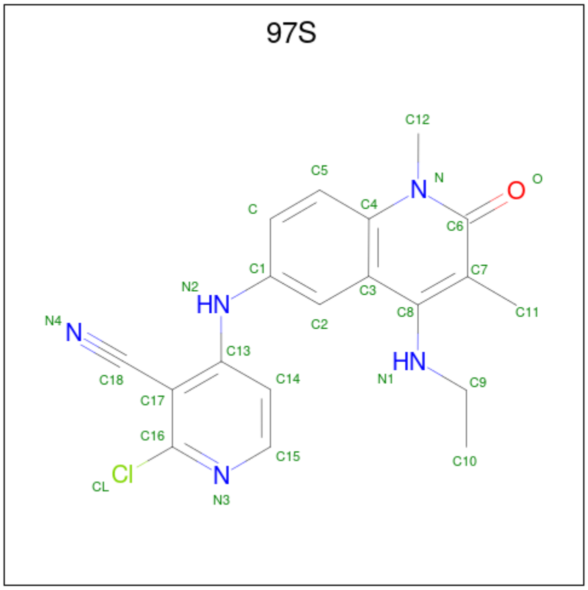

Mol	Chain	Residues	Atoms					ZeroOcc	AltConf
			Total	C	Cl	N	O		
3	A	1	26	19	1	5	1	0	0

- Molecule 4 is 1,2-ETHANEDIOL (three-letter code: EDO) (formula: $C_2H_6O_2$).

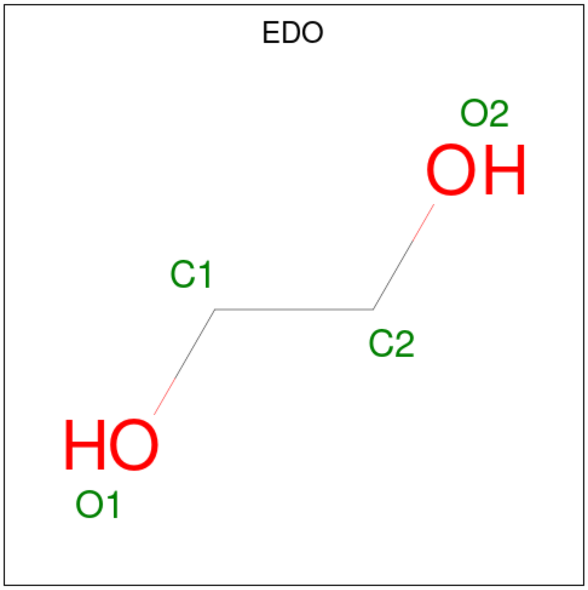

Mol	Chain	Residues	Atoms			ZeroOcc	AltConf
			Total	C	O		
4	A	1	4	2	2	0	0
4	A	1	4	2	2	0	0
4	A	1	4	2	2	0	0

Continued on next page...

CONFIDENTIAL VALIDATION REPORT

Continued from previous page...

Mol	Chain	Residues	Atoms	ZeroOcc	AltConf
4	A	1	Total C O<br>4 2 2	0	0
4	A	1	Total C O<br>4 2 2	0	0

- Molecule 5 is CHLORIDE ION (three-letter code: CL) (formula: Cl).

Mol	Chain	Residues	Atoms	ZeroOcc	AltConf
5	A	2	Total Cl<br>2 2	0	1

- Molecule 6 is water.

Mol	Chain	Residues	Atoms	ZeroOcc	AltConf
6	A	200	Total O<br>204 204	0	7
6	B	7	Total O<br>7 7	0	0

CONFIDENTIAL VALIDATION REPORT

3 Residue-property plots (i)

These plots are drawn for all protein, RNA, DNA and oligosaccharide chains in the entry. The first graphic for a chain summarises the proportions of the various outlier classes displayed in the second graphic. The second graphic shows the sequence view annotated by issues in geometry and electron density. Residues are color-coded according to the number of geometric quality criteria for which they contain at least one outlier: green = 0, yellow = 1, orange = 2 and red = 3 or more. A red dot above a residue indicates a poor fit to the electron density (RSRZ > 2). Stretches of 2 or more consecutive residues without any outlier are shown as a green connector. Residues present in the sample, but not in the model, are shown in grey.

- Molecule 1: B-cell lymphoma 6 protein

Chain A:
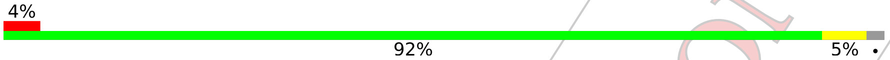

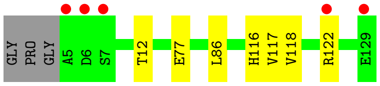

- Molecule 2: ALA-TRP-VAL-ILE-PRO-ALA

Chain B:
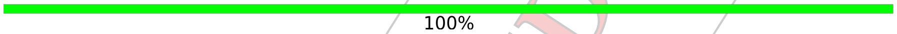

There are no outlier residues recorded for this chain.

CONFIDENTIAL VALIDATION REPORT

4 Data and refinement statistics (i)

Property	Value	Source
Space group	P 61 2 2	Depositor
Cell constants<br>a, b, c, α, β, γ	67.20Å 67.20Å 165.14Å<br>90.00° 90.00° 120.00°	Depositor
Resolution (Å)	47.57 – 1.44<br>47.57 – 1.44	Depositor<br>EDS
% Data completeness<br>(in resolution range)	99.5 (47.57-1.44)<br>99.5 (47.57-1.44)	Depositor<br>EDS
R_{merge}	0.12	Depositor
R_{sym}	(Not available)	Depositor
$< I/\sigma(I) >$ [1]	1.43 (at 1.44Å)	Xtriage
Refinement program	BUSTER 2.10.4 (24-FEB-2021)	Depositor
R, R_{free}	0.184 , 0.200<br>0.178 , 0.198	Depositor<br>DCC
R_{free} test set	1997 reflections (4.91%)	wwPDB-VP
Wilson B-factor (Å^2)	15.4	Xtriage
Anisotropy	0.345	Xtriage
Bulk solvent k_{sol}(e/Å^3), B_{sol}(Å^2)	(Not available) , (Not available)	EDS
L-test for twinning[2]	$< \|L\| > = 0.48$, $< L^2 > = 0.31$	Xtriage
Estimated twinning fraction	No twinning to report.	Xtriage
F_o,F_c correlation	0.97	EDS
Total number of atoms	1401	wwPDB-VP
Average B, all atoms (Å^2)	23.0	wwPDB-VP

Xtriage's analysis on translational NCS is as follows: *The largest off-origin peak in the Patterson function is 7.28% of the height of the origin peak. No significant pseudotranslation is detected.*

[1] Intensities estimated from amplitudes.

[2] Theoretical values of $< |L| >$, $< L^2 >$ for acentric reflections are 0.5, 0.333 respectively for untwinned datasets, and 0.375, 0.2 for perfectly twinned datasets.

CONFIDENTIAL VALIDATION REPORT

5 Model quality (i)

5.1 Standard geometry (i)

Bond lengths and bond angles in the following residue types are not validated in this section: EDO, 97S, CL

The Z score for a bond length (or angle) is the number of standard deviations the observed value is removed from the expected value. A bond length (or angle) with $|Z| > 5$ is considered an outlier worth inspection. RMSZ is the root-mean-square of all Z scores of the bond lengths (or angles).

Mol	Chain	Bond lengths		Bond angles	
		RMSZ	$\#\|Z\| > 5$	RMSZ	$\#\|Z\| > 5$
1	A	0.44	0/1123	0.58	0/1514
2	B	0.40	0/42	0.58	0/59
All	All	0.44	0/1165	0.58	0/1573

There are no bond length outliers.

There are no bond angle outliers.

There are no chirality outliers.

There are no planarity outliers.

5.2 Too-close contacts (i)

In the following table, the Non-H and H(model) columns list the number of non-hydrogen atoms and hydrogen atoms in the chain respectively. The H(added) column lists the number of hydrogen atoms added and optimized by MolProbity. The Clashes column lists the number of clashes within the asymmetric unit, whereas Symm-Clashes lists symmetry-related clashes.

Mol	Chain	Non-H	H(model)	H(added)	Clashes	Symm-Clashes
1	A	1102	0	1088	5	0
2	B	40	0	37	0	0
3	A	26	0	0	0	0
4	A	20	0	30	0	0
5	A	2	0	0	0	0
6	A	204	0	0	2	0
6	B	7	0	0	0	0
All	All	1401	0	1155	5	0

The all-atom clashscore is defined as the number of clashes found per 1000 atoms (including hydrogen atoms). The all-atom clashscore for this structure is 2.

CONFIDENTIAL VALIDATION REPORT

All (5) close contacts within the same asymmetric unit are listed below, sorted by their clash magnitude.

Atom-1	Atom-2	Interatomic distance (Å)	Clash overlap (Å)
1:A:12[B]:THR:HG22	6:A:329:HOH:O	1.87	0.74
1:A:86:LEU:HD21	1:A:117:VAL:HG11	1.93	0.51
1:A:116[B]:HIS:N	1:A:116[B]:HIS:ND1	2.62	0.47
1:A:12[B]:THR:HG21	6:A:389:HOH:O	2.18	0.43
1:A:118:VAL:HG12	1:A:122[B]:ARG:HE	1.84	0.43

There are no symmetry-related clashes.

5.3 Torsion angles (i)

5.3.1 Protein backbone (i)

In the following table, the Percentiles column shows the percent Ramachandran outliers of the chain as a percentile score with respect to all X-ray entries followed by that with respect to entries of similar resolution.

The Analysed column shows the number of residues for which the backbone conformation was analysed, and the total number of residues.

Mol	Chain	Analysed	Favoured	Allowed	Outliers	Percentiles	
1	A	140/128 (109%)	138 (99%)	2 (1%)	0	100	100
2	B	4/6 (67%)	4 (100%)	0	0	100	100
All	All	144/134 (108%)	142 (99%)	2 (1%)	0	100	100

There are no Ramachandran outliers to report.

5.3.2 Protein sidechains (i)

In the following table, the Percentiles column shows the percent sidechain outliers of the chain as a percentile score with respect to all X-ray entries followed by that with respect to entries of similar resolution.

The Analysed column shows the number of residues for which the sidechain conformation was analysed, and the total number of residues.

Mol	Chain	Analysed	Rotameric	Outliers	Percentiles	
1	A	126/115 (110%)	124 (98%)	2 (2%)	62	31

Continued on next page...

CONFIDENTIAL VALIDATION REPORT

Continued from previous page...

Mol	Chain	Analysed	Rotameric	Outliers	Percentiles	
2	B	4/4 (100%)	4 (100%)	0	100	100
All	All	130/119 (109%)	128 (98%)	2 (2%)	78	34

All (2) residues with a non-rotameric sidechain are listed below:

Mol	Chain	Res	Type
1	A	77[A]	GLU
1	A	77[B]	GLU

Sometimes sidechains can be flipped to improve hydrogen bonding and reduce clashes. There are no such sidechains identified.

5.3.3 RNA (i)

There are no RNA molecules in this entry.

5.4 Non-standard residues in protein, DNA, RNA chains (i)

There are no non-standard protein/DNA/RNA residues in this entry.

5.5 Carbohydrates (i)

There are no monosaccharides in this entry.

5.6 Ligand geometry (i)

Of 8 ligands modelled in this entry, 2 are monoatomic - leaving 6 for Mogul analysis.

There are no bond length outliers.

There are no bond angle outliers.

There are no chirality outliers.

There are no torsion outliers.

There are no ring outliers.

No monomer is involved in short contacts.

The following is a two-dimensional graphical depiction of Mogul quality analysis of bond lengths, bond angles, torsion angles, and ring geometry for all instances of the Ligand of Interest. In addition, ligands with molecular weight > 250 and outliers as shown on the validation Tables will

CONFIDENTIAL VALIDATION REPORT

also be included. For torsion angles, if less then 5% of the Mogul distribution of torsion angles is within 10 degrees of the torsion angle in question, then that torsion angle is considered an outlier. Any bond that is central to one or more torsion angles identified as an outlier by Mogul will be highlighted in the graph. For rings, the root-mean-square deviation (RMSD) between the ring in question and similar rings identified by Mogul is calculated over all ring torsion angles. If the average RMSD is greater than 60 degrees and the minimal RMSD between the ring in question and any Mogul-identified rings is also greater than 60 degrees, then that ring is considered an outlier. The outliers are highlighted in purple. The color gray indicates Mogul did not find sufficient equivalents in the CSD to analyse the geometry.

Ligand 97S A 201

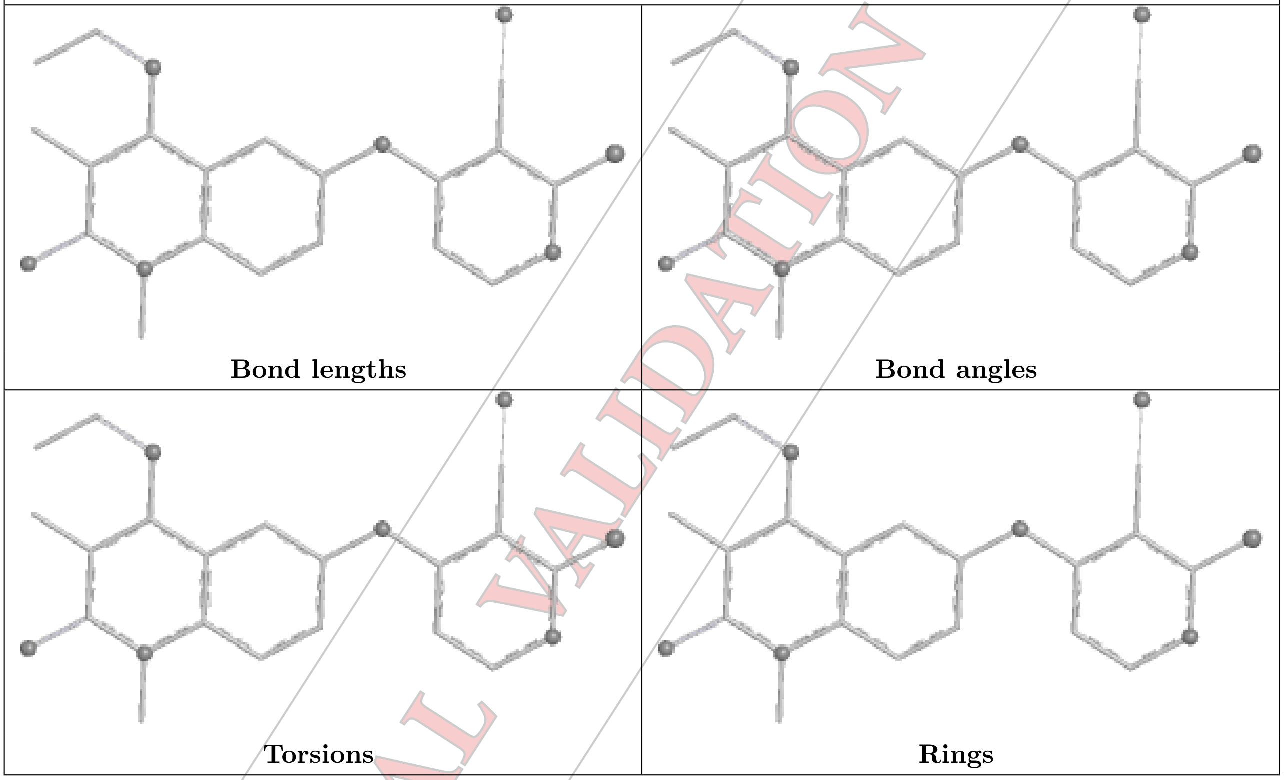

5.7 Other polymers (i)

There are no such residues in this entry.

5.8 Polymer linkage issues (i)

There are no chain breaks in this entry.

CONFIDENTIAL VALIDATION REPORT

6 Fit of model and data (i)

6.1 Protein, DNA and RNA chains (i)

In the following table, the column labelled '#RSRZ> 2' contains the number (and percentage) of RSRZ outliers, followed by percent RSRZ outliers for the chain as percentile scores relative to all X-ray entries and entries of similar resolution. The OWAB column contains the minimum, median, 95th percentile and maximum values of the occupancy-weighted average B-factor per residue. The column labelled 'Q< 0.9' lists the number of (and percentage) of residues with an average occupancy less than 0.9.

Mol	Chain	Analysed	<RSRZ>	#RSRZ>2			OWAB(Å^2)	Q<0.9
1	A	125/128 (97%)	0.40	5 (4%)	38	39	12, 17, 30, 49	2 (1%)
2	B	6/6 (100%)	0.15	0	100	100	14, 17, 18, 20	0
All	All	131/134 (97%)	0.39	5 (3%)	40	42	12, 17, 30, 49	2 (1%)

All (5) RSRZ outliers are listed below:

Mol	Chain	Res	Type	RSRZ
1	A	6	ASP	6.3
1	A	5	ALA	5.3
1	A	129	GLU	3.1
1	A	122[A]	ARG	2.6
1	A	7	SER	2.4

6.2 Non-standard residues in protein, DNA, RNA chains (i)

There are no non-standard protein/DNA/RNA residues in this entry.

6.3 Carbohydrates (i)

There are no monosaccharides in this entry.

6.4 Ligands (i)

In the following table, the Atoms column lists the number of modelled atoms in the group and the number defined in the chemical component dictionary. The B-factors column lists the minimum, median, 95th percentile and maximum values of B factors of atoms in the group. The column labelled 'Q< 0.9' lists the number of atoms with occupancy less than 0.9.

CONFIDENTIAL VALIDATION REPORT

Mol	Type	Chain	Res	Atoms	RSCC	RSR	B-factors(Å^2)	Q<0.9
4	EDO	A	204	4/4	0.70	0.15	73,73,73,73	0
4	EDO	A	203	4/4	0.74	0.20	44,44,44,44	4
4	EDO	A	206	4/4	0.75	0.17	65,65,65,65	0
4	EDO	A	205	4/4	0.86	0.17	23,24,25,25	4
4	EDO	A	202	4/4	0.89	0.15	24,25,26,28	4
3	97S	A	201	26/26	0.92	0.12	14,17,22,25	26
5	CL	A	208	1/1	0.96	0.09	48,48,48,48	1
5	CL	A	207[A]	1/1	0.98	0.09	23,23,23,23	1

The following is a graphical depiction of the model fit to experimental electron density of all instances of the Ligand of Interest. In addition, ligands with molecular weight > 250 and outliers as shown on the geometry validation Tables will also be included. Each fit is shown from different orientation to approximate a three-dimensional view.

Electron density around 97S A 201:

2mF$_o$-DF$_c$ (at 0.7 rmsd) in gray
mF$_o$-DF$_c$ (at 3 rmsd) in purple (negative)
and green (positive)

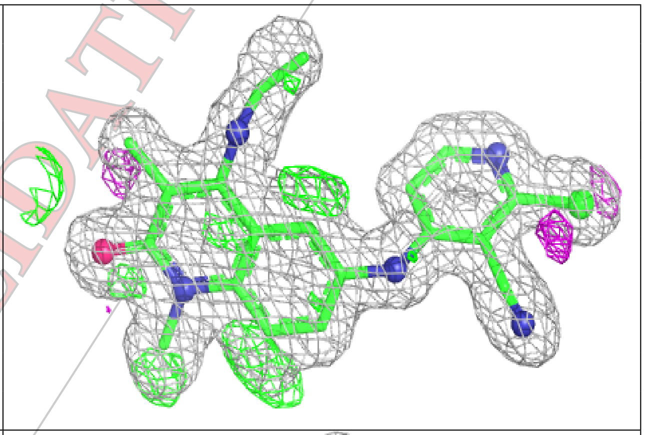

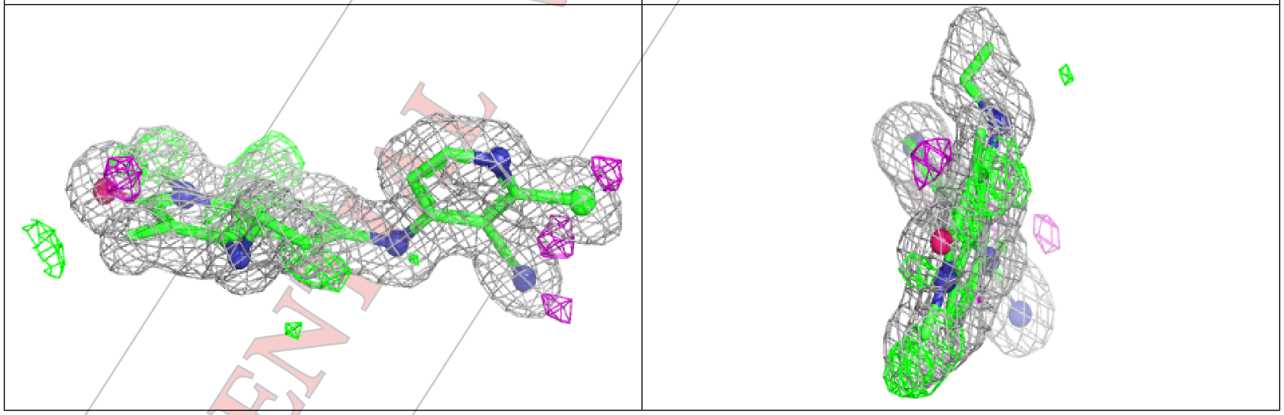

6.5 Other polymers ⓘ

There are no such residues in this entry.

CONFIDENTIAL VALIDATION REPORT
