# Supplementary material for: Optimizing Shape Complementarity Enables the Discovery of Potent Tricyclic BCL6 Inhibitors
Source: J Med Chem. 2022 Jun 3;65(12):8169–90. doi: 10.1021/acs.jmedchem.1c02174 (PMC9234963; doi:10.1021/acs.jmedchem.1c02174)
Supplement: Supplementary file 7 — jm1c02174_si_007.pdf [file jm1c02174_si_007.pdf]

Supporting information

Optimising shape complementarity enables the discovery of potent tricyclic BCL6 inhibitors

Owen A. Davis,[†] Kwai-Ming J. Cheung,[†] Alfie Brennan,[†] Matthew G. Lloyd,[†] Matthew J. Rodrigues,[†,#] Olivier A. Pierrat,[†] Gavin W. Collie,[†,#] Yann-Vaï Le Bihan,[†,#] Rosemary Huckvale,[†] Alice C. Harnden,[†] Ana Varela,[†] Michael D. Bright,[†] Paul Eve,[†] Angela Hayes,[†] Alan T. Henley,[†] Michael D. Carter,[†] P. Craig McAndrew,[†] Rachel Talbot,[†] Rosemary Burke,[†] Rob L. M. van Montfort,[†,#] Florence I. Raynaud,[†] Olivia W. Rossanese,[†] Mirco Meniconi,[†] Benjamin R. Bellenie,[†,*] and Swen Hoelder.[†,*]

[†]Cancer Research UK Cancer Therapeutics Unit and [#]Division of Structural Biology, The Institute of Cancer Research, London SM2 5NG, U.K.

* email addresses benjamin.bellenie@icr.ac.uk; swen.hoelder@icr.ac.uk

Contents

1. Supplementary experimental: protein production, purification and crystallography......S-3

1.1 BCL6 constructs used for assays and crystallography S-3

1.2 BCL6 expression......S-3

1.3 BCL6 purification......S-3

1.4 BCL6 crystallisation S-4

1.5 Crystallographic data collection, processing and refinement......S-4

2. Supplementary experimental: biological assay conditions......S-6

2.1 TR-FRET assay......S-6

2.2 NanoBRET assay S-6

2.3 Cell proliferation assay......S-7

3. Supplementary experimental: physicochemical assays S-8

3.1 NMR solubility assay S-8

3.2 HPLC solubility assay S-9

4. Supplementary information: synthetic and analytical methods......S-9

4.1 General Synthetic Information S-9

4.2 Analytical Methods......S-9

5. Supplementary analytical data......S-11

5.1 HPLC/MS traces for key compounds S-11

5.1.1 Compound 1 ......S-11

5.1.2 Compound 7 ......S-12

5.1.3 Compound 9a......S-13

5.1.4 Compound 12a S-14

5.2 Chiral SFC traces S-15

5.2.1 Compound 34a S-15

5.2.2 **Compound 34b** S-16

6. Supplementary tables S-17

Table S1: summary statistics and individual replicate values S-17

*Table S2: Crystallographic data collection and refinement statistics for **4**, **7** and **9a**.* S-20

*Table S3: Crystallographic data collection and refinement statistics for **12a** and **1**.* S-21

Table S4: Optimisation of the cyclisation reaction forming 19 ....S-22

7. References for supplementary information S-22

1. Supplementary experimental: protein production, purification and crystallography

1.1 BCL6 constructs used for assays and crystallography

A first construct of BCL6 BTB domain, which we will refer to as Trx-6His-HRV3C-BCL6, was obtained by sub-cloning the sequence coding for residues 5-129 of human BCL6, corresponding to its BTB domain, into a pET48b vector with N-terminal Thioredoxin and 6-Histidine tags, followed by a HRV-3C protease cleavage site. For the TR-FRET assay, the Trx-6His-HRV3C-BCL6 protein construct was used without cleaving the tag, as a 6His was needed to bind to the anti-6His-Terbium antibody. For crystallography with compounds **4** and **7**, the tag was removed by HRV-3C protease treatment, generating a simpler BCL6 5-129 product.

For crystallography with compounds **9a**, **12a** and **1**, the construct described above was modified to introduce a Flag Tag and a TEV cleavage site between the HRV3C and BCL6 sequences. This construct will be referred to as Flag-TEV-BCL6.

1.2 BCL6 expression

For both plasmid constructs described above, transformed BL21-AI E. coli cells were grown in LB media supplemented with 50 mg/L kanamycin at 37 ^{o}C until an OD600 nm of 0.6 was reached. Protein expression was then induced by addition of 0.2 mM IPTG and 0.2 % Arabinose. Expression was carried out at 18 ^{o}C for 18 hours. Cells were harvested by centrifugation (5500 g for 30 minutes at 4 ^{o}C) and stored at -80 ^{o}C.

1.3 BCL6 purification

Cells were re-suspended in a buffer composed of 20 mM Tris pH 8, 250 mM NaCl, 1 mM $MgCl_2$, 0.5 mM TCEP and 5 % glycerol, 1x cOmplete™ ULTRA protease inhibitors and 12.5 U/ml Benzonaze. Cells were lysed by sonication followed by centrifugation at 21,000 g for 45 minutes at 4 ^{o}C. The supernatant was loaded onto a HisTrap FF column followed by on-column cleavage of the Trx-6His-HRV3C tag by addition of 2 mg of HRV-3C protease. The cleaved BCL6 5-129 BTB domain or Flag-TEV-BCL6 5-129 was then eluted and purified further by ResourceQ (for Flag-TEV-BCL6 construct only) and gel filtration using a HiLoad 26/60 Superdex75 column in a buffer containing 20 mM HEPES pH 7.5, 250 mM NaCl, 1 mM TCEP and 5 % glycerol. The final protein was assessed for purity and molar mass by SDS-PAGE and high-resolution mass spectrometry, respectively.

For the uncleaved Trx-6His-HRV3C-BCL6 protein construct to be used in TR-FRET, the protein was directly eluted from the HisTrap FF column without HRV-3C treatment, and submitted to Superdex75 gel filtration as described above.

1.4 BCL6 crystallisation

The purified BCL6 5-129 was crystallised in the presence of a tetra-peptide of sequence Ac-WVIP-NH_2. A stock solution of WVIP peptide at 100 mM in 100 % DMSO was added to a 2 mg/mL solution of purified BCL6 to a final concentration of 1 mM. This mixture was then concentrated to a final protein concentration of 4 mg/mL using a centrifugal concentrator with a 3 kDa molecular weight cut-off. Crystals were grown at 18 ^{o}C in hanging drops composed of 2 μL of the BCL6-BTB/WVIP complex plus 1 μL of a crystallisation solution consisting of 1 M K_2HPO_4, 0.7 M NaH_2PO_4, 75 mM sodium acetate buffer pH 4.5 and 2 % DMSO, against 350 μL of crystallisation solution. Crystals typically grew in 2 days, and compounds were soaked into crystals by addition of 0.5 μL of each compound (dissolved in DMSO to a final concentration of 10 to 200 mM) directly to crystallisation drops, followed by 10-120 minutes incubation. Crystals were then cryo-protected in a solution composed of the crystallisation reagent supplemented with 30 % ethylene glycol and cryo-cooled in liquid nitrogen.

The purified Flag-TEV-BCL6 5-129 was crystallised without any peptide, the supplementary Flag-TEV tag of this construct replacing the WVIP peptide in the crystal packing. The protein was concentrated to a final protein concentration of 10 mg/mL using a centrifugal concentrator with a 3 kDa molecular weight cut-off. Crystals were grown at 18 ^{o}C in hanging drops composed of 1.5 μL of the Flag-TEV-BCL6 complex plus 1.5 μL of a crystallisation solution consisting of 0.1 M Tris pH 7.5 and 0.80 M Na/K Tartrate, against 300 μL of crystallisation solution. Crystals typically grew in 2 days,

and compounds were soaked as described above for the other construct. Crystals were then cryo-protected in a solution composed of the crystallisation reagent supplemented with 30 % ethylene glycol and cryo-cooled in liquid nitrogen.

1.5 Crystallographic data collection, processing and refinement

X-ray data were collected at Diamond Light Source, Harwell campus, Oxfordshire, UK, on beamlines I03, and I04-1, or at ESRF, Grenoble, France, on beamline ID30A-1. Crystals obtained with both BCL6 constructs belonged to the space group P 6_1 2 2 and diffracted to between 1.44 and 1.81 Å resolution. Datasets were integrated with XDS[1] or DIALS[2] and scaled and merged with AIMLESS[3] or AutoPROC.[4] Structures were solved by molecular replacement using PHASER[5-6] with a publicly available BCL6 structure[7] (PDB code 3BIM) with ligand and water molecules removed used as molecular replacement model. All protein/ligand structures were manually corrected and rebuilt in COOT[8] and refined with BUSTER[9] in iterative cycles. Ligand restraints were generated with GRADE[10] and MOGUL[11]. The quality of the structures was assessed with MOLPROBITY[12-13]. The data collection and refinement statistics are presented in Supplementary Table S2.

2. Supplementary experimental: biological assay conditions

Cell lines were supplied by the German Collection of Microorganisms and Cell Cultures (DSMZ). Cell lines were authenticated by STR profiling using a GenePrint® 10 kit (Promega, Southampton, UK) and a 3730xl DNA analyser (Applied Biosystems, Warrington, UK). All STR profiles were >80% match (using ATCC or DSMZ matching algorithms) with the respective reference profile. Cells were routinely screened for *Mycoplasma*, using an in-house PCR-based assay (Universal Mycoplasma Detection Kit (30-1012K, ATCC, Manassas, VA, USA).

2.1 TR-FRET assay

Assays were performed in a 384-well black Proxiplate (Perkin Elmer) containing 1 nM* Trx-6xHis-BCL6 (in house-produced, human BCL6 BTB domain covering amino-acid sequence 5-129), 300 nM BCOR-AF633 peptide (RSEIISTAPSSWVVPGP-Cys-AlexaFluor 633-amide, Cambridge Research Biochemical) and 0.5 nM anti-6xHis-Terbium cryptate (CisBio Bioassays, France), in assay buffer (25 mM Hepes pH8, 100 mM NaCl, 0.05% Tween20, 0.5 mM TCEP, 0.05% bovine serum albumin). Test compounds in DMSO or DMSO alone were added to the wells using an ECHO550 acoustic dispenser (Labcyte Inc) to give the appropriate test concentration in 0.7% v/v DMSO final. After 2 hours incubation at room temperature the plate was read on an Envision plate reader (Perkin Elmer) with 337 nm laser excitation, a first emission filter APC 665 nm and a second emission filter Europium 615 nm, or alternatively on a Pherastar FSX (BMG Labtech) plate reader equipped with 337 nm laser excitation filter, a first emission filter at 620 nm and a second emission filter at 665 nm. The % inhibition at each concentration was calculated by normalising FRET ratio to the appropriate high (DMSO with all reagents) and low (DMSO without BCL6) controls. IC_{50} values were determined using GraphPad Prism 6.0 or Dotmatics (Bishops Stortford, UK) software by fitting the normalised data to a sigmoidal four-parameter logistic fit equation.

*10 nM was used for initial testing or early examples. Individual replicates shown in supplementary table S1.

2.2 NanoBRET assay

A cellular nano-Bioluminescence Resonance Energy Transfer (nanoBRET) assay (Promega NanoBRET Nano-Glo Detection System, catalogue number N1662) was used to detect inhibition of the BCL6-SMRT (also called NCOR2) corepressor protein-protein interaction. DNA encoding full length BCL6 and SMRT were inserted into pFC32K.NanoLuc and pFC14K.HaloTag vectors (Promega)

to produce C-terminal tagged fusion proteins BCL6-nanoLuc and SMRT-HaloTag, respectively. HEK293T cells were plated ($5x10^5$) in T75 tissue culture flask and bulk transfected 48 hours later with Fugene 6 (Promega cat# E2691) reagent and 18 µg total DNA plasmids encoding BCL6-nanoLuc as donor and SMRT-HaloTag as acceptor, at a donor:acceptor DNA ratio of 1:25. At 24 hr post-transfection, HEK293T cells were collected and stored in liquid nitrogen in 90% FBS (PAN Biotech UK) and 10% DMSO. At the time of assay, compounds (100nL/well) and NanoBRET 618 ligand (10nL of 1mg/ml stock solution per well) were dispensed in a dry 384-well NUNC white assay plate (ThermoScientific NUNC cat.#10080681) using Echo550 acoustic dispensing (Labcyte Inc.). Frozen transfected HEK293T cells were thawed, centrifuged and freezing medium was replaced by phenol red-free OptiMEM+4% FBS (Life Technology). The cell density was adjusted to $3x10^5$ cells/ml and 20 µL (6000 cells) were plated in each well containing test compounds (0.0125-50 µM) in DMSO or DMSO alone and 0.5 µg/ml NanoBRET 618 fluorescence ligand, in 0.55% v/v DMSO final concentration. Cells were incubated for 6 hr at 37 °C / 5% CO_2 then NanoBRET furimazine substrate (Promega) was added to give a final concentration of 10 µM. After a short centrifugation the plates were read on an Envision (Perkin Elmer) plate reader equipped with a LUM/D600 Dual mirror, Lum 450/40 nm bandpass and D605 nm longpass filters, with a 0.2 sec reading to determine the BRET ratio. Alternatively, plates were read on Pherastar FSX (BMG Labtech) equipped with BRET module LP610 nm (1st emission filter) / 450-80 nm (2nd emission filter). The % inhibition at each test concentration was calculated by normalising the BRET ratio to the appropriate high and low controls. The compound IC_{50}s were determined using Graphpad Prism 6.0 or Dotmatics software by fitting the normalised data to a sigmoidal four-parameter logistic fit equation.

2.3 Cell proliferation assay

Cells were seeded in 96-well culture plates at a density of 2500 cells/well in RPMI-1640 medium (Sigma-Aldrich) supplemented with 10% FBS (Gibco). Compounds were initially dispensed into 96-well U-bottom plates using an Echo 550 acoustic dispenser (Labcyte Inc.), then diluted in RPMI-1640 medium and transferred onto the cells. Cells were treated with 8 compound concentrations in duplicate, ranging from 1.07 nM to 10 µM, in a final DMSO concentration of 0.1% and final volume of 100 µl. Cells were incubated with compound for 14 days, with medium changes at days 3, 7 and 10 carried out as follows: fresh 96-well cell culture plates were prepared containing 100 µl medium plus compound at the assay concentrations (white plates were used on day 10 to optimise luminescence measurement). Assay plates containing cells were vortexed to mix and cell density in one control well was counted using a Coulter Z2 cell counter (Beckman Coulter). The volume of medium containing

2500 cells in the control well was calculated and this volume of cells was transferred from every well of the assay plates to the corresponding well of the fresh plates containing compound. After 14 days, CellTiter Glo reagent (Promega) was added to the medium in each well of the assay plate at a ratio of 1:2, mixed on a plate shaker, then incubated at room temperature for 10 minutes. Luminescence was measured using an Envision plate reader (Perkin Elmer) and the relative luminescence at each compound concentration, compared to DMSO alone, was calculated. GI50 were determined using a 4-parameter curve fit in Dotmatics (Bishops Stortford, UK).

3. Supplementary experimental: physicochemical assays

3.1 NMR solubility assay

9 μL of 10 mM DMSO stock solution was pipetted into one well of a 384 deepwell plate (Greiner, part-no. 781270), then 171 μL of HEPES buffer (20 mM HEPES [Sigma Aldrich, cat-no. H3375-250G], 150 mM NaCl, 0.5 mM TCEP, 10% D_2O) was pipetted into the same well and mixed by up-down pipetting 3 times to create a 0.5 mM solution or suspension containing 5% DMSO. It was then separated by centrifugation (1000 rpm for 1 min, Eppendorf 5810C). The plate was then sealed and incubated at room temperature for 20 hours, without shaking. The plate was centrifuged again for 1 minute at 1000 rpm on Eppendorf 5810R before 165 μL of the supernatant was transferred to a 3 mm NMR tube (Bruker, Part No. Z112272) using liquid handler SamplePro Tube SJ S (Bruker). The concentration of the solubilized compound in solubility sample is measured by quantitative ^{1}H-NMR using a single external standard (200 μM caffeine (Sigma, C1778) in PBS (pH 7.4) with 1% DMSO-d6).

The detail of the NMR method is as following: NMR data was collected on a Bruker Avance Neo 600 spectrometer equipped with a 5 mm TCI-CryoProbe. The ^{1}H spectrum was referenced to the internal deuterated solvent. The operating frequency for ^{1}H was 600 MHz. All NMR data were acquired at the temperature of 298 K. All data were acquired and processed using Bruker Topspin 4.0. The quantitative ^{1}H-NMR spectrum was acquired using a Bruker standard 1D lc1pngppsf2 pulse sequence with 32 scans. The sweep width was 6.2 ppm with O1P set to 8.8 ppm, and the FID contained 16k time-domain data points. Relaxation delay was set to 20 sec. Water signal was suppressed. The quantification of the compound concentration was carried out using MestReNova Mgear.[14-16]

3.2 HPLC solubility assay

10 µL of 10 mM DMSO stock solution was pipetted into a micro centrifuge tube (1.5 mL, Sarstedt part-no. 72.690.001) containing 990 µL of PBS buffer (pH 7.4, Sigma Aldrich, cat-no. 79382) and mixed for 5 seconds on vortex mixer (Grant-bio vortex mixer) to create 100 µM solution with 1% DMSO. Following shaking of the suspension on a Bohdan Shaker at 500 rpm for 2 hours at room temperature (20 °C), it was separated by centrifugation (14000 rpm for 15 min, Eppendorf 5415C). 200 µL of the supernatant was transferred to a 2 mL Agilent vial containing 50 µL of DMSO (Sigma Aldrich, cat-no. 41640-100ML) and mixed for 5 seconds to avoid precipitation from the saturated solution.

The concentration of the solubilized compound in solubility sample is measured by HPLC with UV detection using an external standard which was prepared by pipetting 10 µL of the same batch of compound DMSO stock used in solubility sample preparation to 990 µL of DMSO.

The detail of the HPLC method is as following: chromatographic separation at 30°C is carried out over a 5 minute gradient elution method from 90:10 to 10:90 water:methanol (both modified with 0.1% formic acid) at a flow rate of 1.5 mL/min. Calibration curve is prepared by injecting 0.5, 2.5, and 5 µL of compound external standard. Compound solubility value is obtained by injecting 6.25 and 62.5 µL of compound solubility sample.

4. <u>Supplementary information: synthetic and analytical methods</u>

4.1 General Synthetic Information

All anhydrous solvents and reagents were obtained from commercial suppliers and used without further purification. Evaporation of solvent was carried out using a rotary evaporator under reduced pressure at a bath temperature of up to 60 °C. Flash column chromatography was carried out using a Biotage purification system using prepacked SNAP KP-Sil cartridges. Reverse phase column chromatography was carried out using a Biotage purification system using Biotage SNAP Ultra C-18 12g and 30g columns as required. HPLC separations were carried out on an Agilent 6120 MS-Prep LC using an ACE 5 C18-PFP 250 x 21.2 mm column using a 15 min gradient of water:methanol (both modified with 0.1% formic acid). Microwave-assisted reactions were carried out using a Biotage Initiator microwave system. Final compounds were purified to ≥ 95% purity.

4.2 Analytical Methods

NMR spectra were recorded on a Bruker AMX 500 (500 MHz) spectrometer, or on a Bruker Avance NEO 600 (600 MHz) spectrometer equipped with 5 mm TCI Cryoprobe. NMR data is presented in the form of chemical shift δ (multiplicity, coupling constants, integration) for major diagnostic protons, given in parts per million (ppm) relative to tetramethylsilane (TMS), referenced to the internal deuterated solvent.

LCMS/HRMS analysis was performed according to one of four methods as described in the experimental text. Where **2min; ToF** is shown, LC/MS and HRMS analysis was performed on an Agilent 1200 series HPLC and diode array detector coupled to a 6210 time of flight mass spectrometer with dual multimode APCI/ESI source. Analytical separation was carried out at 40°C on a Merck Chromolith Flash column (RP-18e, 25 x 2 mm) using a flow rate of 1.5 mL/min in a 2 minute gradient elution with detection at 254 nm. The mobile phase was a mixture of methanol (solvent A) and water (solvent B), both containing formic acid at 0.1%. Gradient elution was as follows: 5:95 (A/B) to 100:0 (A/B) over 1.25 min, 100:0 (A/B) for 0.5 min, and then reversion back to 5:95 (A/B) over 0.05 min, finally 5:95 (A/B) for 0.2 min. Where **4min; ToF** is shown, the method is the same except at 30°C and using a flow rate of 0.75 mL/min in a 4 minute gradient elution as follows: 5:95 (A/B) to 100:0 (A/B) over 2.5 min, 100:0 (A/B) for 1 min, and then reversion back to 5:95 (A/B) over 0.1 min, finally 5:95 (A/B) for 0.4 min. Where **2min; ESI** is shown, LC/MS and HRMS analysis was performed on a Waters Acquity UPLC and diode array detector coupled to a Waters G2 QToF mass spectrometer fitted with a multimode ESI/APCI source. Analytical separation was carried out at 30°C on a Phenomenex Kinetex C18 column (30 x 2.1 mm, 2.6u, 100A) using a flow rate of 0.5 mL/min in a 2 minute gradient elution with detection at 254 nm. The mobile phase was a mixture of methanol (solvent A) and water (solvent B), both containing formic acid at 0.1%. Gradient elution was as follows: 10:90 (A/B) to 90:10 (A/B) over 1.25 min, 90:10 (A/B) for 0.5 min, and then reversion back to 10:90 (A/B) over 0.15 min, finally 10:90 (A/B) for 0.1 min. Where **4min; ESI** is shown, the method is the same except using a flow rate of 0.3 mL/min in a 4 minute gradient elution as follows: 10:90 (A/B) to 90:10 (A/B) over 3 min, 90:10 (A/B) for 0.5 min, and then reversion back to 10:90 (A/B) over 0.3 min, finally 10:90 (A/B) for 0.2 min.

5. Supplementary analytical data

5.1 HPLC/MS traces for key compounds

5.1.1 Compound 1

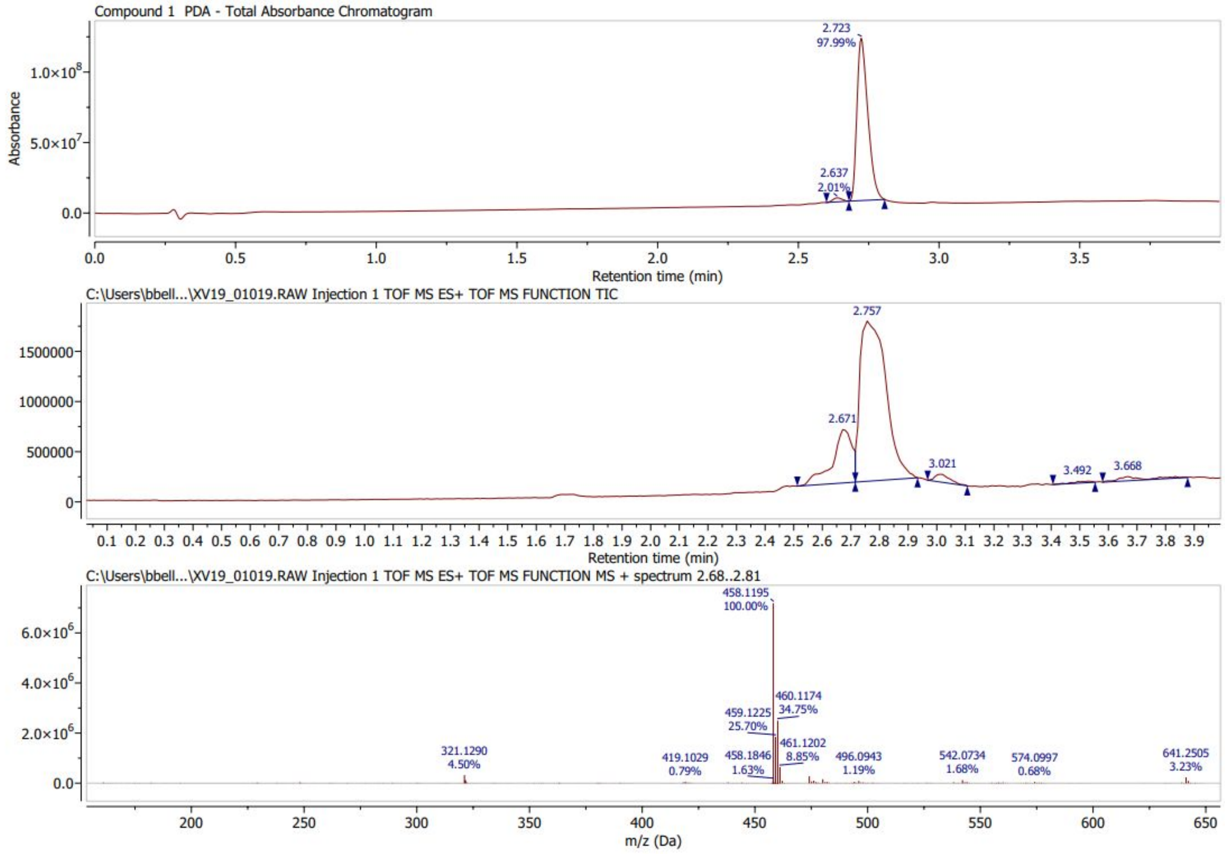

5.1.2 Compound 7

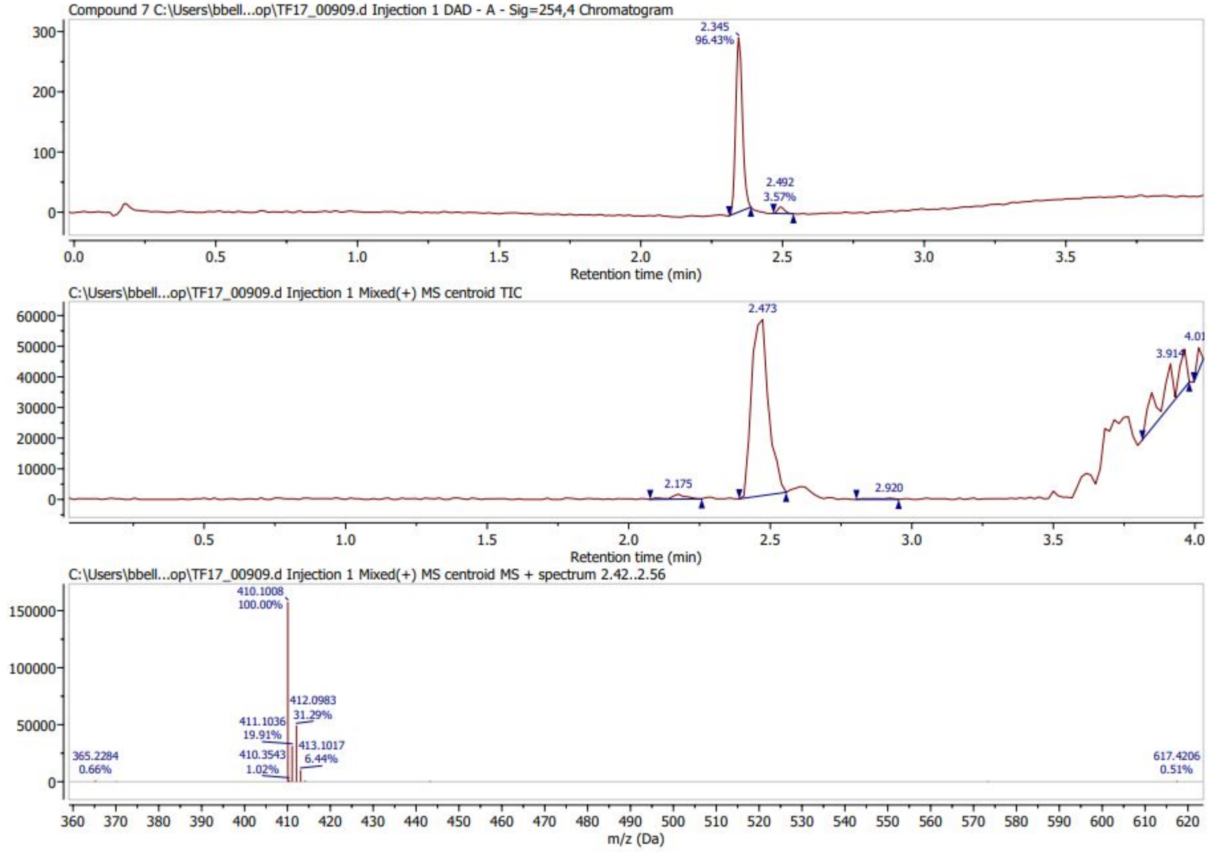

5.1.3 Compound 9a

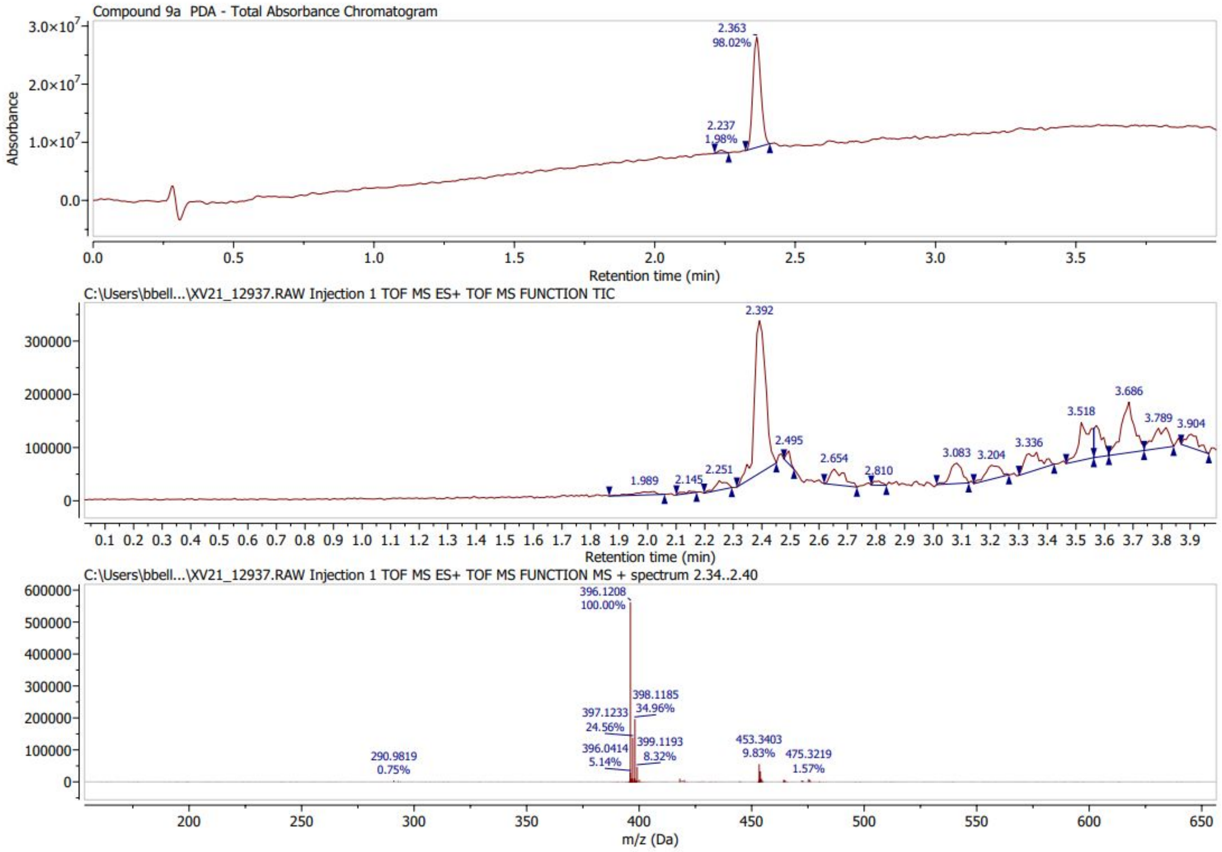

5.1.4 Compound 12a

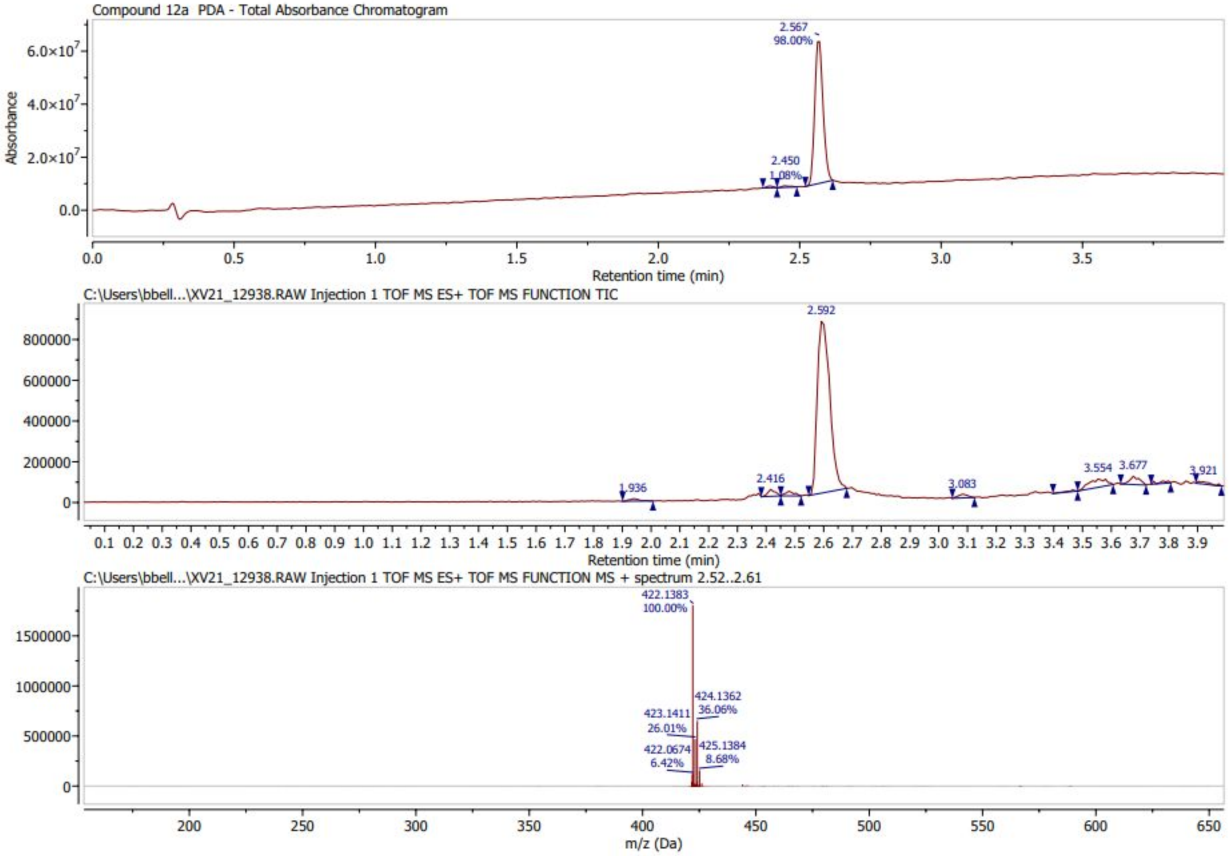

5.2 Chiral SFC traces

5.2.1 Compound 34a

Method QDa Methanol NH3 30%

Date Acquired 18/06/2019 14:26:15 BST

Column Name Amy-C

Data File ICR19 Final

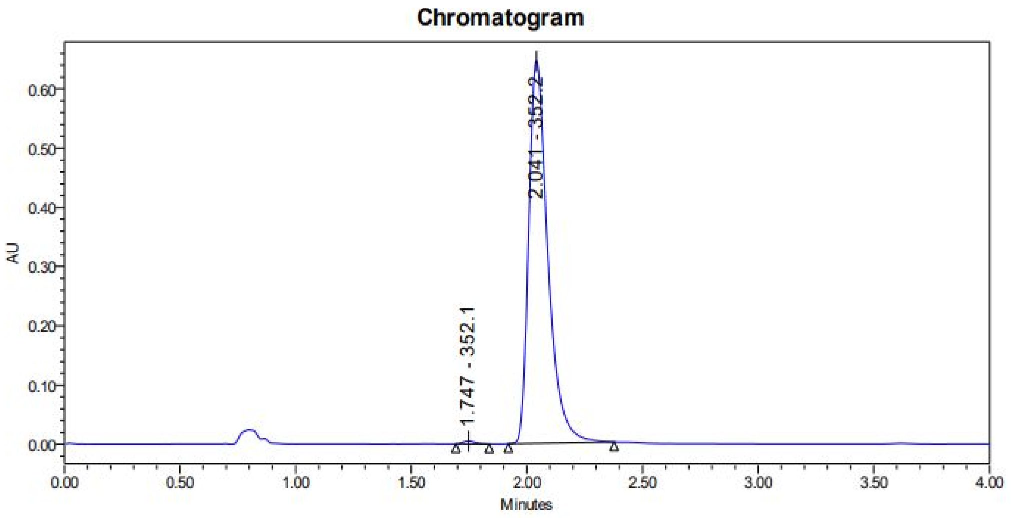

Peak Results

	Retention Time (min)	Area (µV*sec)	% Area	Width @ 50%
1	1.75	17505	0.5	0.05522
2	2.04	3713886	99.5	0.08501

5.2.2 **Compound 34b**

Method QDa Methanol NH3 30%

Date Acquired 19/06/2019 09:21:44 BST

Column Name Amy-C

Data File ICR19 Final

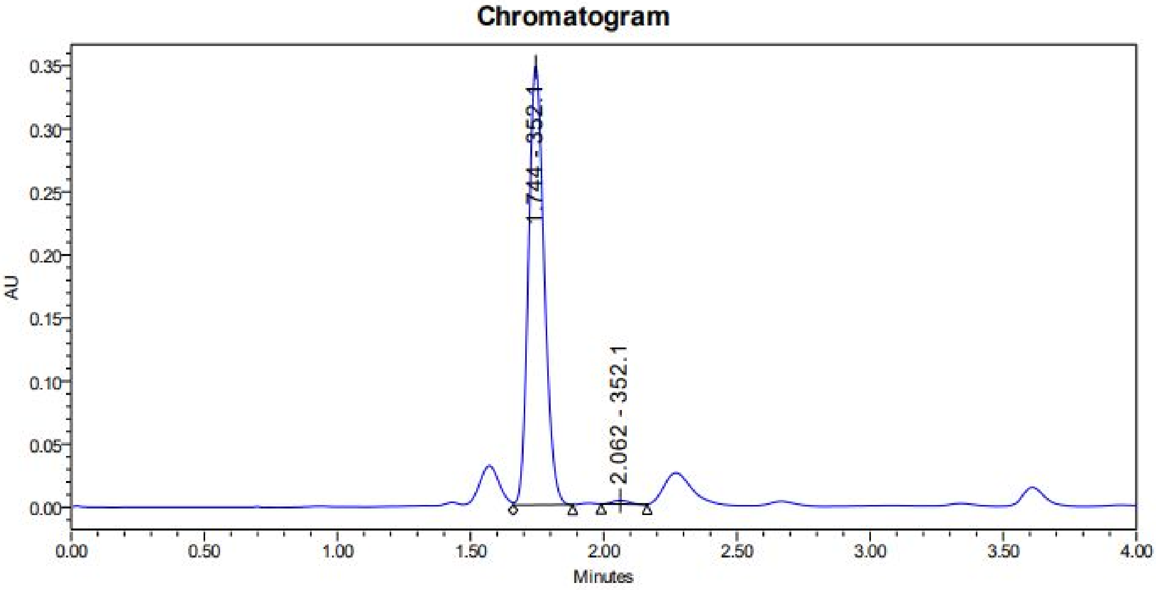

Peak Results

	Retention Time (min)	Area (µV*sec)	% Area	Width @ 50%
1	1.74	1429117	99.1	0.06357
2	2.06	12845	0.9	0.07140

6. Supplementary tables

Table S1: summary statistics and individual replicate values

No.	BCL6 TR-FRET IC_{50} Geometric Mean (μM)	BCL6 TR-FRET IC_{50} individual replicates (μM)	BCL6 TR-FRET pIC_{50} mean	BCL6 TR-FRET pIC_{50} SD	BCL6 TR-FRET pIC_{50} SEM	n	LE	LLE
**1**	0.0039	0.0029, 0.0024, 0.0162, 0.0029, 0.0040, 0.0039, 0.0029	8.40	0.28	0.11	7	0.37	5.40
**2**	1.307	*1.91*, 1.66, 1.95, 1.05, 0.68, 1.13	5.88	0.18	0.07	6	0.34	3.48
**3**	0.026	0.0255, 0.0331, 0.0410, 0.0226, 0.0156	7.58	0.16	0.07	5	0.30	4.58
**4**	5.581	*6.09, 5.57*, 4.59, 5.76, 6.04	5.25	0.05	0.02	5	0.28	2.15
**5a**	6.120	*7.06, 6.93*, 3.89, 7.37	5.21	0.13	0.07	4	0.29	3.11
**5b**	2.078	*1.03, 0.96*, 1.46, 1.67, 6.96, 4.82	5.68	0.36	0.15	6	0.29	2.88
**6**	1.332	1.11, 1.39, 1.53	5.88	0.07	0.04	3	0.30	3.78
**7**	0.094	*0.153, 0.151*, 0.098, 0.056, 0.058	7.03	0.21	0.10	5	0.34	5.93
**8**	2.239	2.19, 2.18, 2.35	5.65	0.02	0.01	3	0.28	3.95
**9a**	0.129	0.136, 0.103, 0.121, 0.165	6.89	0.09	0.04	4	0.34	4.39
**9b**	1.065	0.923, 1.12, 1.17	5.97	0.05	0.03	3	0.30	3.47

No.	BCL6 TR-FRET IC_{50} Geometric Mean (μM)	BCL6 TR-FRET IC_{50} individual replicates (μM)	BCL6 TR-FRET pIC_{50} mean	BCL6 TR-FRET pIC_{50} SD	BCL6 TR-FRET pIC_{50} SEM	n	LE	LLE
**9r**	0.547	0.669, 0.732, 0.421, 0.435	6.26	0.12	0.06	4	0.31	3.76
**10**	18.691	15.6, 22.4	4.73			2	0.25	2.83
**11a**	0.078	0.075, 0.069, 0.093	7.11	0.07	0.04	3	0.33	4.21
**11b**	0.612	0.454, 0.496, 1.02	6.21	0.19	0.11	3	0.30	3.21
**11c**	4.312	3.57, 5.26, 4.27	5.37	0.08	0.05	3	0.24	2.07
**11d**	3.119	2.41, 3.46, 3.64	5.51	0.10	0.06	3	0.26	2.21
**11e**	2.023	2.15, 1.54, 2.50	5.69	0.11	0.06	3	0.27	3.69
**12a**	0.039	0.0274, 0.0267, 0.0343, 0.0624, 0.0424, 0.0531	7.41	0.15	0.06	6	0.35	4.51
**12b**	1.119	0.852, 1.48, 1.11	5.95	0.12	0.07	3	0.28	3.05
**13a**	0.483	0.215, 0.628, 0.651, 0.618	6.32	0.23	0.12	4	0.30	2.92
**13b**	>9.8	8.7, 16.5, >6.6	<5.01			2		
**13c**	1.022	0.82, 1.55, 0.84	5.99	0.16	0.09	3	0.28	2.89
**13d**	1.588	1.07, 2.07, 1.87	5.80	0.15	0.09	3	0.27	2.90
**14**	0.494	0.615, 0.397	6.31			2	0.28	3.31

No.	BCL6 TR-FRET IC_{50} Geometric Mean (µM)	BCL6 TR-FRET IC_{50} individual replicates (µM)	BCL6 TR-FRET pIC_{50} mean	BCL6 TR-FRET pIC_{50} SD	BCL6 TR-FRET pIC_{50} SEM	n	LE	LLE
**BI-3812**	0.003	0.003	8.52	-	-	1	0.31	6.76
**BI-3802**	0.033	0.012, 0.051, 0.025, *0.107, 0.039,* 0.021	7.48	0.33	0.14	6	0.31	3.98

** for early examples, indicated in italics, TR-FRET assay was run using 10 nM BCL6 instead of 1 nM.*

LE calculated using 1.4(pIC50)/HAC. LLE calculated using MoKa* $logD_{7.4}$ *values.*

*Table S2: Crystallographic data collection and refinement statistics for **4**, **7** and **9a**.*

Crystal system	BCL6/WVIP	BCL6/WVIP	Flag-BCL6
Ligand	**4**	**7**	**9a**
PDB code	7Q7S	7Q7T	7Q7U
Crystal			
Space group	P 6_1 2 2	P 6_1 2 2	P 6_1 2 2
Unit cell dimensions (a/b/c in Å)	67.20/67.20/165.14	68.57/68.57/167.25	67.66/67.66/166.05
Unit cell angles (α/β/γ in °)	90/90/120	90/90/120	90/90/120
Data collection and processing			
Beamline	ESRF ID30A-1	DLS I04-1	DLS I03
Wavelength (Å)	0.9660	0.9282	0.9762
Integration program	XDS	DIALS	DIALS
Reduction program	AIMLESS	AIMLESS	AIMLESS
Resolution range	47.57 – 1.44	48.42 – 1.46	47.87 – 1.78
Number of unique reflections [a]	40751 (1993)	41379 (2005)	22461 (1212)
Completeness [a]	99.7 (99.8)	100 (99.9)	99.9 (98.7)
Redundancy [a]	11.1 (10.9)	12.4 (12.8)	24.5 (24.6)
R_{merge} (%) [a]	11.8 (207.3)	5.8 (224.2)	11.4 (289.5)
I/σ(I) [a]	12.9 (1.1)	17.1 (1.1)	15.6 (0.9)
$CC_{1/2}$ [a, b]	0.998 (0.367)	1.000 (0.541)	0.993 (0.714)
Refinement			
Program	BUSTER	BUSTER	BUSTER
R_{work} (%)	18.37	19.53	0.1979
R_{free} (%)	20.02	21.26	0.2283
Number of residues	131	131	131
Number of water molecules	207	191	150
Average B-factor (Å^2)	23.05	32.56	41.89
Ramachandran favoured (%)	96.8	97.6	98.5
Ramachandran outliers (%)	0	0	0
RMSD bonds (Å)	0.012	0.012	0.011
RMSD angles (°)	1.458	1.363	1.431

[a] Values between brackets are for the highest resolution shell.

[b] Half-dataset correlation coefficient, see: Karplus, P. A.; Diederichs, K. Linking crystallographic model and data quality. *Science* **2012**, *336*, 1030−1033.

*Table S3: Crystallographic data collection and refinement statistics for **12a** and **1**.*

Crystal system	Flag-BCL6	Flag-BCL6
Ligand	**12a**	**1**
PDB code	7Q7V	7Q7R
Crystal		
Space group	P 6_1 2 2	P 6_1 2 2
Unit cell dimensions (a/b/c in Å)	67.31/67.31/166.81	67.52/67.52/166.92
Unit cell angles (α/β/γ in °)	90/90/120	90/90/120
Data collection and processing		
Beamline	DLS I03	DLS I03
Wavelength (Å)	0.9763	0.9763
Integration program	XDS	DIALS
Reduction program	AIMLESS	AIMLESS
Resolution range	19.63 – 1.81	47.89 – 1.70
Number of unique reflections [a]	21275 (1230)	25720 (1337)
Completeness [a]	99.9 (100)	100 (100)
Redundancy [a]	23.1 (24.4)	36.9 (38.3)
R_{merge} (%) [a]	13.3 (259.3)	7.9 (251.4)
I/σ(I) [a]	13.4 (1.6)	24.1 (1.9)
$CC_{1/2}$ [a, b]	0.999 (0.514)	0.999 (0.813)
Refinement		
Program	BUSTER	BUSTER
R_{work} (%)	18.46	19.40
R_{free} (%)	20.65	20.58
Number of residues	131	130
Number of water molecules	92	159
Average B-factor (Å^2)	38.08	41.04
Ramachandran favoured (%)	98.5	98.4
Ramachandran outliers (%)	0	0
RMSD bonds (Å)	0.012	0.012
RMSD angles (°)	1.521	1.417

[a] Values between brackets are for the highest resolution shell.

[b] Half-dataset correlation coefficient, see: Karplus, P. A.; Diederichs, K. Linking crystallographic model and data quality. *Science* **2012**, *336*, 1030−1033.

Table S4: Optimisation of the cyclisation reaction forming 19

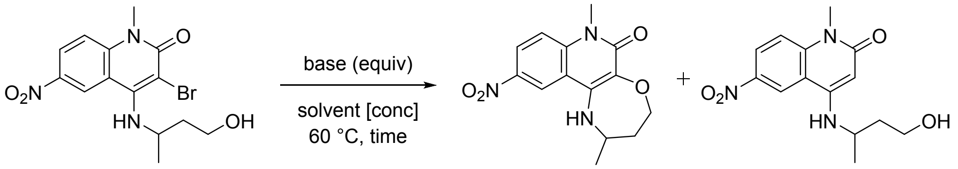

25c **19** **25a**

Base[a] (equiv)	Solvent [conc]	Time / min	Ratio 19 : 25a	Conversion[b] (isolated yield) / %
KO^{t}Bu (1.8)	DMSO [0.075]	50	76 : 24	91 (41)[c]
KO^{t}Bu (1.8)	DMSO [0.075]	60	81 : 19	78
NaO^{t}Bu (1.8)	DMSO [0.075]	60	76 : 24	85
LiO^{t}Bu (1.8)	DMSO [0.075]	60	83 : 17	100
LiOMe (1.8)	DMSO [0.075]	60	-	<5[d]
LiO^{t}Bu (1.8)	DMF [0.075]	60	93:7	100
LiO^{t}Bu (1.8)	DME [0.075]	60	100 : 0	90
LiO^{t}Bu (1.8)	DCE [0.075]	60	100 : 0	30
LiO^{t}Bu (1.8)	THF [0.075]	60	100 : 0	100 (79)
LiO^{t}Bu (1.6)	THF [0.075]	60	100 : 0	100
LiO^{t}Bu (1.4)	THF [0.075]	60	100 : 0	80
LiO^{t}Bu (1.6)	THF [0.1]	60	100 : 0	100
LiO^{t}Bu (1.6)	THF [0.1]	15	100 : 0	100 (81)

a) KOtBu and LiOtBu used as 1 M solutions in THF. Other bases and additives including LiOH, DIPEA, imidazole, triazabicyclodecene, cesium carbonate, HCl or TFA were attempted but did not lead to product formation; b) 0.07 mmol scale unless stated; c) 0.3 mmol scale, reaction conducted using microwave irradiation; d) multiple side products observed.

7. References for supplementary information

1. Kabsch, W., XDS. Acta Crystallographica Section D 2010, 66 (2), 125-132.

2. Winter, G.; Waterman, D. G.; Parkhurst, J. M.; Brewster, A. S.; Gildea, R. J.; Gerstel, M.; Fuentes-Montero, L.; Vollmar, M.; Michels-Clark, T.; Young, I. D.; Sauter, N. K.; Evans, G., DIALS: implementation and evaluation of a new integration package. Acta Crystallographica Section D 2018, 74 (2), 85-97.

3. Evans, P., Scaling and assessment of data quality. Acta Crystallographica Section D 2006, 62 (1), 72-82.

4. Vonrhein, C.; Flensburg, C.; Keller, P.; Sharff, A.; Smart, O.; Paciorek, W.; Womack, T.; Bricogne, G., Data processing and analysis with the autoPROC toolbox. *Acta Crystallographica Section D* **2011,** *67* (4), 293-302.

5. McCoy, A. J.; Grosse-Kunstleve, R. W.; Adams, P. D.; Winn, M. D.; Storoni, L. C.; Read, R. J., Phaser crystallographic software. Journal of Applied Crystallography 2007, 40 (4), 658-674.

6. Winn, M. D.; Ballard, C. C.; Cowtan, K. D.; Dodson, E. J.; Emsley, P.; Evans, P. R.; Keegan, R. M.; Krissinel, E. B.; Leslie, A. G. W.; McCoy, A.; McNicholas, S. J.; Murshudov, G. N.; Pannu, N. S.; Potterton, E. A.; Powell, H. R.; Read, R. J.; Vagin, A.; Wilson, K. S., Overview of the CCP4 suite and current developments. Acta Crystallographica Section D 2011, 67 (4), 235-242.

7. Ghetu, A. F.; Corcoran, C. M.; Cerchietti, L.; Bardwell, V. J.; Melnick, A.; Privé, G. G., Structure of a BCOR Corepressor Peptide in Complex with the BCL6 BTB Domain Dimer. Molecular Cell 2008, 29 (3), 384-391.

8. Emsley, P.; Cowtan, K., Coot: model-building tools for molecular graphics. Acta Crystallographica Section D 2004, 60 (12 Part 1), 2126-2132.

9. G. Bricogne, E. B., M. Brandl, C. Flensburg, P. Keller, W. Paciorek, P. Roversi,; A. Sharff, O. S. S., C. Vonrhein, T.O. Womack, BUSTER, Version 2.10.2, Global Phasing Ltd., Cambridge, United Kingdom 2015.

10. O.S. Smart, T. O. W., A. Sharff, C. Flensburg, P. Keller, W. Paciorek,; C. Vonrhein, G. B., Grade, Version 1.2.9, Global Phasing Ltd., Cambridge, United Kingdom, 2014.

11. Bruno, I. J.; Cole, J. C.; Lommerse, J. P. M.; Rowland, R. S.; Taylor, R.; Verdonk, M. L., IsoStar: A library of information about nonbonded interactions. Journal of Computer-Aided Molecular Design 1997, 11 (6), 525-537.

12. Chen, V. B.; Arendall, W. B., III; Headd, J. J.; Keedy, D. A.; Immormino, R. M.; Kapral, G. J.; Murray, L. W.; Richardson, J. S.; Richardson, D. C., MolProbity: all-atom structure validation for macromolecular crystallography. Acta Crystallographica Section D 2010, 66 (1), 12-21.

13. Davis, I. W.; Leaver-Fay, A.; Chen, V. B.; Block, J. N.; Kapral, G. J.; Wang, X.; Murray, L. W.; Arendall, W. B., III; Snoeyink, J.; Richardson, J. S.; Richardson, D. C., MolProbity: all-atom contacts and structure validation for proteins and nucleic acids. Nucleic Acids Research 2007, 35 (suppl_2), W375-W383.

14. Wider, G.; Dreier, L., Measuring Protein Concentrations by NMR Spectroscopy. Journal of the American Chemical Society 2006, 128 (8), 2571-2576.

15. Lin, M.; Tesconi, M.; Tischler, M., Use of 1H NMR to facilitate solubility measurement for drug discovery compounds. International Journal of Pharmaceutics 2009, 369 (1), 47-52.

16. Saal, C.; Petereit, A. C., Optimizing solubility: Kinetic versus thermodynamic solubility temptations and risks. European Journal of Pharmaceutical Sciences 2012, 47 (3), 589-595.
